# Intrinsic and Measured Information in Separable Quantum Processes

**DOI:** 10.3390/e27060599

**Published:** 2025-06-03

**Authors:** David Gier, James P. Crutchfield

**Affiliations:** Complexity Sciences Center and Physics and Astronomy Department, University of California at Davis, One Shields Avenue, Davis, CA 95616, USA; dgier@formerstudents.ucdavis.edu

**Keywords:** quantum stochastic process, quantum information theory, classical information theory, hidden Markov models, information source, measurement, positive operator-valued measure, adaptive measurement, von Neumann entropy rate, Shannon entropy rate, excess entropy, computational mechanics, non-Markovian process, mutual information, synchronization, quantum process tomography, unifilarity

## Abstract

Stationary quantum information sources emit sequences of correlated qudits—that is, structured quantum stochastic processes. If an observer performs identical measurements on a qudit sequence, the outcomes are a realization of a classical stochastic process. We introduce quantum-information-theoretic properties for separable qudit sequences that serve as bounds on the classical information properties of subsequent measured processes. For sources driven by hidden Markov dynamics, we describe how an observer can temporarily or permanently synchronize to the source’s internal state using specific positive operator-valued measures or adaptive measurement protocols. We introduce a method for approximating an information source with an independent and identically distributed, Markov, or larger memory model through tomographic reconstruction. We identify broad classes of separable processes based on their quantum information properties and the complexity of measurements required to synchronize to and accurately reconstruct them.

## 1. Introduction

Determining a quantum system’s state requires grappling with multiple sources of uncertainty, including several that do not arise in classical physics. Irreducible limits on measurement, in particular, have been a hallmark of quantum physics since Heisenberg introduced the position–momentum uncertainty principle in 1927 [1]. Similar incompatible measurements exist for generic pure quantum states [2].

For a 2-level quantum system or *qubit*, it is impossible to simultaneously measure the value of a spin in the *x*, *y*, and *z* directions. (Stated mathematically, the Pauli matrices $\sigma_{x}$, $\sigma_{y}$, and $\sigma_{z}$ do not commute). Additionally, a single measurement in each basis is insufficient. One must measure many copies in each basis to specify the distribution of outcomes. As a result, determining an unknown qubit state through quantum state tomography requires measuring a large ensemble of identical copies with a set of mutually unbiased bases [3] or a single informationally complete *positive operator-valued measure* (POVM) [4].

These sources of uncertainty are familiar in quantum physics. Contrast them with when an observer receives a sequence of correlated qubits. Measuring them one by one, what will they see? And, what then can they infer about the resources necessary to generate these qubit strings? The following answers these questions by teasing apart the sources of apparent randomness and correlation in measured quantum processes.

### 1.1. Quantum and Classical Randomness

Also in 1927, von Neumann formulated quantum mechanics in terms of statistical ensembles and quantified the entropy of these *mixed quantum states*. In doing so, he extended Gibbs’ work on statistical ensembles and classical thermodynamic entropies to the quantum domain [5]. A mixed quantum state ρ has an entropy S(ρ)=−tr(ρlogρ), which is now known as the *von Neumann entropy.* (tr(·) is the trace operator). S(ρ)=0 if and only if ρ is a pure (nonmixed) quantum state. On the one hand, the von Neumann entropy is key to understanding quantum systems, particularly those with *entangled* subsystems that exhibit nonclassical correlations. On the other, the uncertainty S(ρ) quantifies a generic feature of statistical ensembles. It does not correspond to any particular quantum mechanical effect.

These two forms of uncertainty—due to ensembles and to quantum indeterminacy—are combined within the framework of quantum information theory, which generalizes classical information theory to quantum observables [6]. One notable example is noiseless coding. Shannon quantified the information content produced by a noiseless classical independent and identically distributed (i.i.d.) information source—one that emits a state drawn from the same distribution at each timestep [7]. Schumacher’s quantum noiseless coding theorem generalized this to quantum information sources. This gave a new physical interpretation of the von Neumann entropy: For an i.i.d. quantum source emitting state ρ, S(ρ) is the number of qubits required for a reliable compression scheme [8].

### 1.2. Sources with Memory

Non-i.i.d. stationary information sources inject additional forms of uncertainty. For example, a source may have an internal memory that induces correlations between sequential qubits and therefore between measurement outcomes. Such correlations may be purely classical or uniquely quantal in nature. As we will show, an experimenter who assumes (incorrectly) that such a source is i.i.d. and then applies existing tomographic methods will not detect these correlations and so will overestimate the source’s randomness and underestimate its compressibility.

Classical memoryful sources are described within the framework of computational mechanics, in which stationary dynamical systems serve as information sources with their own internal states and dynamic [9]. Sequential finite-precision measurements of a dynamical system form a discrete-time stochastic process. The resulting process’ statistics allow one to construct a model of the source and calculate its asymptotic entropy rate, internal memory requirements, and other physically relevant properties [10,11]. Importantly, the uncertainty associated with sequential measurements of a classical information source can be reduced, sometimes substantially, by an observer capable of synchronizing to the source’s internal states [12,13].

Subjecting an open quantum system to sequential qudit probes presents a similar but more general challenge, as the amount of information an observer can glean from an individual qudit through measurement is limited. Recent results established that applying particular measuring instruments to qubits induces complex behavior in measurement sequences [14]. Here, we extend these results by studying the properties of the quantum states themselves in addition to particular sequences of measurement outcomes.

The following introduces novel quantum-information-theoretic properties for sequences of separable—i.e., nonentangled—qudits. We build on previous results that focused on entropy rates, compression limits, and optimal coding strategies for stationary quantum information sources [15,16,17], as well as on results for specific experimentally motivated deviations from the i.i.d. assumption [18]. The approach is distinct from but complements recent efforts on quantum stochastic processes in which an observer measures a quantum system directly. This is complicated due to the latter’s interaction with an inaccessible environment that induces memory effects in sequential measurement outcomes [19,20,21,22,23].

Section 2 introduces classical processes, separable qudit processes, and methods of transforming from one to the other via classical-quantum channels and measurement channels. Then, Section 3, in concert with Appendix A, defines the entropies associated with quantum and classical processes, respectively. Adapting Ref. [11]’s entropy hierarchy, we employ discrete-time derivatives and integrals to obtain a family of distinct quantitative measures of quantum process randomness and correlation. We prove that, for projective or informationally complete measurements, the sequences of measurement outcomes form classical processes whose information properties are bounded by those of the quantum process being measured.

Section 4 then surveys examples of increasingly structured separable qubit and qutrit processes. Section 5 discusses how an observer can synchronize to a memoryful source—i.e., determine its internal state—through sequential measurement. Section 6 uses the resulting catalog of possible process behaviors to answer practical questions for an observer of a quantum process attempting to perform tomography. Finally, Section 7 draws out lessons and proposes future directions and applications, most notably extending the results to the experimentally realizable generation of arbitrary entangled qudit states [24,25] and using correlations as a resource to perform thermodynamic quantum information processing [26,27].

## 2. Stochastic Processes

**Figure 1:**
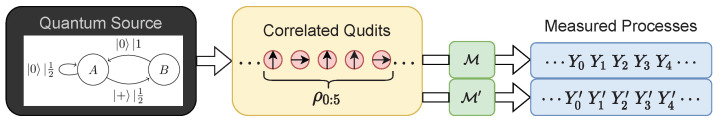
A stationary quantum information source emits qudits that are correlated due to the source’s internal memory. An experimenter measures these qudits in different ways (M or $M^{\prime }$), resulting in a family of classical stochastic processes.

We consider the output of an information source to be a discrete-time, stationary stochastic process. If the source output is a classical random variable—$X_{t}$ for each timestep *t*—we can directly apply the methods of computational mechanics [11]. Our goal is to extend these methods to describe separable sequences of qudits, each represented by a pure state in *d*-dimensional Hilbert space: $|\psi_{t}\rangle \in H^{d}$ at each timestep *t*. Given such a qudit sequence, one can perform repeated, identical measurements such that the outcomes form a classical stochastic process. Since one can choose to measure qudit states in many different bases, the properties of the classical measured process are determined by both the state of the correlated qudits and the measurement choice. Thus, the relationship between a quantum process and classical measured processes is one-to-many. Figure 1 illustrates this setup.

### 2.1. Classical Processes

A *classical stochastic process* is defined by a probability measure $\mathrm{Pr}(\overset{↔}{X})$ over a chain of random variables:

$$\begin{array}{c} \overset{↔}{X}\equiv X_{-2}X_{-1}X_{0}X_{1}X_{2}\ldots \;, \end{array}$$

with each $X_{t}$ taking on values drawn from a finite alphabet X. A block of *ℓ* consecutive random variables is denoted as $X_{0:l}=X_{0}X_{1}\cdots X_{l-1}$. The indexing is left-inclusive and right-exclusive. A particular bi-infinite process *realization* is denoted as $\overset{↔}{x}\equiv \ldots x_{-2}x_{-1}x_{0}x_{1}x_{2}\ldots$, with events $x_{t}$ taking values in a discrete set X. Realizations of a block of length *ℓ* are known as *words* and denoted as $x_{0:l}$. The set of all words of length *ℓ* is $X^{l}$.

We consider processes that are *stationary*, meaning that word probabilities $\mathrm{Pr}(X_{0:l})$ are time-independent:

$$\begin{array}{c} \mathrm{Pr}\left(X_{0:l}\right)=\mathrm{Pr}\left(X_{t:t+l}\right)\;, \end{array}$$

for all t∈Z and $l\in Z^{+}$. A stationary process’s statistics are fully described by the set of length-*ℓ* word distributions $\mathrm{Pr}(X_{0:l})$. A block of length *ℓ* has at most ${\left|X\right|}^{l}$ possible realizations (words).

One important subclass of processes are *independently and identically distributed* (i.i.d.) processes. The joint block probabilities of an i.i.d. process take the form

(1) $$\begin{array}{c} \mathrm{Pr}\left(X_{0:l}\right)\equiv \mathrm{Pr}\left(X_{0}\right)\mathrm{Pr}\left(X_{1}\right)\cdots \mathrm{Pr}\left(X_{l-1}\right)\;, \end{array}$$

for all $l\in Z^{+}$. This factoring of the block probabilities results in no statistical correlations between any random variables due to stationarity $\mathrm{Pr}\left(X_{t}\right)=\mathrm{Pr}\left(X_{0}\right)$ for all *t* for an i.i.d. process.

Another commonly studied subclass consists of the *Markov processes* for which the distribution for each $X_{t}$ depends only on the immediately preceding random variable $X_{t-1}$. For Markov processes, the joint probabilities for finite-length blocks factor as

(2) $$\begin{array}{c} \mathrm{Pr}\left(X_{0:l}\right)\equiv \mathrm{Pr}\left(X_{0}\right)\mathrm{Pr}\left(X_{1}|X_{0}\right)\cdots \mathrm{Pr}\left(X_{l-1}|X_{l-2}\right)\;, \end{array}$$

where Pr(X|Y) is the probability distribution of random variable *X* conditioned on random variable *Y*.

Finally, there is the markedly larger subclass of *hidden Markov processes* that have an internal Markov dynamic that is not directly observable. Though the joint probabilities do not factor as in Equation (2), the internal Markov dynamic restricts the process statistics, as we describe next.

### 2.2. Presentations

A *presentation* of a process is a model consisting of a set of internal states and a transition dynamic between those states that together reproduce the process’s statistics exactly. A given process may have many presentations. We focus on those depicted with state transition diagrams (directed graphs) that generate stationary, discrete-time stochastic processes in a natural way.

A *Markov chain* is a process presentation defined by the pair (X,T):

- A finite alphabet X of *m* symbols x∈X.
- A m×m transition matrix *T*. That is, if the source emits symbol $x_{i}$, with probability $T_{ij}=\mathrm{Pr}\left(x_{j}|x_{i}\right)$, it emits symbol $x_{j}$ next.

The stationary distribution for a Markov chain is denoted as π, and it is a distribution over internal states in X that satisfies π=πT. For a Markov chain, the set of internal states is exactly the set of emitted symbols, since the probability distribution for the next symbol is completely determined by the previous symbol. We represent each state as a node in a graph and each transition as a directed edge between nodes labeled by the associated probability.

Markov chains are sufficient to represent Markov processes, but we can describe the more general class of hidden Markov processes by allowing for internal states that are not directly observable. These processes are generated by *hidden Markov chains* (HMCs), which are defined by the triple (S,X,T):

- A finite set $S=\{\sigma_{1},\ldots ,\sigma_{n}\}$ of internal states.
- A finite alphabet X of *m* symbols x∈X.
- A set $T=\{T^{x}:x\in X\}$ of *m* n×n symbol-labeled transition matrices. That is, if the source is in state $\sigma_{i}$, with probability $T_{ij}^{x}=\mathrm{Pr}\left(x,\sigma_{j}|\sigma_{i}\right)$, it emits symbol *x* while transitioning to state $\sigma_{j}$.

We represent each possible transition between states as an edge between their nodes labeled with the emitted symbol and the transition probability. An HMC’s stationary distribution π over S uniquely satisfies $\pi =\pi \sum_{x}T^{x}$.

Any HMC that exactly reproduces a process’s statistical features is a *generative* HMC. This is an important distinction, since only some of those also belong to the more restrictive class of *predictive* HMCs. An HMC is predictive if its state at time t+1 is completely determined by the state at time *t* and the emitted symbol. This property is known as *unifilarity*.

At this point, we must emphasize the difference between a process and a particular presentation of that process. This distinction is critical when designating processes and models to be ‘classical’ or ‘quantum.’ A discrete-time classical stochastic processes is classical because it consists of a chain of *classical* random variables. Markov chains and HMCs are classical models because their internal states and dynamics are both *classical*. One may instead construct a presentation of a classical process with a set of *quantum* states that the model transitions between via some *quantum* dynamic. An observer can recover the classical process’ statistics by taking sequential measurements on either the system or on ancilla qudits that interact with the system at each timestep. The simulation of classical stochastic processes with quantum resources is the objective of *quantum computational mechanics*. There, a class of quantum models (q-simulators) shows advantage in terms of memory requirements over provably minimal classical predictive models (ε-machines) [28,29,30,31,32]. Likewise, different presentations of a quantum processes may have an underlying dynamic that is either classical or quantum. We turn now to quantum processes and their presentations.

### 2.3. Quantum Processes

Discrete-time classical stochastic processes consist of one classical random variable for each timestep. Likewise, discrete-time quantum stochastic processes consist of one quantum state $|\psi_{t}\rangle \in H^{d}$ at each timestep. We first describe an i.i.d. quantum information source and then generalize to sources with memory.

#### 2.3.1. Memoryless

A discrete-time *quantum information source* emits a *d*-level quantum system or *qudit* at each timestep. The statistical mixture of the infinite qudit sequences emitted by a source is a *quantum process*. As in the classical setting, different classes of quantum processes are distinguished by their temporal correlations. Now, however, for quantum sources we must use quantum information theory to account for both classical and quantal correlations.

First, consider the output of an i.i.d. (memoryless) quantum information source. Let H be a *d*-dimensional Hilbert space with pure states $|\psi_{x}\rangle \in H$. A *d*-level i.i.d. quantum information source consists of a set Q of pure qudit states and a probability distribution over those states such that $\mathrm{Pr}(|\psi_{x}\rangle )>0$ for all $|\psi_{x}\rangle \in Q$. We refer to Q as a quantum alphabet and consider only quantum alphabets with a finite number of pure states.

At each discrete timestep *t*, the source emits state $|\psi_{x}\rangle$ with probability $\mathrm{Pr}(|\psi_{x}\rangle )$. The resulting ensemble is described by the d×d density matrix:

(3) $$\begin{array}{c} \rho_{iid}=\sum_{|\psi_{x}\rangle \in Q}\mathrm{Pr}\left(|\psi_{x}\rangle \right)|\psi_{x}\rangle \langle \psi_{x}|\;. \end{array}$$

This particular pure-state decomposition of $\rho_{iid}$ is not unique. Moreover, an observer cannot determine Q through observations—unless Q consists of only one state—since many pure-state ensembles correspond to the same density matrix.

If an i.i.d. source emits $\rho_{iid}$ at each timestep, then the quantum process generated by the source is simply the infinite tensor product state:

(4) $$\begin{array}{c} |\Psi_{iid}\rangle =\cdots ⊗\rho_{iid}⊗\rho_{iid}⊗\rho_{iid}⊗\cdots \;. \end{array}$$

#### 2.3.2. Memoryful

We cannot describe non-i.i.d. sources using a single probability distribution over Q but must introduce a probability distribution over *sequences* of states drawn from Q. We do this by associating each element of Q with an element in the symbol alphabet X of an underlying classical stochastic process $\overset{↔}{X}$. Infinite qudit sequences then inherit probabilities from $\overset{↔}{X}$. This construction results in qudit sequences that are *separable*—i.e., not entangled.

We express the relationship between symbols and pure quantum states via a memoryless classical-quantum channel E:X→H, taking $x\to |\psi_{x}\rangle$. This is also known as a *preparation channel* (or encoder); see Appendix B for more.

Preparation channels are dual to measurement channels, described later, that map quantum states to classical probability distributions and, via sampling, to particular symbols.

For the classical process $\overset{↔}{X}$ whose realizations consist of symbols x∈X, the associated quantum alphabet Q is constructed by passing each element of X through E such that Q={E(x)}. Thus, Q is completely determined by X and E. For example, in the i.i.d. case, Equation (3) can also be written as $\rho_{iid}=E\left(X\right)$, and each possible pure-state decomposition of $\rho_{iid}$ can now be interpreted as a different combination of classical random variable *X* and preparation channel E.

In a slight abuse of notation, we write $|\Psi_{-\infty :\infty }\rangle =E(\overset{↔}{X})$ to indicate that quantum process $|\Psi_{-\infty :\infty }\rangle$ is formed by passing each random variable of $\overset{↔}{X}$ through the classical quantum channel E.

Note that an infinite qudit sequence (separable or entangled) can be viewed as a one-dimensional lattice of qudits indexed by t∈Z. These possibly entangled states can be described in full generality using an operator algebraic approach. We ground our formal definition of quantum processes in this mathematical setting. (Reference [17] provides a more detailed treatment of observable algebras for entangled qudit sequences over Z).

Let $B_{t}$ be the *d*-dimensional matrix algebra describing all possible observables on lattice site *t*. (For d=2, the space of observables is spanned by the identity and the 2×2 complex Pauli (Hermitian, unitary) matrices). The state of the qudit at site *t* can be described by the density matrix $\rho_{t}$ acting on $H_{t}$ of dimension *d*. For a block of *ℓ* consecutive qudits, all observables can be described by the joint algebra over *ℓ* sites of the lattice, $B_{0:l}={⨂}_{t=0}^{l-1}B_{t}$, and the state of this block is $\rho_{0:l}$ acting on $H_{0:l}={⨂}_{t=0}^{l-1}H_{t}$, a Hilbert space of dimension $d^{l}$. Combining all local algebras allows one to define an algebra B over the infinite lattice. A quantum process is a particular state over the infinite lattice and can be written as $|\Psi_{-\infty :\infty }\rangle$.

As a necessary first step and to more readily adapt information-theoretic tools from classical processes, we return to the more restricted case: separable sequences of qudits drawn from a finite alphabet Q of pure qudit states. Given a classical word $w=x_{0}x_{1}\ldots x_{l-1}\in X^{l}$ and a preparation channel E:X→Q, a qudit sequence takes the form

(5) $$\begin{array}{cc} |\psi_{w}\rangle & =E(w)\\ \; & =|\psi_{x_{0}}\rangle ⊗|\psi_{x_{1}}\rangle ⊗\cdots ⊗|\psi_{x_{l-1}}\rangle \;, \end{array}$$

where *ℓ* is the length of the sequence, and $|\psi_{w}\rangle \in H_{0:l}$. Note that $\dim\left(H_{0:l}\right)=d^{l}$, Q={E(x)}, and the number of possible qudit sequences of length *ℓ* is ${\left|Q\right|}^{l}$ or—assuming all $|\psi_{x}\rangle$ are distinguishable—${\left|X\right|}^{l}$.

A *separable quantum process* is then defined by $|\Psi_{-\infty :\infty }\rangle =E(\overset{↔}{X})$. Different preparations—i.e., different combinations of $\overset{↔}{X}$ and E—may produce the same quantum process.

The set of length-*ℓ*-block density matrices for a quantum process is given by

(6) $$\begin{array}{cc} \rho_{0:l} & =E(X_{0:l})\\ \; & =\sum_{w\in X^{l}}\mathrm{Pr}\left(|\psi_{w}\rangle \right)|\psi_{w}\rangle \langle \psi_{w}|\;, \end{array}$$

where $|\psi_{w}\rangle$ are the separable vectors given in Equation (5). Conveniently, their probabilities are determined by those of the underlying classical stochastic process $\mathrm{Pr}(\overset{↔}{X})$: $\mathrm{Pr}(|\psi_{w}\rangle )=\mathrm{Pr}(w)$. Each $\rho_{0:l}$ is a finite subsystem of the pure quantum state $|\Psi_{-\infty :\infty }\rangle$ over the infinite lattice. We use left-inclusive/right-exclusive indexing for density matrices as well.

For a given $\rho_{0:l}$, one can also obtain a purification in a finite-dimensional Hilbert space [33]. It is important to note that, since $\rho_{0:l}$ does not have a unique pure-state decomposition, one cannot generally reconstruct the probabilities $\mathrm{Pr}(|\psi_{w}\rangle )$ from it. Rather, $\rho_{0:l}$ contains only information accessible to an observer. And, if Q contains nonorthogonal qudit states ($\langle \psi_{x}|\psi_{x^{\prime }}\rangle \neq 0$ for some $|\psi_{x}\rangle$,$|\psi_{x^{\prime }}\rangle \in Q$), then an observer cannot unambiguously distinguish them.

In addition to separability, we also focus on *stationary* quantum processes, meaning

(7) $$\begin{array}{c} \rho_{0:l}=\rho_{t:l+t}\;, \end{array}$$

for all $l\in Z^{+}$ and t∈Z. If $\overset{↔}{X}$ is stationary, then $|\Psi_{-\infty :\infty }\rangle =E(\overset{↔}{X})$ will be stationary by construction.

For an i.i.d. quantum process, the joint probabilities of $\overset{↔}{X}$ factor as in Equation (1), giving the quantum process the form of Equation (4). The length-*ℓ*-block density matrix is represented by a product state as follows:

(8) $$\begin{array}{c} \rho_{0:l}=\overset{l-1}{\underset{t=0}{⨂}}\rho_{iid}\;, \end{array}$$

with $\rho_{iid}$ taking the form in Equation (3).

For an underlying classical process $\overset{↔}{X}$ that is Markov, there are additional subtleties. Joint probabilities of $\overset{↔}{X}$ factor as in Equation (2), so the joint probabilities of $|\Psi_{-\infty :\infty }\rangle =E(\overset{↔}{X})$ also factor so that

(9) $$\begin{array}{cc} \mathrm{Pr}(|\psi_{w}\rangle ) & =\mathrm{Pr}(|\psi_{x_{0}}\rangle )\mathrm{Pr}(|\psi_{x_{1}}\rangle ||\psi_{x_{0}}\rangle )\\ \; & \;\cdots \mathrm{Pr}(|\psi_{x_{l-1}}\rangle ||\psi_{x_{l-2}}\rangle )\;. \end{array}$$

However, an observer cannot reliably distinguish between different states $|\psi_{x}\rangle$ when measuring a quantum process, and the underlying Markov dynamic is hidden from observation. Thus, the general setting for memoryful quantum processes is that of hidden Markov processes. These are best introduced using concrete models that directly represent a process’s structure.

### 2.4. Presentations of Quantum Processes

A *presentation* for a quantum process is a model with internal states and a transition dynamic between them that emits pure quantum states rather than classical symbols. As for presentations of classical processes, we depict them with state transition diagrams. When $\overset{↔}{X}$ is a Markov or hidden Markov process, $|\Psi_{-\infty :\infty }\rangle =E(\overset{↔}{X})$ can be represented with an extension of Ref. [14]’s classically controlled qubit sources (cCQSs) as follows.

A *hidden Markov chain quantum source* (HMCQS) is a triple (S,Q,T) consisting of

- A finite set $S=\{\sigma_{1},\ldots ,\sigma_{n}\}$ of internal states.
- A finite alphabet $Q=\{|\psi_{0}\rangle ,\ldots ,|\psi_{m-1}\rangle \}$ of pure qudit states, with each $|\psi_{x}\rangle \in H^{d}$.
- A set $T=\{T^{x}:|\psi_{x}\rangle \in Q\}$ of *m* n×n transition matrices. That is, if the source is in state $\sigma_{i}$, with probability $T_{ij}^{x}=\mathrm{Pr}\left(|\psi_{x}\rangle ,\sigma_{j}|\sigma_{i}\right)$, it emits qudit $|\psi_{x}\rangle$ while transitioning to internal state $\sigma_{j}$.

As with HMCs, the stationary distribution for an HMCQS satisfies $\pi =\pi \sum_{x}T^{x}$.

Any HMCQS that exactly reproduces a quantum process is a *generative* HMCQS or generator of the process. Though quantum models cannot be predictive in the same sense as classical models, we can define an analog to classical unifilarity. An HMCQS is *quantum unifilar* if, for every state σ∈S at time *t*, there exists at most a single measurement that determines the internal state at time t+1. This closely parallels the definition of unifilarity in classical stochastic processes. We discuss several implications of quantum unifilarity later.

We call an HMCQS a *classical* controller of a quantum process, since there is nothing quantal about its internal states or transition dynamic. This is in contrast to related classes of quantum models that evolve a finite quantum system according to a quantum operation (defined via a set of Kraus operators) at each timestep. These include *Quantum Markov Chains* (QMCs) [34] and *Hidden Quantum Markov Models* (HQMMs) [35,36,37]. While HMCQSs emit separable quantum states, QMCs and HQMMs generate sequences of measurement outcomes (each corresponding to a particular Kraus operator) that form classical stochastic processes.

Anticipating future effort, we consider it worthwhile to draw out several observations on entanglement between successive qudits at this point. Entanglement means that finite-length qudit sequences are not separable and so are not described by Equation (5). Moreover, their sequence probabilities cannot be straightforwardly defined with reference to an underlying classical stochastic process.

That said, there are systematic ways of defining stationary $|\Psi_{-\infty :\infty }\rangle$ such that the set $\left\{\rho_{0:l}\right\}$ of marginals describes all measurements over blocks of *ℓ* qudits. For example, if the source’s internal structure consists of a *D*-dimensional quantum system interacting unitarily with one qudit per time step, it generates a matrix product state (MPS) with a maximum bond dimension of *D* [38]. If the source operates stochastically (rather than unitarily), then many different MPSs can be emitted with varying probabilities. The collection is then described by *matrix product density operators* (MPDOs) [39]. We refer to these as *entangled qudit processes*. Their dynamical and informational analyses are left for elsewhere. The present goal is to layout the basics for those efforts.

### 2.5. Measured Processes

An agent observing a quantum process has many ways to measure it. Let *M* represent a measurement applied to the qudit in state ρ. In general, *M* is a *positive operator-valued measure* (POVM) described by a set of positive semi-definite Hermitian operators $\left\{E_{y}\right\}$ on the Hilbert space H of dimension *d*. Each $E_{y}$ corresponds to a possible measurement outcome *y*, and POVM elements must sum to the following identity:

$$\begin{array}{c} \sum_{y}E_{y}=I\;. \end{array}$$

*Projection-valued measures* (PVMs) are an important subclass of POVMs with an additional constraint: operators $E_{y}$ must be orthogonal projectors. PVMs have at most *d* elements. A PVM consisting only of rank-one projectors on $H^{d}$ is a *von Neumann measurement* and has exactly *d* elements [5].

A set of measurements applied to a block of *ℓ* qudits is described by some block POVM $M_{0:l}$ with elements $\left\{E_{y_{0:l}}\right\}$ on the Hilbert space $H_{0:l}$ of dimension $d^{l}$. $M_{0:l}$ may include measurements in the joint basis of multiple qudits—measurements essential for fully characterizing entangled processes.

For separable processes we focus on “local” measurements—operators on a single qudit. The measurement operator for a block of *ℓ* qudits then takes a tensor product structure:

(10) $$\begin{array}{c} M_{0:l}=\overset{l-1}{\underset{t=0}{⨂}}M_{t}\;, \end{array}$$

where each $M_{t}$ is a POVM on $H_{t}$.

If we apply the same local POVM *M* to each qudit, then

(11) $$\begin{array}{c} M_{0:l}=\overset{l-1}{\underset{t=0}{⨂}}M\;. \end{array}$$

We refer to this as a *repeated POVM measurement*.

An observer can also have multiple POVMs at their disposal and apply different measurements at different time steps according to some *measurement protocols*. We describe measurement protocols in more detail shortly.

For simplicity, the following ignores $\rho_{0:l}$’s post-measurement state and considers only the measurement outcomes $y_{0:l}$. Thus, we take $M_{0:l}$ to be a stochastic map $\rho_{0:l}\to Y_{0:l}$, with the random variables representing measurement outcomes.

When applying a measurement of the form of Equation (11) to a finite block of *ℓ* qudits, the outcomes factor into a block of *ℓ* classical random variables:

$$\begin{array}{cc} Y_{0:l} & =Y_{0}Y_{1}\cdots Y_{l-1}\\ \; & =M_{0:l}\left(\rho_{0:l}\right)\;, \end{array}$$

where the possible values of each $Y_{t}$ are the POVM measurement outcomes y∈Y. There are ${\left|Y\right|}^{l}$ possible realizations of $Y_{0:l}$. We write a realization (word) of length *ℓ* as $y_{0:l}$.

The probability of any particular measurement outcome for a block of *ℓ* qudits in state $\rho_{0:l}$ is

$$\begin{array}{c} \mathrm{Pr}\left(y_{0:l}\right)=tr\left(E_{y_{0:l}}\rho_{0:l}\right)\;. \end{array}$$

For $\rho_{0:l}$ with the separable form of Equation (6) and identical POVM measurements on each qudit as in Equation (11), we can decompose $E_{y_{0:l}}$ into *ℓ* local operators $E_{y_{t}}$ as follows:

(12) $$\begin{array}{cc} \mathrm{Pr}(y_{0:l}) & =tr\left(E_{y_{0:l}}\sum_{w\in X^{l}}\;\;\mathrm{Pr}\left(w\right)|\psi_{w}\rangle \langle \psi_{w}|\right)\\ \; & =tr\left(\sum_{w\in X^{l}}\;\;\mathrm{Pr}\left(w\right)\prod_{t=0}^{l-1}E_{y_{t}}|\psi_{x_{t}}\rangle \langle \psi_{x_{t}}|\right). \end{array}$$

For a separable qudit process, a sequence $y_{0:l}$ of local measurement outcomes can also be interpreted as the result of sending random variables $X_{0:l}$ from $\overset{↔}{X}$ over the same memoryless noisy channel C:X→Y. C decomposes into the deterministic preparation E and our stochastic measurement M:

$$\begin{array}{c} C=M\circ E\;. \end{array}$$

(Appendix B presents a more thorough description of the classical-quantum channels E and M).

This construction makes it clear that measurement outcomes correspond to classical random variables $Y_{t}$ that take values y∈Y and form a classical process $\overset{↔}{Y}$, with probabilities defined by Equation (12). To express the relationship between a quantum process and a measured classical process, we write

$$\begin{array}{c} \overset{↔}{Y}=M\left(|\Psi_{-\infty :\infty }\rangle \right)\;, \end{array}$$

where M is a repeated, local POVM. If the qudit process is separable, we can also write

$$\begin{array}{c} \overset{↔}{Y}=C\left(\overset{↔}{X}\right)\;. \end{array}$$

### 2.6. Adaptive Measurement Protocols

An observer does not need to repeat the same measurement on every qudit but may apply different POVMs at different time steps according to some algorithm. If the observer uses past measurement outcomes to inform their choice of POVM, we say that they are using an *adaptive measurement protocol*.

The following limits discussion to measurement protocols that have a deterministic finite automata (DFA) as their underlying controller. Similar constructions combining quantum measurement and DFAs have appeared in the context of quantum grammars [40,41,42].

A *deterministic quantum measurement protocol* (DQMP) is defined by the quintuple $\left(S,S_{0},M,Y,\delta \right)$:

- A finite set $S=\{\sigma_{1},\ldots ,\sigma_{n}\}$ of internal states.
- A unique start state $S_{0}\in S$.
- A set of POVMs $M={\left\{M_{\sigma }\right\}}_{\sigma \in S}$, one for each internal state.
- An alphabet Y of *m* symbols corresponding to different measurement outcomes.
- A deterministic transition map δ:S×Y→S.

If S consists of only $S_{0}$, then the DQMP is a repeated POVM measurement for POVM $M_{S_{0}}$. When S has more than one internal state, the POVMs corresponding to different states may have the same or a different number of elements. Likewise, the symbol sets corresponding to their measurement outcomes may be disjoint, or symbols may be repeated.

We can place the following bounds on the size of the set Y: $m\leq \sum_{s}\left|\left\{E_{s,y}\right\}\right|$, where $\left\{E_{s,y}\right\}$ is the set of operators corresponding to POVM $M_{s}$, and $m\geq \max_{s}\left|\left\{E_{s,y}\right\}\right|$ is the size of the POVM with the most elements.

For DQMP M and qudit process $|\Psi_{-\infty :\infty }\rangle$, obtaining a measured process $\overset{↔}{Y}=M\left(|\Psi_{-\infty :\infty }\rangle \right)$ is generically more difficult than for the case of repeated POVM measurements. When an observer begins using protocol M at t=0, they experience two distinct operating regimes: first the *transient* dynamic and then the *recurrent* dynamic. We briefly outline this process and return to the subject when we describe synchronization—a task deeply related to the transient dynamic—in Section 5.

M begins in state $S_{0}$ at t=0. For a given (stationary, ergodic) input $|\Psi_{-\infty :\infty }\rangle$, as t→∞, the DQMP approaches a stationary distribution over a subset of its internal states $\pi =\{\mathrm{Pr}\left(\sigma_{i}\right)>0,\;\mathrm{for}\;\mathrm{all}\;\sigma_{i}\in S_{r}\}$, where $S_{r}\subseteq S$ is the set of recurrent states. This distribution (and even which states are in $S_{r}$) depends on $|\Psi_{-\infty :\infty }\rangle$. The recurrent dynamic is determined by this stationary distribution and the transition probabilities between states in $S_{r}$. Any state not in $S_{r}$ is in the transient state set $S_{t}$.

The transient dynamic describes how M goes from $S_{0}$ at t=0 to its recurrent dynamic over $S_{r}$, which may occur at a finite time $t=t_{sync}$ or only asymptotically as t→∞. In general, two dynamics produce two distinct measured processes: ${\overset{↔}{Y}}_{r}$, which is stationary and ergodic by construction, and $Y_{0:t_{sync}}$, which is not. The final measured process has two components—i.e., $\overset{↔}{Y}=\left\{{\overset{↔}{Y}}_{r},Y_{0:t_{sync}}\right\}$.

### 2.7. Discussion

These nested layers of complication suggest working through a concrete example and restating the overall goals.

Imagine that an observer measures a single qubit from a quantum source that emitted state $\rho_{t}$ using a projective measurement in the computational basis $M_{01}=\left\{|0\rangle \langle 0|,|1\rangle \langle 1|\right\}$. The possible measurement outcomes $y_{0}=0$ and $y_{0}=1$ occur with the following probabilities:

$$\begin{array}{cc} \mathrm{Pr}(y_{0}=0) & =tr(|0\rangle \langle 0|\rho_{t})\\ \mathrm{Pr}(y_{0}=1) & =tr(|1\rangle \langle 1|\rho_{t})\;, \end{array}$$

respectively. These two values determine the distribution for the random variable $Y_{0}$ and, by applying the same projective measurement to $\rho_{0:l}$, we completely determine the statistics $\mathrm{Pr}(Y_{0:l})$ of the measured block. Continuing this procedure for l→∞ defines the measured process $\overset{↔}{Y}$.

Naturally, the observer can also choose to apply measurements in another basis, e.g., $M_{\pm }=\left\{|+\rangle \langle +|,|-\rangle \langle -|\right\}$, where $|\pm \rangle =\frac{1}{\sqrt{2}}\left(|0\rangle \pm |1\rangle \right)$. This typically results in a measured process ${\overset{↔}{Y}}^{\prime }$ with radically different statistical features.

Finally, an observer could use an adaptive measurement protocol M. It starts in state $s=S_{0}$ and measures with $M_{01}$. If $y_{0}=0$, it stays in $S_{0}$ and continues using $M_{01}$. If $y_{0}=1$, it transitions to a new internal state and uses $M_{\pm }$ on the next qubit. Regardless of the outcome of $M_{\pm }$, it returns to $S_{0}$ and measures the next qubit with $M_{01}$. The measured process ${\overset{↔}{Y}}^{\prime \prime }$ will be distinct from both $\overset{↔}{Y}$ and ${\overset{↔}{Y}}^{\prime }$ and may consist of both a transient and recurrent component.

With this setting laid out, we can now more precisely state the questions the following development answers:

1. Given the density matrices $\rho_{0:l}$ describing sequences of *ℓ* separable qudits, what are the general properties of sequences $Y_{0:l}$ of measurement outcomes? This is Section 3’s focus. There, $\rho_{0:l}$’s quantum information properties bound the classical information properties of measurement sequences $Y_{0:l}$ for certain classes of measurements.
2. Given a hidden Markov chain quantum source, when is an observer with knowledge of the source able to determine the internal state (synchronize)? Can the observer remain synchronized at later times? Section 5 addresses this.
3. If an observer encounters an unknown qudit source, how accurately can the observer estimate the informational properties of the emitted process through tomography with limited resources? How can they build approximate models of the source if they reconstruct $\rho_{0:l}$ for some finite *ℓ*? This is Section 6’s subject.

Additionally, Section 4 illustrates these general results and the required analysis methods using specific examples of qudit processes.

## 3. Information in Quantum Processes

We wish to develop an information-theoretic analysis of quantum processes for which the observed sequences depend on the observer’s choice of measurement. (Much of this parallels the classical information measures reviewed in Appendix A). This requires a more general approach using density matrices $\rho_{0:l}$ that contain all the information necessary to describe the outcome of any measurement performed on *ℓ*-qudit blocks. We use quantum information theory to study properties of the set of $\rho_{0:l}$ and then relate them to classical properties of measurement sequences described in Appendix A. We begin by briefly reviewing several basic quantities in quantum information theory. References [6,33] give a more complete picture of the subject.

### 3.1. von Neumann Entropy

In quantum information theory, the von Neumann entropy plays a role similar to that of the Shannon entropy in classical information theory. Given a mixed quantum state ρ, the *von Neumann entropy* is

(13) $$\begin{array}{cc} S(\rho ) & =-tr(\rho \log_{2}\rho )\\ \; & =-\sum_{i}\lambda_{i}\log_{2}\lambda_{i}\;, \end{array}$$

where the $\lambda_{i}$s are the eigenvalues of the density matrix ρ. S(ρ)=0 if and only if ρ is a pure state. We use $\log_{2}\left(\cdot \right)$; therefore, the units of the von Neumann entropy will be *bits*.

From Equation (13), the von Neumann entropy is the Shannon entropy of the eigenvalue distribution of density matrix ρ. Therefore,

(14) $$S\left(\rho \right)=\min_{M}H\left[M(\rho )\right],$$

where H[·] is the Shannon entropy, and the minimum is taken over the set of all rank-one POVMs. The minimum will always be a PVM with projectors that compose ρ’s eigenbasis [6]. We use brackets to indicate that M(ρ) is a classical probability distribution over the measurement outcomes.

To monitor correlations between two quantum systems, we use the quantum relative entropy:

(15) $$\begin{array}{c} S\left(\rho ∥\sigma \right)\equiv tr\left(\rho \log_{2}\rho \right)-tr\left(\rho \log_{2}\sigma \right)\;, \end{array}$$

where ρ and σ are the density operators of the two systems. The quantum relative entropy is non-negative and defined as follows:

(16) $$\begin{array}{c} S(\rho ∥\sigma )\geq 0\;, \end{array}$$

with equality if and only if ρ=σ, a result known as *Klein’s Inequality* [33].

The *joint quantum entropy* for a state $\rho^{AB}$ of a bipartite system AB is

$$\begin{array}{cc} S(A,B) & =S(\rho^{AB})\\ \; & =-tr(\rho^{AB}\log_{2}\rho^{AB})\;, \end{array}$$

We can further define a *conditional quantum entropy* of system *A* conditioned on system *B* as

(17) $$\begin{array}{c} S(A|B)=S(A,B)-S(B)\;, \end{array}$$

where $S\left(B\right)=S\left(\rho^{B}\right)$. Note that S(A|B)≠S(B|A).

In contrast to the classical case, the conditional quantum entropy may be negative—a phenomenon leveraged in super-dense coding protocols [6]. Equivalently, the conditional quantum entropy can be written using the quantum relative entropy as

(18) $$\begin{array}{cc} S(A|B) & =-S(\rho^{AB}∥I^{A}⊗\rho^{B})\\ \; & =\log_{2}\left(d_{A}\right)-S\left(\rho^{AB}∥\frac{I^{A}}{d_{A}}⊗\rho^{B}\right)\;, \end{array}$$

where $I^{A}$ is the identity operator on Hilbert space $H^{A}$ with dimension $d_{A}$.

The *quantum mutual information* between quantum subsystems *A* and *B* is given by

(19) $$\begin{array}{cc} S(A:B) & =S(A)-S(A|B)\\ \; & =S(A)+S(B)-S(A,B)\;. \end{array}$$

The quantum mutual information is symmetric and non-negative. If the joint system AB is in a pure state, then S(A,B) will be zero, and S(A)=S(B). It can also be expressed as a quantum relative entropy as

(20) $$\begin{array}{cc} S(A:B) & =S\left(\rho^{AB}∥\rho^{A}⊗\rho^{B}\right)\;. \end{array}$$

A few additional well-known properties of the von Neumann entropy facilitate later results. First, each $\rho_{0:l}$ for separable qudit sequences is a finite mixture of states formed from length-*ℓ* words of an underlying classical process, so the following will be useful.

**Lemma** **1.** *Consider a random variable X that takes values x∈{0,1,…,n} with corresponding probabilities $\left\{p_{0},p_{1},\ldots p_{n}\right\}$. Given a set of density matrices $\left\{\rho_{0},\rho_{1},\ldots ,\rho_{n}\right\}$, the following inequality holds [33]:*

$$\begin{array}{c} S\left(\sum_{x=0}^{n}p_{x}\rho_{x}\right)\leq H\left[X\right]+\sum_{x=0}^{n}p_{x}S\left(\rho_{x}\right)\;, \end{array}$$

*with equality if and only if the all $\rho_{x}$ have support on orthogonal subspaces.*

Second, since we use quantum channels to both prepare and measure a qudit process, we make use of the fact that the quantum relative entropy is monotonic [6]:

(21) $$\begin{array}{c} S(\rho ∥\sigma )\geq S(E(\rho )∥E(\sigma ))\;, \end{array}$$

where E is any quantum channel. This inequality becomes an equality if and only if there exists a recovery map R such that R(E(ρ))=ρ and R(E(σ))=σ [43].

### 3.2. Quantum Block Entropy

Since stationary qudit processes are correlated across time, we explore how the von Neumann entropy for qudit blocks scales with block size. The following gives bounds on the possible measurement sequences one can observe from a quantum information source. As the von Neumann entropy generalizes the Shannon entropy, the results here (and many of the proofs) are natural generalizations of those in Appendix A. We also note that exactly determining $\rho_{0:l}$ becomes practically infeasible for large *ℓ*. And so, Section 6 addresses how to approximate properties and models for qudit processes when restricted to measurements of finite length blocks.

For a qudit process, we define the quantum block entropy as the von Neumann entropy of a block of *ℓ* consecutive qudits:

$$\begin{array}{c} S\left(l\right)\equiv -tr\left(\rho_{0:l}\log_{2}\rho_{0:l}\right)\;. \end{array}$$

If $\rho_{0:l}$ is a pure state, S(l)=0. By the same logic as the classical case, S(0)≡0.

Many properties of the classical block entropy hold for S(l).

**Proposition** **1.** *For a stationary qudit process, S(l) is a nondecreasing function of ℓ.*

**Proof.**  As a consequence of the strong subadditivity of the von Neumann entropy [33],

(22) $$\begin{array}{c} S\left(\rho^{A}\right)+S\left(\rho^{C}\right)\leq S\left(\rho^{AB}\right)+S\left(\rho^{BC}\right)\;. \end{array}$$

Let $\rho^{ABC}=\rho_{0:2l+1}$, where $\rho^{A}=\rho_{0:l}$, $\rho^{B}=\rho_{l}$, and $\rho^{C}=\rho_{l+1:2l+1}$.

Incorporating qudit process stationarity, we rewrite Equation (22) as follows:

(23) $$\begin{array}{cc} S\left(\rho_{0:l}\right)+S\left(\rho_{l+1:2l+1}\right) & \leq S\left(\rho_{0:l+1}\right)+S\left(\rho_{l:2l+1}\right)\\ S(l)+S(l) & \leq S(l+1)+S(l+1)\\ S(l) & \leq S(l+1)\;, \end{array}$$

where Equation (23) follows from stationarity.

Thus, S(l)≤S(l+1), for all l≥0, and S(l) is a nondecreasing function of *ℓ*. □

**Proposition** **2.** *For a stationary qudit process, S(l) is concave.*

**Proof.**  The von Neumann entropy is strongly subadditive [33], meaning that

(24) $$\begin{array}{c} S\left(\rho^{ABC}\right)+S\left(\rho^{B}\right)\leq S\left(\rho^{AB}\right)+S\left(\rho^{BC}\right)\;. \end{array}$$

For l≥3, let $\rho^{ABC}=\rho_{0:l}$, where $\rho^{A}=\rho_{0}$, $\rho^{B}=\rho_{1:l-1}$, and $\rho^{C}=\rho_{l-1}$

We can rewrite Equation (24) by incorporating the stationarity of qudit processes:

(25) $$\begin{array}{cc} S\left(\rho_{0:l}\right)+S\left(\rho_{1:l-1}\right) & \leq S\left(\rho_{0:l-1}\right)+S\left(\rho_{1:l}\right)\\ S(l)+S(l-2) & \leq S(l-1)+S(l-1)\\ S(l)-2S(l-1)+S(l-2) & \leq 0\;, \end{array}$$

where Equation (25) follows from stationarity.

Thus, S(l) is concave. □

For a separable qudit process formed by passing a classical process through a classical-quantum channel, its block entropies are related in the following way:

**Proposition** **3.** *Let $|\Psi_{-\infty :\infty }\rangle =E(\overset{↔}{X})$. The block entropies of $|\Psi_{-\infty :\infty }\rangle$ and $\overset{↔}{X}$ obey*

$$\begin{array}{c} S\left(\rho_{0:l}\right)\leq H\left[X_{0:l}\right]\;, \end{array}$$

*for all ℓ, with equality if and only if Q consists of |X| orthogonal pure states in H of dimension d≥|X|.*

**Proof.**  Recall from Equation (6) that for separable qudit processes,

$$\begin{array}{c} \rho_{0:l}=\sum_{w\in X^{l}}\mathrm{Pr}\left(w\right)|\psi_{w}\rangle \langle \psi_{w}|\;, \end{array}$$

with each $|\psi_{w}\rangle$ taking the separable form of Equation (5) and $w=x_{0:l}$.

We first note that, for all symbols in X to be associated with orthogonal qudit states, the minimum dimension of the Hilbert space is $d_{min}=\left|X\right|$.

With $\rho_{0:l}$ written as a mixture of separable qudit words, we apply Lemma 1 to obtain

$$\begin{array}{cc} S(\rho_{0:l}) & \leq H\left[X_{0:l}\right]+\sum_{w\in X^{l}}\mathrm{Pr}\left(w\right)S\left(|\psi_{w}\rangle \right)\\ \; & \leq H\left[X_{0:l}\right]\;, \end{array}$$

where $w=x_{0:l}$. The second term evaluates to zero in the final line, since each $|\psi_{w}\rangle$ is a pure state. That is, $S(|\psi_{w}\rangle )=0$ for all $w\in X^{l}$.

Equality occurs if and only if the states $|\psi_{w}\rangle$ have support on orthogonal subspaces, which requires that d≥|X| and all elements of Q are orthogonal. □

We cannot use $S(\rho_{0:l})$ to bound the block entropy of a measured process $\overset{↔}{Y}$ for *general* POVM measurements. For the case where the measurement $M_{0:l}$ consists only of rank-one POVMs (including all PVMs) however, the following holds:

(26) $$\begin{array}{c} S\left(\rho_{0:l}\right)\leq H\left[M_{0:l}\left(\rho_{0:l}\right)\right]\;, \end{array}$$

with equality if and only if the measurement is performed in the minimum-entropy (eigen)basis of $\rho_{0:l}$. This follows directly from Equation (14).

**Proposition** **4.** *Let $\overset{↔}{Y}=M\left(|\Psi_{-\infty :\infty }\rangle \right)$, where M is a repeated rank-one POVM measurement. The block entropies of $|\Psi_{-\infty :\infty }\rangle$ and $\overset{↔}{Y}$ then obey*

$$\begin{array}{c} S\left(\rho_{0:l}\right)\leq H\left[Y_{0:l}\right]\;, \end{array}$$

*for all ℓ, with equality if and only if $|\Psi_{-\infty :\infty }\rangle$ is a separable process with an orthogonal alphabet Q and M uses a POVM whose operators include one projector for each element in Q.*

**Proof.**  The bound follows directly from Equation (26) because repeated rank-one POVM measurements are a subclass of the more general measurement sequence $M_{0:l}$.

The condition for equality also follows from Equation (26) but requires more justification. First, we consider measuring the single-qubit marginal $\rho_{0}$ with POVM *M*. For equality, each element of the eigenbasis of $\rho_{0}$ ($\left\{|e_{i}\rangle \right\}$) must have a corresponding operator in *M* that is a projector on that eigenspace ($E_{i}=|e_{i}\rangle \langle e_{i}|$).

We can write $\rho_{0}=\sum_{i}p_{i}|e_{i}\rangle \langle e_{i}|$. Since we apply the same POVM to each qudit, all blocks of length *ℓ* must have eigenstates of the form ${⨂}_{t=0}^{l-1}|e_{t}\rangle$—i.e., they must take the separable form of Equation (5)—making $|\Psi_{-\infty :\infty }\rangle$ a separable process with a quantum alphabet Q of orthogonal states. The *M* consisting of projectors onto the states in Q is then the minimum-entropy measurement over blocks $\rho_{0:l}$. Note that there may be other elements of the POVM that are not projectors if the probability of those measurement outcomes is 0 when applied to $\rho_{0:l}$. (If the process does not make use of that part of Hilbert space, it does not matter how it is measured). □

To summarize, in the case of separable qudit processes, S(l) is upper-bounded by the underlying classical process’s block entropy $H\left[X_{0:l}\right]$. For repeated measurement with rank-one POVMs, S(l) serves as a lower bound on the block entropy of all classical measured processes $H\left[Y_{0:l}\right]$. There is no direct relationship between $H\left[X_{0:l}\right]$ and $H\left[Y_{0:l}\right]$. Rather, it depends on the specifics of E and M.

### 3.3. von Neumann Entropy Rate

The von Neumann entropy rate of a qudit process is

(27) $$\begin{array}{c} s=\lim_{l\to \infty }\frac{S(l)}{l}\;. \end{array}$$

The units of *s* are *bits per time step*. This quantity is equivalent to the *mean entropy*, first introduced in the context of quantum statistical mechanics [44]. The limit exists for all stationary processes [45]. Operationally, *s* is the optimal coding rate for a stationary quantum source [17].

**Proposition** **5.** *For a stationary qudit process, we can equivalently write the von Neumann entropy rate as*

(28) $$\begin{array}{c} s=\lim_{l\to \infty }S\left(\rho_{0}|\rho_{-l:0}\right)\;. \end{array}$$

**Proof.**  This proof closely follows the proof for the classical entropy rate in Ref. [46]. We begin by showing that $\lim_{l\to \infty }S\left(\rho_{0}|\rho_{-l:0}\right)$ exists and then that it is equivalent to the limit in Equation (27). The limit exists if $S(\rho_{0}|\rho_{-l:0})$ is a decreasing, non-negative function of *ℓ*:

$$\begin{array}{cc} S(\rho_{0}|\rho_{-l:0}) & =S\left(\rho_{-l:1}\right)-S\left(\rho_{-l:0}\right) \end{array}$$

(29) $$\begin{array}{cc} \; & =S(l+1)-S(l) \end{array}$$

(30) $$\begin{array}{cc} \; & \geq 0\;, \end{array}$$

where Equation (29) follows from stationarity, and Equation (30) follows from the nondecreasing nature of S(l). This, combined with the fact that S(l) is concave, means that $\lim_{l\to \infty }S\left(\rho_{0}|\rho_{-l:0}\right)$ exists.

Now, we establish that $\lim_{l\to \infty }S\left(\rho_{0}|\rho_{-l:0}\right)=\lim_{l\to \infty }S\left(l\right)/l$. Through repeated application of Equation (17) to a block of length *ℓ*, we obtain the following chain rule for the von Neumann entropy:

(31) $$\begin{array}{c} S\left(\rho_{0:l}\right)=\sum_{j=0}^{l-1}S\left(\rho_{j}|\rho_{j-1}\cdots \rho_{0}\right)\;. \end{array}$$

We can modify indices (due to stationarity) and divide both sides by *ℓ* to obtain

(32) $$\begin{array}{c} \frac{S(\rho_{0:l})}{l}=\frac{1}{l}\sum_{i=1}^{l}S\left(\rho_{i}|\rho_{i-1}\cdots \rho_{1}\right)\;. \end{array}$$

The final steps require the following result, known as the Cesáro mean [46].

**Lemma** **2.** *If $a_{n}\to a$ and $b_{n}=\frac{1}{n}\sum_{i=1}^{n}a_{i}$, then $b_{n}\to a$.*

Taking the limit of both sides of Equation (32) and applying Lemma 2, we find

(33) $$\begin{array}{c} \lim_{l\to \infty }\frac{S(\rho_{0:l})}{l}=\lim_{l\to \infty }S\left(\rho_{l}|\rho_{l-1}\cdots \rho_{1}\right)\;. \end{array}$$

Using stationarity, $\lim_{l\to \infty }S\left(\rho_{l}|\rho_{l-1}\cdots \rho_{1}\right)=\lim_{l\to \infty }S\left(\rho_{0}|\rho_{-l:0}\right)$. When combined with Equation (33), this proves that our two definitions of *s* are equivalent. □

**Figure 2:**
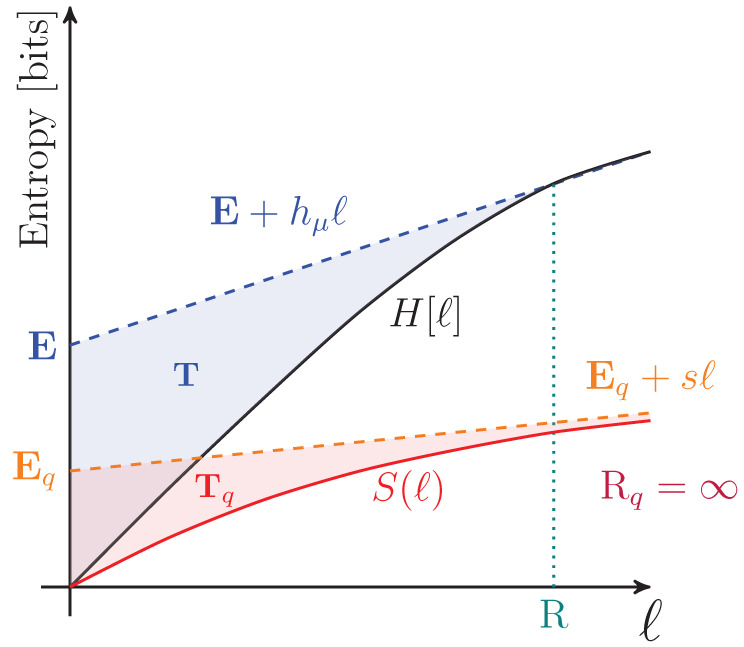
Convergence of the block entropies to their linear asymptotes. H[l] is the block entropy for a finitary classical process with Markov order *R* (see Appendix A), and S(l) is the quantum block entropy for a finitary quantum process with infinite quantum Markov order $R_{q}$. For the classical process, E is the excess entropy, and $h_{\mu }$ is its Shannon entropy rate. Similarly, for a quantum process, $E_{q}$ is the quantum excess entropy, and *s* is its von Neumann entropy rate. The area of the red shaded region is the quantum transient information $T_{q}$.

To motivate a number of the following results, it is important to appreciate that simply because a process has a von Neumann entropy rate given by Equation (27) does not imply that an observer is able to perform a measurement on any qudit such that the uncertainty in that individual measurement is *s*. Rather, obtaining *s* corresponds to the measurement basis over the entire chain of qudits for which the distribution of outcomes has the minimal Shannon entropy. This basis is highly nonlocal or otherwise experimentally infeasible for many nontrivial examples. As in the classical case, *s* appears graphically as the slope of S(l) as l→∞, as shown in Figure 2.

For separable qudit processes, we can also relate *s* to the classical entropy rate of the underlying process $\overset{↔}{X}$ as follows.

**Proposition** **6.** *Let $|\Psi_{-\infty :\infty }\rangle =E(\overset{↔}{X})$. The von Neumann entropy rate s of $|\Psi_{-\infty :\infty }\rangle$ then obeys the bound*

$$\begin{array}{c} s\leq h_{\mu }^{X}\;, \end{array}$$

*where $h_{\mu }^{X}$ is the Shannon entropy rate of $\overset{↔}{X}$. Equality occurs if and only if Q consists of |X| orthogonal pure states in $H^{d}$ of dimension d≥|X|.*

**Proof.**  Divide both sides of Proposition 3 by *ℓ* and take the limit of both sides as l→∞ to obtain

$$\begin{array}{c} \lim_{l\to \infty }\frac{S(\rho_{0:l})}{l}\leq \lim_{l\to \infty }\frac{H[X_{0:l}]}{l}\;. \end{array}$$

From Equation (27), the left side is *s*, and from Equation (A1), the right side is $h_{\mu }^{X}$. The condition for equality is inherited from Proposition 3, concluding the proof. □

Restricting once again to repeated measurements with rank-one POVMs, we can prove the following bound for measured processes.

**Proposition** **7.** *Let $\overset{↔}{Y}=M\left(|\Psi_{-\infty :\infty }\rangle \right)$, and let M be a repeated rank-one POVM. The measured entropy rate $h_{\mu }^{Y}$ then obeys*

$$\begin{array}{c} s\leq h_{\mu }^{Y}\;, \end{array}$$

*where s is the von Neumann entropy rate of $|\Psi_{-\infty :\infty }\rangle$, with equality if and only if $|\Psi_{-\infty :\infty }\rangle$ is a separable process with an orthogonal alphabet Q and M uses a POVM whose operators include a projector for each element in Q.*

**Proof.**  Divide both sides of Proposition 4 by *ℓ* and take the limit of both sides as l→∞ to obtain

$$\begin{array}{c} \lim_{l\to \infty }\frac{S(\rho_{0:l})}{l}\leq \lim_{l\to \infty }\frac{H[Y_{0:l}]}{l}\;. \end{array}$$

The left side is *s*, and the right side $h_{\mu }^{Y}$. The conditions for equality are inherited from Proposition 4, concluding the proof. □

### 3.4. Quantum Redundancy

Unlike a classical process, the maximum entropy rate for a qudit process depends on the size of the Hilbert space rather than on the size of the alphabet Q. For Hilbert space of dimension *d*, the largest possible value of *s* is $\log_{2}\left(d\right)$, corresponding to an i.i.d. sequence of qudits, each in a maximally mixed state $\rho_{iid}=I/d$.

A qudit process can be compressed down to its von Neumann entropy rate *s*. The amount that it can be compressed is the *quantum redundancy*:

(34) $$\begin{array}{c} R_{q}\equiv \log_{2}\left(d\right)-s\;. \end{array}$$

Statistical biases in individual qudits and temporal correlations between them offer opportunities for compression. $R_{q}$ includes the effects of both.

For separable qudit processes, we can bound the quantum redundancy using properties of the underlying classical process:

**Proposition** **8.** *Let $\overset{↔}{X}$ be a classical process with redundancy $R^{X}$, symbol alphabet X, and entropy rate $h_{\mu }^{X}$, and let $|\Psi_{-\infty :\infty }\rangle =E(\overset{↔}{X})$ be a qudit process with redundancy $R_{q}$, Hilbert space of dimension d, and entropy rate s.*

*For d≥|X|,*

$$\begin{array}{c} R_{q}\geq R^{X}\;, \end{array}$$

*with equality if and only if d=|X| and Q consist of |X| orthogonal pure states.*

*For d<|X|,*

$$\begin{array}{c} R_{q}<R^{X}+\left(h_{\mu }^{X}-s\right)\;, \end{array}$$

*where the term $\left(h_{\mu }^{X}-s\right)$ is always positive, as indicated by Proposition (6).*

**Proof.**  First, consider d=|X|:

$$\begin{array}{cc} R_{q} & =\log_{2}\left(|X|\right)-s\\ \; & =R^{X}+h_{\mu }^{X}-s\\ \; & \geq R^{X}\;, \end{array}$$

The final line comes from Proposition 6, as does the condition for equality.

For d>|X|,

$$\begin{array}{cc} R_{q} & =\log_{2}\left(d\right)-s\\ \; & >\log_{2}\left(|X|\right)-s\\ \; & >R^{X}\;. \end{array}$$

There is no opportunity for equality. In this case, Q will not span H, naturally leading to more redundancy.

Finally, for d<|X|,

$$\begin{array}{cc} R_{q} & <\log_{2}\left(d\right)-s\\ \; & <\log_{2}\left(|X|\right)-s\\ \; & <R^{X}+\left(h_{\mu }^{X}-s\right)\;, \end{array}$$

concluding the proof. □

We can also compare the classical redundancy of a measured process (obtained through repeated use of a rank-one POVM) to the quantum redundancy of the qudit process being measured.

**Proposition** **9.** *Let $\overset{↔}{Y}$ be a measured process such that $\overset{↔}{Y}=M\left(|\Psi_{-\infty :\infty }\rangle \right)$ and M be a repeated rank-one POVM. Let $\overset{↔}{Y}$ have redundancy $R^{Y}$, and let $|\Psi_{-\infty :\infty }\rangle$ have quantum redundancy $R_{q}$. Then,*

(35) $$\begin{array}{c} R_{q}\leq R^{Y}\;, \end{array}$$

*with equality if and only if d=|Y|, $|\Psi_{-\infty :\infty }\rangle$ is a separable process with an orthogonal alphabet Q and M uses a POVM whose operators include a projector for each element in Q.*

**Proof.**  A rank-one POVM on H must have at least *d* elements; therefore, for |Y|≥d,

$$\begin{array}{cc} R_{q} & =\log_{2}\left(d\right)-s\\ \; & \leq \log_{2}\left(|Y|\right)-s\\ \; & \leq R^{Y}+h_{\mu }^{Y}-s\\ \; & \leq R^{Y}\;. \end{array}$$

Going from the first line to the second provides a condition for equality: d=|Y|. Proposition 7 is used in the final line and provides the other conditions for equality. □

### 3.5. Quantum Entropy Gain

We can take discrete-time derivatives of S(l), as was done for H[l] in [11]. This process is summarized in Appendix A. We call the first derivative of S(l) the *quantum entropy gain* as

(36) $$\begin{array}{c} \Delta S(l)\equiv S(l)-S(l-1)\;, \end{array}$$

for l>0. The units for the quantum entropy gain are *bits per time step*, and we set the boundary condition at length l=0 to $\Delta S\left(0\right)=\log_{2}\left(d\right)$, where *d* is the Hilbert space dimension. Since S(l) is monotonically increasing, ΔS(l)≥0.

The quantum entropy gain is the amount of additional uncertainty introduced by including the $l^{\mathrm{th}}$ qudit in a block, where that uncertainty is quantified by the von Neumann entropy.

By combining Equations (17) and (36), we can write ΔS(l) as

$$\begin{array}{c} \Delta S\left(l\right)=S\left(\rho_{0}|\rho_{-l:0}\right)\;. \end{array}$$

This allows for relating the quantum entropy gain and the von Neumann entropy rate as follows:

(37) $$\begin{array}{c} s=\lim_{l\to \infty }\Delta S\left(l\right)\;. \end{array}$$

Thus, paralleling the classical case, the quantum entropy gain serves as a finite-*ℓ* approximation of the von Neumann entropy rate:

(38) $$\begin{array}{cc} s(l) & \equiv \Delta S(l)\;. \end{array}$$

s(l) serves as the best estimate for the entropy rate of a qudit process that can be made by an observer who only has access to measurement statistics for length-*ℓ* blocks of qudits.

**Figure 3:**
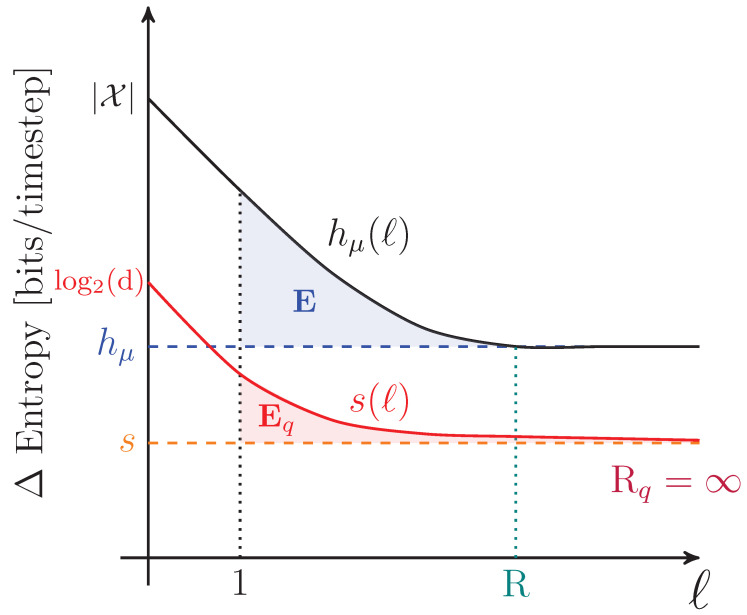
Convergence of ΔH(l) (for a finitary classical process with Markov order *R*) and ΔS(l) (for a finitary quantum process with infinite quantum Markov order $R_{q}$) to the processes’ entropy rates, $h_{\mu }$ and *s*. The shaded areas are the classical (blue) and quantum (red) excess entropies.

The way in which the entropy rate estimate converges and its relationship to other information properties of a qudit process are summarized in Figure 3.

### 3.6. Quantum Predictability Gain

**Figure 4:**
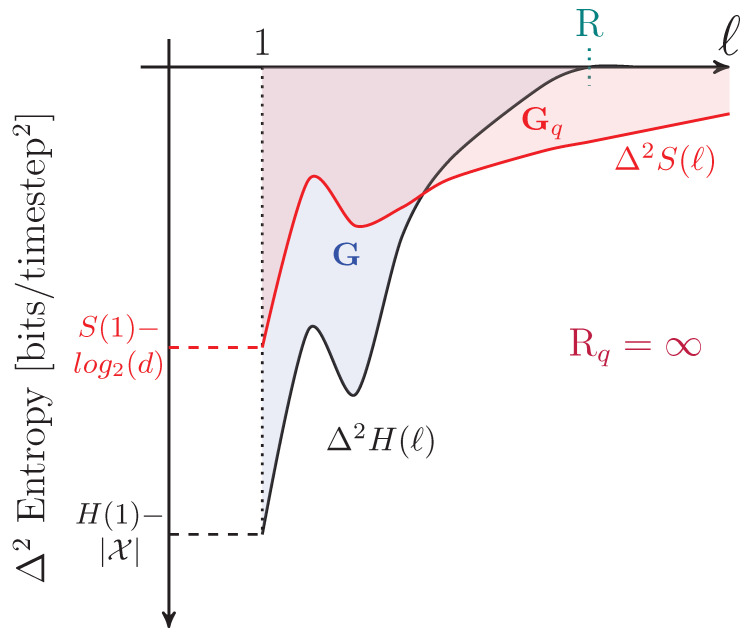
Convergence of the predictability ($\Delta^{2}H\left(l\right)$ and $\Delta^{2}S\left(l\right)$) to 0 for a finitary classical process with Markov order *R* and a finitary quantum process with infinite quantum Markov order $R_{q}$. Recall Section III, which notes that changes in the magnitude of these second-order quantities—rates of change in a rate—indicate increasing or decreasing unpredictability. Said differently, their nonmonotonities reflect that both entropy rates capture length-dependent correlations. Note that the predictability is not monotonic (unlike the block entropy and its first derivative). The overlapping shaded areas represent the magnitude of the classical (blue) and quantum (red) predictabilities, G and $G_{q}$, which are negative by convention.

We call the second derivative of S(l) the *quantum predictability gain*, which is given by

$$\begin{array}{cc} \Delta^{2}S\left(l\right) & \equiv \Delta s(l)\\ \; & =s(l)-s(l-1)\;, \end{array}$$

for l>0. The units of $\Delta^{2}S\left(l\right)$ are *bits per time step^2^*. Since S(l) is concave, then $\Delta^{2}S\left(l\right)\leq 0$. $|\Delta^{2}S\left(l\right)|$ quantifies how much an observer’s estimate of the von Neumann entropy rate *s* improves if they enlarge their observations from blocks of l−1 to blocks of *ℓ* qudits. The generic convergence behavior of $\Delta^{2}S\left(l\right)$ is shown in Figure 4.

For all higher-order discrete derivatives of S(l) (as with the classical block entropy),

$$\begin{array}{c} \lim_{l\to \infty }\Delta^{n}S\left(l\right)=0,n\geq 2\;. \end{array}$$

This follows directly from the existence of the limit in Equation (27) for stationary quantum states.

### 3.7. Total Quantum Predictability

Up to this point, introducing new information-theoretic characteristics of separable quantum processes proceeded by taking discrete-time derivatives of the von Neumann block entropy. We can likewise integrate the functions $\Delta^{n}S\left(l\right)$, as is done for the classical case with Equation (A3). While this starts off straightforwardly, a number of interesting new informational quantities emerge.

These properties of qudit processes take the following general form:

$$\begin{array}{c} I_{n}^{q}\equiv \sum_{l=l_{0}}^{\infty }\left[\Delta^{n}S\left(l\right)-\lim_{l\to \infty }\Delta^{n}S\left(l\right)\right]\;, \end{array}$$

where $l_{0}$ is the first value of *ℓ* for which $\Delta^{n}S\left(l\right)$ is defined.

$I_{2}^{q}$, the first of these, monitors the convergence of the quantum predictability gain $\Delta^{2}S\left(l\right)$ to its limit of 0 for l→∞. We use $l_{0}=1$ to get the *total quantum predictability* $G_{q}$:

(39) $$\begin{array}{cc} G_{q} & \equiv I_{2}^{q}\\ \; & =\sum_{l=1}^{\infty }\Delta^{2}S\left(l\right)\;. \end{array}$$

The units of $G_{q}$ are *bits per time step*. Note that $G_{q}\leq 0$ because $\Delta^{2}S\left(l\right)\leq 0$ for all *ℓ*.

$G_{q}$ can be interpreted by relating it to a previously established property of qudit processes: quantum redundancy.

**Proposition** **10.** *For a stationary qudit process,*

$$\begin{array}{c} G_{q}=-R_{q}\;. \end{array}$$

**Proof.**  Applying Equation (A3) to Equation (39), we find that

$$\begin{array}{cc} G_{q} & =\lim_{l\to \infty }\Delta S\left(l\right)-\Delta S\left(0\right)\\ \; & =s-\log_{2}\left(d\right)\\ \; & =-R_{q}\;. \end{array}$$

Here, the second line follows from Equation (37) and the third from Equation (34). □

Thus, $|G_{q}|$ is the total amount of predictable information per time step for a qudit process.

Rather immediately, one sees that the amount of information in an individual qudit decomposes into

$$\begin{array}{c} \log_{2}\left(d\right)=\left|G_{q}\right|+s\;. \end{array}$$

$|G_{q}|$ is the amount of quantum information within a qudit that is predictable, whereas *s* is the amount of information that is irreducibly random.

The relation $|G_{q}|=R_{q}$ can be combined with Propositions 8 and 9 to prove two corollaries.

**Corollary** **1.** *Let $\overset{↔}{X}$ be a classical process with total predictability $G^{X}$, symbol alphabet X, and entropy rate $h_{\mu }^{X}$, and let $|\Psi_{-\infty :\infty }\rangle =E(\overset{↔}{X})$ be a qudit process with total quantum predictability $G_{q}$, Hilbert space of dimension d, and entropy rate s.*

*For d≥|X|,*

$$\begin{array}{c} |G_{q}\left|\geq \right|G^{X}|\;, \end{array}$$

*with equality if and only if d=|X| and Q consist of |X| orthogonal pure states.*

*For d<|X|,*

$$\begin{array}{c} |G_{q}\left|<\right|G^{X}|+\left(h_{\mu }^{X}-s\right)\;, \end{array}$$

*where the term $\left(h_{\mu }^{X}-s\right)$ is always positive, as derived from Proposition (6).*

**Proof.**  This follows immediately from combining Propositions 8 and 10. □

**Corollary** **2.** *Let $\overset{↔}{Y}$ be a measured process such that $\overset{↔}{Y}=M\left(|\Psi_{-\infty :\infty }\rangle \right)$, and M is a repeated rank-one POVM. Let $\overset{↔}{Y}$ have redundancy $G^{Y}$, and let $|\Psi_{-\infty :\infty }\rangle$ have quantum redundancy $G_{q}$. Then,*

$$\begin{array}{c} |G_{q}\left|\leq \right|G^{Y}|\;, \end{array}$$

*with equality if and only if d=|Y|, $|\Psi_{-\infty :\infty }\rangle$ is a separable process with an orthogonal alphabet Q and M uses a POVM whose operators include a projector for each element in Q.*

**Proof.**  This follows immediately from combining Propositions 9 and 10. □

Graphically, the total quantum predictability is the area between the predictability gain curve and its linear asymptote of 0, as seen in Figure 4. The von Neumann entropy rate and total predictability lend insight into compression limits for stationary sources. They do not indicate, however, whether that compression is achievable due to bias within individual qudit states or correlations between qudits. For that, we must continue our way back up the entropy hierarchy.

### 3.8. Quantum Excess Entropy

The convergence of s(l) to the true von Neumann entropy rate *s* is quantified with the *quantum excess entropy*:

(40) $$\begin{array}{cc} E_{q}\equiv I_{1}^{q} & =\sum_{l=1}^{\infty }\left[\Delta S\left(l\right)-\lim_{l\to \infty }\Delta S\left(l\right)\right]\\ \; & =\sum_{l=1}^{\infty }\left[s(l)-s\right]\;. \end{array}$$

The units for $E_{q}$ are *bits*. Paralleling the classical case, we refer to any qudit process with finite $E_{q}$ as *finitary* and those with infinite $E_{q}$ as *infinitary*.

We can further express $E_{q}$ in terms of the asymptotic behavior of S(l).

**Proposition** **11.** *The quantum excess entropy can be written as*

(41) $$\begin{array}{c} E_{q}=\lim_{l\to \infty }\left[S(l)-sl\right]\;. \end{array}$$

**Proof.**  We evaluate the discrete integral in Equation (40) with Equation (A3) using partial sums:

$$\begin{array}{cc} E_{q} & =\lim_{l\to \infty }\sum_{m=1}^{l}\left[\Delta s(m)-s\right]\\ \; & =\lim_{l\to \infty }\left[S(l)-S(0)-sl\right]\\ \; & =\lim_{l\to \infty }\left[S(l)-sl\right]\;, \end{array}$$

since S(0)=0 by definition. □

For finitary quantum processes, $E_{q}$ is the area between the entropy gain curve and its asymptote *s*, as seen in Figure 3. It also appears in Figure 2 as the vertical offset of the linear asymptote to the S(l) curve.

This leads to a natural scaling of the quantum block entropy as

$$\begin{array}{c} S\left(l\right)∼E_{q}+sl\;, \end{array}$$

as l→∞.

A clearer interpretation of $E_{q}$ as a quantum mutual information is provided by the following proposition.

**Proposition** **12.** *The quantum excess entropy can be written as*

$$\begin{array}{c} E_{q}=\lim_{l\to \infty }S\left(\rho_{-l:0}:\rho_{0:l}\right)\;, \end{array}$$

*where $\rho_{-l:0}$ and $\rho_{0:l}$ are two blocks of ℓ consecutive qudits with a shared boundary.*

**Proof.**  The quantum mutual information, from Equation (19), between two neighboring blocks of *ℓ* qudits can be expressed as

$$\begin{array}{cc} S(\rho_{0:l}:\rho_{-l:0}) & =S\left(\rho_{0:l}\right)-S\left(\rho_{0:l}|\rho_{-l:0}\right)\\ \; & =S\left(l\right)-\sum_{t=0}^{l-1}S\left(\rho_{t}|\rho_{-l:t-1}\right)\;, \end{array}$$

where the final line is obtained through repeated application of Equation (17).

Taking l→∞,

$$\begin{array}{cc} \lim_{l\to \infty }S\left(\rho_{0:l}:\rho_{-l:0}\right)\; & =\lim_{l\to \infty }\left[S\left(l\right)-\sum_{t=0}^{l-1}S\left(\rho_{t}|\rho_{-l:t-1}\right)\right]\\ \; & =\lim_{l\to \infty }\left[S(l)-sl\right]\;, \end{array}$$

where the final line follows from Equation (32) and stationarity.

This final expression is equivalent to the form of $E_{q}$ derived in Proposition 11, concluding the proof. □

$E_{q}$ is therefore a measure of all the correlations between two halves of the infinite sequence of qudits. $E_{q}=0$ if and only if a source is i.i.d. (with S(l)=sl).

We can relate $E_{q}$ for a separable qudit process to $E^{X}$ of the underlying classical process.

**Proposition** **13.** *Let $\overset{↔}{X}$ be a classical process with alphabet X and excess entropy $E^{X}$, and let $|\Psi_{-\infty :\infty }\rangle =E(\overset{↔}{X})$ be a qudit process with alphabet Q and quantum excess entropy $E_{q}$.*

*Then,*

$$\begin{array}{c} E_{q}\leq E^{X}\;, \end{array}$$

*with equality if and only if Q consists of |X| orthogonal states.*

**Proof.**  Consider $X_{-l:l}$, a block of length 2l of the classical process $\overset{↔}{X}$. We can write realizations of $X_{-l:l}$ into a classical register to form the following state:

(42) $$\begin{array}{c} \rho_{-l:l}^{C}=\sum_{x_{-l:l}}\mathrm{Pr}\left(x_{-l:l}\right)|x_{-l:l}\rangle \langle x_{-l:l}|\;, \end{array}$$

where all $|x_{-l:l}\rangle$ are orthogonal. Then, we pass each symbol through the preparation channel E to obtain blocks of our qudit process $\rho_{-l:l}=E^{2l}\left(\rho_{-l:l}^{C}\right)$, where $E^{2l}={⨂}_{i=0}^{2l}E$.

We can express the quantum mutual information as a quantum relative entropy, Equation (20), giving the following relation:

(43) $$\begin{array}{cc} I\left[X_{-l:0}:X_{0:l}\right]\; & =S(\rho_{-l:0}^{C}:\rho_{0:l}^{C})\\ \; & \geq S(E^{l}\left(\rho_{-l:0}^{C}\right):E^{l}\left(\rho_{0:l}^{C}\right))\\ \; & \geq S(\rho_{-l:0}:\rho_{0:l})\;, \end{array}$$

where Equation (43) comes from the monotonicity of the quantum relative entropy in Equation (21). The condition for equality comes from Equation (21) as well. The set of states for which the recovery map must exist is Q, and this is only possible if all $|\psi_{x}\rangle$ are distinguishable—i.e., orthogonal.

Using Equation (A9) to write the excess entropy of $\overset{↔}{X}$ as a limit, we see that

$$\begin{array}{cc} \lim_{l\to \infty }I\left[X_{-l:0}:X_{0:l}\right]\; & \geq \lim_{l\to \infty }S\left(\rho_{-l:0}:\rho_{0:l}\right)\\ E^{X} & \geq E_{q}\;. \end{array}$$

□

A similar bound appears when we apply a repeated POVM measurement to the quantum process to obtain a classical process.

**Proposition** **14.** *Let $\overset{↔}{Y}$ be a measured process such that $\overset{↔}{Y}=M\left(|\Psi_{-\infty :\infty }\rangle \right)$, and let M be a repeated rank-one POVM. Let $\overset{↔}{Y}$ have excess entropy $E^{Y}$, and let $|\Psi_{-\infty :\infty }\rangle$ have quantum excess entropy $E_{q}$. Then,*

$$\begin{array}{c} E_{q}\geq E^{Y}\;, \end{array}$$

*with equality if and only if $|\Psi_{-\infty :\infty }\rangle$ is a separable process with an orthogonal alphabet Q and M uses a POVM whose operators include a projector for each element in Q.*

**Proof.**  Consider $\rho_{-l:l}$—a block of length 2l of the quantum process $|\Psi_{-\infty :\infty }\rangle$—and let M be a repeated measurement of rank-one POVM *M* with elements $\left\{E_{y}\right\}$ so that $M\left(\rho_{t}\right)=\sum_{y}\mathrm{Pr}\left(y\right)|y\rangle \langle y|$, $\mathrm{Pr}\left(y\right)=tr\left(E_{y}\rho_{t}\right)$, and all |y〉 are orthogonal. The repeated measurement applied over a block of length *ℓ* is then $M_{0:l}={⨂}_{i=0}^{l-1}M$.

We express the quantum mutual information as a quantum relative entropy (using Equation (20)) and apply Equation (21) to obtain

(44) $$\begin{array}{c} S\left(\rho_{-l:0}:\rho_{0:l}\right)\geq S\left(M_{0:l}\left(\rho_{-l:0}\right):M_{0:l}\left(\rho_{0:l}\right)\right)\geq S\left(\sum_{y_{-l:0}}\mathrm{Pr}\left(y_{-l:0}\right)\overset{0}{\underset{t=-l}{⨂}}|y_{t}\rangle \langle y_{t}|:\sum_{y_{0:l}}\mathrm{Pr}\left(y_{0:l}\right)\overset{l-1}{\underset{t=0}{⨂}}|y_{t}\rangle \langle y_{t}|\right)\geq I\left[Y_{-l:0}:Y_{0:l}\right]. \end{array}$$

The condition for equality comes from Equation (21) as well. The set of states for which the recovery map must exist is {|y〉}, and this is only possible if all $|\psi_{x}\rangle \in Q$ are orthogonal and *M* contains a projector for each $|\psi_{x}\rangle$. By the same argument as Proposition 4, $|\Psi_{-\infty :\infty }\rangle$ must be a separable process, and Q must consist of orthogonal states.

Taking the limit l→∞, we see that

$$\begin{array}{cc} \lim_{l\to \infty }S\left(\rho_{-l:0}:\rho_{0:l}\right) & \geq \lim_{l\to \infty }I\left[Y_{-l:0}:Y_{0:l}\right]\\ E_{q} & \geq E^{Y}.\; \end{array}$$

□

Combining the above proofs, we obtain the following corollary relating the excess entropies of the underlying classical process $\overset{↔}{X}$ and the measured process $\overset{↔}{Y}$.

**Corollary** **3.** *Let $\overset{↔}{X}$ be a classical process with excess entropy $E^{X}$ and alphabet X, let $|\Psi_{-\infty :\infty }\rangle =E(\overset{↔}{X})$ be a separable qudit process with alphabet Q, and let $\overset{↔}{Y}$ be a measured process with excess entropy $E^{Y}$ such that $\overset{↔}{Y}=M\left(|\Psi_{-\infty :\infty }\rangle \right)$, where M is a repeated rank-one POVM.*

*Then,*

$$\begin{array}{c} E^{X}\geq E^{Y}\;, \end{array}$$

*with equality if and only if Q consists of |X| orthogonal states and M uses a POVM whose operators include a projector for each element in Q.*

**Proof.**  This follows immediately from combining Propositions 13 and 14. □

An exact value of $E_{q}$ typically requires characterizing infinite-length sequences of qudits. However, we can write a finite-*ℓ* estimate of $E_{q}$ using Equation (41):

(45) $$\begin{array}{c} E_{q}\left(l\right)=S\left(l\right)-ls\left(l\right)\;, \end{array}$$

which generally underestimates $E_{q}$’s true value.

### 3.9. Quantum Transient Information

We now turn to look at how the quantum block entropy curve converges to its linear asymptote $E_{q}+sl$. We define the quantum transient information as

(46) $$\begin{array}{c} T_{q}\equiv -I_{0}^{q}=\sum_{l=1}^{\infty }\left[E_{q}+sl-S\left(l\right)\right]\;. \end{array}$$

The units of $T_{q}$ are *bits × time steps*.

$T_{q}$ is represented graphically as the area between the S(l) curve and its linear asymptote for l→∞, as seen in Figure 2. We will see that $T_{q}$ distinguishes between periodic qudit processes that cannot be distinguished with previous information quantities such as $E_{q}$ and *s*.

**Proposition** **15.** *The transient quantum information $T_{q}$ can be written as*

$$\begin{array}{c} T_{q}=\sum_{l=1}^{\infty }l\left[s(l)-s\right]\;. \end{array}$$

**Proof.**  The proof reduces to the straightforward proof for transient information of a classical stochastic process, as defined in Ref. [11]. It depends only upon Equations (46) and (A3), which have the same form in the quantum case as the classical case. □

This expression allows us to estimate $T_{q}$ for a given quantum process as

(47) $$\begin{array}{c} T_{q}\left(l\right)=\sum_{m=1}^{l-1}m\left[s(m)-s(l)\right]\;, \end{array}$$

which generally underestimates the true value of $T_{q}$.

$T_{q}$ is related to the minimal amount of information necessary for an observer to synchronize to an HMCQS. We say an observer is *synchronized* when they are able to determine a source’s internal state. If S(l) converges to its linear asymptote at finite *ℓ*, then there exists an optimal POVM on $\rho_{0:l}$ (in the eigenbasis of $\rho_{0:l}$) that exactly determines the HMCQS’s internal state. Note that this is not guaranteed to be a repeated POVM or even consist of local POVMs. The information within that measurement that is useful for synchronization is quantified by $T_{q}$. In contrast, if S(l) does not converge for finite *ℓ*, then no such POVM over any finite block of qudits exists, and an observer can (at best) only converge asymptotically to the source’s internal state. We will see in Section 5 that even this is not possible for most sources when we restrict ourselves to local measurements.

### 3.10. Quantum Markov Order

The quantum Markov order corresponds to the number of previous qudits on which the next qudit is conditionally dependent. A process has quantum Markov order $R_{q}$ if $R_{q}$ is the smallest value for which the following property holds:

$$\begin{array}{c} S\left(\rho_{0}|\rho_{-R_{q}:0}\right)=S\left(\rho_{0}|\rho_{-\infty :0}\right). \end{array}$$

A graphical interpretation is that when the block size reaches $R_{q}$, S(l) levels off and has a constant slope—which is *s*, as seen in Figure 2. As a consequence, $R_{q}$ is the value of *ℓ* for which ΔS(l) and $\Delta^{2}S\left(l\right)$ converge to their asymptotic values, as seen in Figure 3 and Figure 4, respectively.

Note that a classical process is referred to as ‘Markov’ if R=1. If a separable qudit process has an underlying classical process that is Markov, then it obeys the property in Equation (9) but does not generally have $R_{q}=1$.

Consider a separable quantum process $|\Psi_{-\infty :\infty }\rangle =E(\overset{↔}{X})$, where $\overset{↔}{X}$ has Markov order $R^{X}$, and $|\Psi_{-\infty :\infty }\rangle$ has quantum Markov order $R_{q}$. $R_{q}$ can be equal to, less than, or greater than $R^{X}$. We will give a simple example of each case:

- $R_{q}=R^{X}$ trivially if Q consists of orthogonal states, in which case they have identical block entropy curves via Proposition 3.
- $R_{q}<R^{X}$ if $R^{X}>0$ and all symbols in X are mapped to the same pure state $|\psi_{x}\rangle$. In this case, $R_{q}=0$, as the resulting process is i.i.d.
- $R_{q}>R^{X}$ when $R^{X}>0$, |X|=|Q|, and Q consists of nonorthogonal states. Frequently, $R_{q}=\infty$, since arbitrary sequences of nonorthogonal states cannot reliably be distinguished with a finite POVM.

Similar rules apply when comparing $R_{q}$ to the Markov order $R^{Y}$ of measured process $\overset{↔}{Y}=M\left(|\Psi_{-\infty :\infty }\rangle \right)$:

- $R_{q}=R^{Y}$ if $|\Psi_{-\infty :\infty }\rangle$ is a separable process with an orthogonal alphabet Q and M uses a POVM whose operators include a projector for each element in Q via Proposition 4.
- $R_{q}<R_{Y}$ if Q consists of orthogonal states, $R_{q}=1$, and M is a repeated rank-one POVM that does not include projectors onto the states in Q.
- $R_{q}>R_{Y}$ if $R_{q}>0$ and M is a repeated POVM measurement with the one-element POVM, I. Note that this is not a rank-one POVM.

Now, with a toolbox of quantum information properties in hand, the following section calculates (or estimates) their values for paradigmatic examples of qudit processes.

## 4. Qudit Processes

We now present a variety of separable qudit processes organized roughly by increasing structural complexity and demonstrate the extent to which their behavior can be quantified with Section 3’s informational measures. Their properties are summarized in Table 1 near the end when we have their informational measures in hand. Note that the following results on qudits do not markedly change if one simplifies to qubits.

### 4.1. I.I.D. Processes

Recall that an i.i.d. (independent and identically distributed) qudit process has no classical or quantum correlation between any of the qudits, and its length-*ℓ* density matrices are in a product state such that

$$\begin{array}{c} \rho_{0:l}=\overset{l-1}{\underset{i=0}{⨂}}\rho_{iid}\;, \end{array}$$

where $\rho_{iid}\in H$ is the density matrix for a single time step.

The quantum block entropy takes the form $S\left(l\right)=lS\left(\rho_{iid}\right)$; thus, the von Neumann entropy rate is $s=S(\rho_{iid})$. This implies that a repeated projective measurement exists for which the measured process $\overset{↔}{Y}$ has a classical entropy rate of $h_{\mu }=S\left(\rho_{iid}\right)$. The measurement that realizes this bound consists of orthogonal projectors $P_{y}$, each of which is constructed from an eigenvector of $\rho_{iid}$. In the special case where $\rho_{iid}$ is the maximally mixed state, $s=\log_{2}\left(d\right)$, and any set of orthogonal projectors on a block $\rho_{0:l}$ gives a uniform distribution over all measurement outcomes $y_{0:l}$, since there are no correlations between qudits $E_{q}=0$, $T_{q}=0$, and $R_{q}=0$ trivially. The output of a single-qudit source with uncorrelated noise can be considered an i.i.d. qudit process.

### 4.2. Quantum Presentations of Classical Processes

Any classical process with alphabet X can be represented by a qudit process with orthogonal alphabet Q, where each symbol x∈X corresponds to a pure state $|\psi_{x}\rangle \in Q$. In this case, the encoding E is trivial, and one can recover the underlying process $\overset{↔}{X}$ via repeated measurement with orthogonal projectors $\left\{P_{x}=|\psi_{x}\rangle \langle \psi_{x}|\right\}$. This requires that d≥|X|.

The information measures for the underlying process, the qudit process, and measurement outcomes obey

$$\begin{array}{cc} H\left[X_{0:l}\right] & =S(\rho_{0:l})\\ \; & \leq H\left[Y_{0:l}\right]\;, \end{array}$$

with equality for repeated measurement with a rank-one POVM whose elements include the projector set $\left\{P_{x}\right\}$.

From this relation, we can see that many quantum information quantities such as *s*, $E_{q}$, $T_{q}$, and $R_{q}$ are equal to the classical properties of $\overset{↔}{X}$. Exceptions include quantities that depend on the relationship between *d* and |X|, such as quantum redundancy.

Since the quantum-classical channel E is trivial, the output process $\overset{↔}{Y}$ is the result of passing $\overset{↔}{X}$ through a noisy classical channel, where the level of noise depends on the particular measurement scheme M.

For a repeated rank-one POVM, $H\left[X_{0:l}\right]\leq H\left[Y_{0:l}\right]$ for all *ℓ* from Propositions 3 and 4. Similar inequalities explicitly relate other classical process properties such as the entropy rates ($h_{\mu }^{X}\leq h_{\mu }^{Y}$), the predictabilities ($\left|G^{X}|\geq |G^{Y}\right|$), and the excess entropies (see Corollary 3).

### 4.3. Periodic Processes

A classical stochastic process $\overset{↔}{X}$ is periodic with period *p* if it consists of repetitions of a template sequence—a length-*p* block of symbols. A periodic separable qudit process $|\Psi_{-\infty :\infty }\rangle =E(\overset{↔}{X})$ is one for which the underlying classical process $\overset{↔}{X}$ is periodic.

For classical periodic processes, the block entropy curve reaches a maximal value at its Markov order R=p and thereafter remains constant with increasing *ℓ* ($h_{\mu }=0$). That maximal value is the excess entropy, which is entirely determined by the period according to the formula $E=\log_{2}\left(p\right)$. Finally, an observer attempting to synchronize to different length-*p* templates may encounter more or less uncertainty in the process depending on the template itself, which is a feature captured by the transient information T [11].

**Figure 5:**
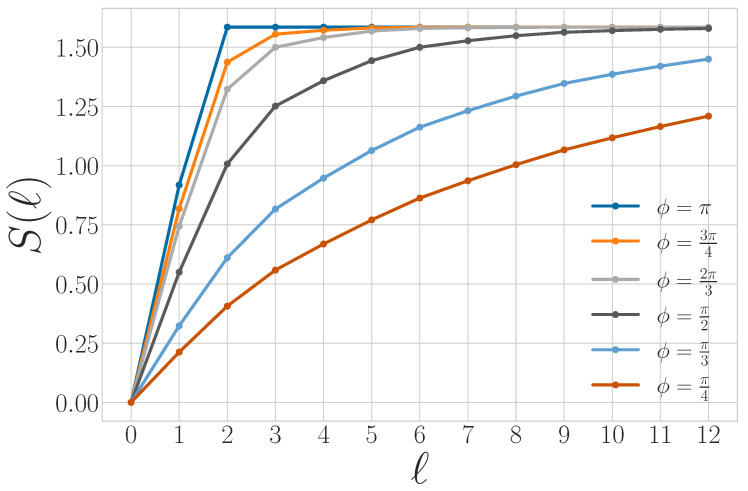
Quantum block entropies S(l) versus length *ℓ* for the periodic process emitting the state $|\psi_{00\phi }\rangle$ with different values of ϕ. Each curve approaches a maximum value of $E_{q}=\log_{2}3$. Larger values of ϕ correspond to more distinguishable alphabet states and lower values of $T_{q}$.

Periodic qudit processes share many of these properties (as we show via Section 3’s results) but also exhibit richer behavior, since they can consist of nonorthogonal qudit states. Figure 5 shows the quantum block entropies for the period-3 process consisting of the repeated quantum word $|\psi_{00\phi }\rangle =|0\rangle ⊗|0\rangle ⊗|\psi (\phi )\rangle$, where $|\psi (\phi )\rangle =\cos\frac{\phi }{2}|0\rangle +\sin\frac{\phi }{2}|1\rangle$. For ϕ = π, we recover the block entropy for the classical period-3 word ‘001’. As ϕ decreases, |ψ(ϕ)〉 becomes less distinguishable from |0〉.

From Propositions 6 and 13, it follows that periodic qudit processes with period *p* have s=0 and $E_{q}\leq \log_{2}\left(p\right)$. (And, $E_{q}=\log_{2}\left(p\right)$ unless two classical symbols in X are sent to the same pure state in Q in a way that reduces the effective period of the qudit process to less than *p*). However, the quantum block entropy curve does not necessarily reach its maximal value of $E_{q}$ for l=p if Q contains nonorthogonal states. In this case, $R_{q}\to \infty$, since an observer cannot unambiguously distinguish where one length-*p* block begins and another one ends with any finite measurement.

Though all period-*p* qudit processes have the same von Neumann entropy rate and quantum excess entropy, they may be distinguished by their values for the quantum transient information in two different ways.

First, different quantum alphabets Q give different values of $T_{q}$. Figure 5 demonstrates that, for a two-state qubit alphabet, as the states become more or less distinguishable, the quantum transient information increases. For ϕ=π (orthogonal alphabet), $T_{q}\approx 2.33$ bits × time steps, whereas for $\phi =\frac{\pi }{2}$, $T_{q}\approx 4.22$ bits × time steps. These values of $T_{q}$ (and others in this section) are numerically approximated using Equation (47), with l=12.

Second, $T_{q}$ can distinguish between different length-*p* words. Reference [11] shows that T can distinguish between different period-5 classical words (‘00001’, ‘00011’, and ‘00101’), and $T_{q}$ generalizes this behavior. Whereas all period-3 words are equivalent to ‘001’ under global bit swap and translations, the same is not true for period-5 words. Section 5 discusses synchronizing to period-5 qudit sources in more detail and relates that task to the value of $T_{q}$.

### 4.4. Quantum Golden Mean Processes

The classical Golden Mean process consists of all binary strings with no consecutive ‘1’s. It is a Markov process (R=1), since the joint probabilities $\mathrm{Pr}(X_{0:l})$ for blocks factor as in Equation (2), with $\mathrm{Pr}\left(0|0\right)=\frac{1}{2}$, $\mathrm{Pr}\left(1|0\right)=\frac{1}{2}$, Pr(0|1)=1, and Pr(1|1)=0. For the classical Golden Mean, $h_{\mu }=\frac{2}{3}$ bits per symbol and E≈0.2516 bits.

**Figure 6:**
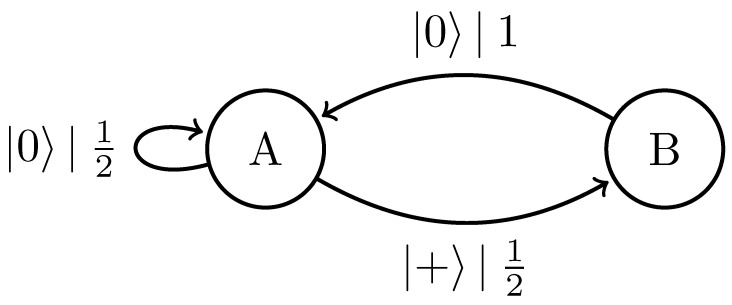
|0〉-|+〉 Quantum Golden Mean process generator.

Replacing the classical symbol alphabet with the quantum alphabet Q={|0〉,|+〉}, where $|+\rangle =\frac{1}{\sqrt{2}}\left(|0\rangle +|1\rangle \right)$, gives the |0〉-|+〉 Quantum Golden Mean process introduced in Ref. [14]. Figure 6 shows its generator.

**Figure 7:**
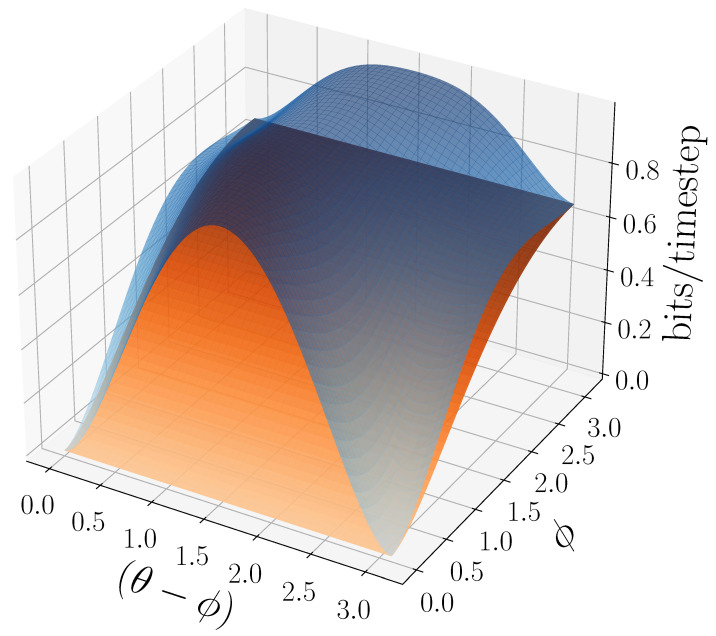
von Neumann entropy rate *s* (lower, orange surface) and measured entropy rate $h_{\mu }^{Y}$ (higher, blue surface) for the |0〉-|ϕ〉 Quantum Golden Mean process measured with the repeated PVM $M_{\theta }$. *s* increases as ϕ does, and the alphabet becomes more distinguishable. For ϕ=π and θ=0,π, we recover the classical Golden Mean. For $\left(\theta -\phi \right)=\frac{\pi }{2}$, $M_{\theta }$ applied to |ϕ〉 is a maximum entropy PVM (distribution of measurement outcomes is 50-50). Maxima of $h_{\mu }^{Y}$ lie in this region.

We can further generalize this process to the |0〉-|ψ(ϕ)〉 Quantum Golden Mean process with quantum alphabet $\left\{|0\rangle ,|\psi (\phi )\rangle =\cos\frac{\phi }{2}|0\rangle +\sin\frac{\phi }{2}|1\rangle \right\}$. This process’s quantum entropy rate is shown in Figure 7 for different values of ϕ. For ϕ=π, |ψ(ϕ)〉=|1〉, and we recover the classical Golden Mean process. As ϕ decreases to 0, the states in Q become less distinguishable and *s* decreases, as is expected from Proposition 6.

Also in Figure 7, we see the entropy rate $h_{\mu }^{Y}$ of the measured processes obtained by applying a repeated PVM to the |0〉-|ψ(ϕ)〉 Quantum Golden Mean process. This PVM consists of projectors parametrized by the angle θ, where $M_{\theta }=\left\{|\psi (\theta )\rangle \langle \psi (\theta )|,|\psi (\theta +\pi )\rangle \langle \psi (\theta +\pi )|\right\}$. As mandated by Proposition 7, $h_{\mu }^{Y}\geq s$ for all ϕ and θ.

**Figure 8:**
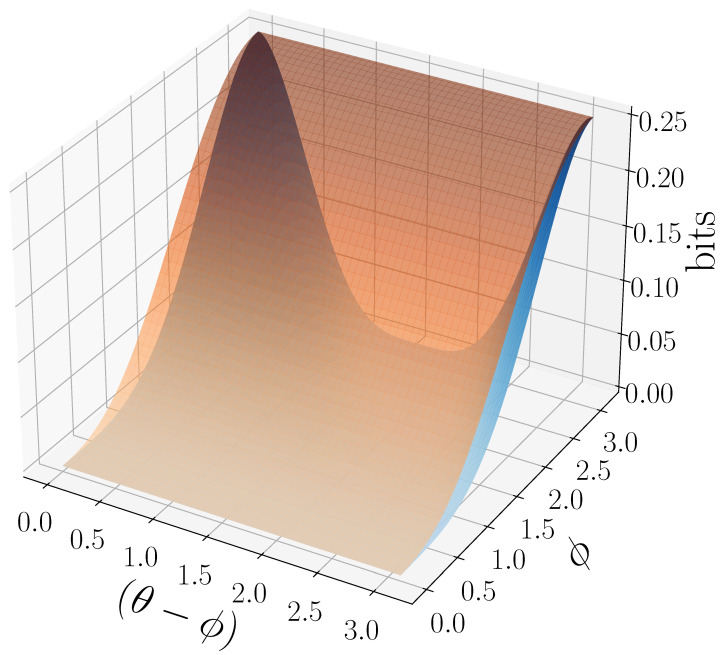
Quantum excess entropy $E_{q}$ (higher, orange surface) and measured excess entropy $E^{Y}$ (lower, blue surface) for the |0〉-|ϕ〉 Quantum Golden Mean process measured with repeated PVM $M_{\theta }$. $E_{q}$ increases with ϕ, since |0〉 and |ϕ〉 become more distinguishable. $E_{Y}$ is maximized for $\left(\theta -\phi \right)=0,\pi$, since $M_{\theta }$ can best determine if |ϕ〉 was emitted.

Figure 8 demonstrates how another of Section 3’s quantum information properties, the quantum excess entropy $E_{q}$, relates to the excess entropy $E^{Y}$ of the classical measured process. For all ϕ and θ, the bound from Proposition 14 ($E_{q}\geq E^{Y}$) holds, $E^{Y}$ has maxima at ϕ=π, and θ=0,π when Q={|0〉,|1〉} and $M=M_{01}$. These quantities were estimated using l=10.

Underlying the |0〉-|ψ(ϕ)〉 Quantum Golden Mean is the classical Golden Mean process, which is Markov. Thus, it obeys the quantum Markov property of Equation (9) despite the fact that it has an infinite quantum Markov order for most values of ϕ. This has implications for an observer’s ability to synchronize to a Quantum Golden Mean source, which Section 5 explores.

### 4.5. 3-Symbol Quantum Golden Mean

**Figure 9:**
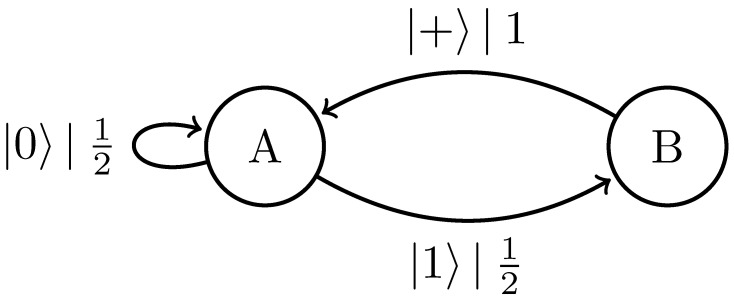
3-Symbol Quantum Golden Mean process generator.

Figure 9 shows the generator of the 3-Symbol Quantum Golden Mean process with alphabet Q={|0〉,|1〉,|+〉}. Though its generator shares the same internal states and transition probabilities as the |0〉-|+〉 Quantum Golden Mean, the 3-Symbol Quantum Golden Mean does not have a one-to-one correspondence between the quantum alphabet Q and the generator states S (|0〉→A and |+〉→B). Instead, |Q|=3, and these three states cannot all be orthogonal with d=2.

However, unlike the |0〉-|+〉 Quantum Golden Mean, we can calculate the quantum entropy rate directly from the generator because (1) it has the property of *quantum unifilarity*, and (2) it is possible to synchronize to it. Both are discussed at length in Section 5. For now, an HMCQS is *quantum unifilar* if and only if, for every σ∈S, there exists some POVM $M_{\sigma }$ such that an observer knowing σ and the outcome of $M_{\sigma }$ can uniquely determine the internal state to which the HMCQS transitioned. The generator in Figure 9 meets this criterion with $M_{A}=M_{01}$. $M_{B}$ can be any POVM.

For a classical, unifilar HMC, $h_{\mu }$ can be calculated as

(48) $$\begin{array}{c} h_{\mu }=\sum_{\sigma_{i},\sigma_{j}\in S}\pi_{i}\sum_{x\in X}T_{ij}^{x}\log_{2}T_{ij}^{x}\;, \end{array}$$

where π is the stationary state distribution. This result dates back to the foundations of information theory [7].

Similarly, for a quantum unifilar HMCQS to which one can synchronize, we can write

(49) $$\begin{array}{c} s=\sum_{\sigma_{i},\sigma_{j}\in S}\pi_{i}\sum_{x\in X}T_{ij}^{x}\log_{2}T_{ij}^{x}\;. \end{array}$$

Let us walk through this logic for the 3-Symbol Quantum Golden Mean. In state *A* ($\pi_{A}=\frac{2}{3}$), the generator emits a qubit either in state |0〉 or |1〉, each with probability $\frac{1}{2}$. The density matrix describing this qubit is a maximally mixed state, and any measurement performed on it involves 1 bit of irreducible randomness. If it is in state *B* ($\pi_{B}=\frac{1}{3}$), it emits a qubit in state |+〉 deterministically. Thus, averaging over the states, the von Neumann entropy rate is $s=\frac{2}{3}$ bits/time step.

**Figure 10:**
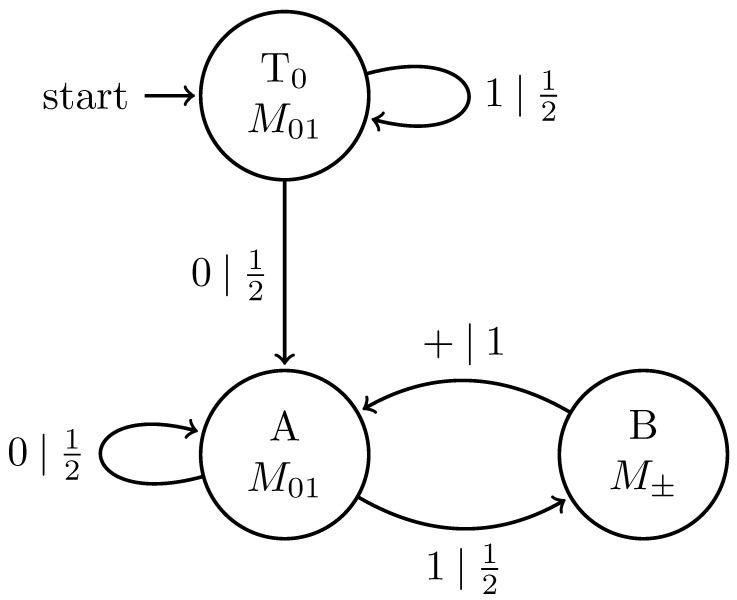
Adaptive measurement protocol (in the form of a DQMP) for the 3-Symbol Quantum Golden Mean process. To synchronize, an observer starts in $T_{0}$ (a transient state) and measures with $M_{01}$. The probability of observing exactly *n* ‘1’s is $\frac{1}{2^{n}}$. Upon observing a ‘0’, the observer synchronizes. States *A* and *B* correspond exactly to internal states *A* and *B* of the source in Figure 9. The ‘−’ transition is not displayed because it has probability 0. The source is quantum unifilar; thus, one stays synchronized for future times.

Despite this, when restricted to measuring with a repeated rank-one POVM, the entropy rate of the observed classical process cannot reach the lower bound of $\frac{2}{3}$ bits per symbol because the minimum entropy basis when in state *A* is $M_{01}$, while the minimum entropy basis when in state *B* is $M_{\pm }$. However, an experimenter using the adaptive measurement protocol in Figure 10 would observe symbol sequences with an entropy rate of $\frac{2}{3}$ bits per symbol once they have synchronized.

They start by measuring in the $M_{01}=\left\{|0\rangle ,|1\rangle \right\}$ basis and use the outcome of the initial measurement to select a new basis. If the outcome is ‘0’, they are able to synchronize to the process generator, which is necessarily in state *A*. They continue in state *A* measuring with POVM $M_{01}$ until they observe a ‘1’, at which point they transition to *B* and measure with $M_{\pm }$, which is a zero-entropy measurement. They observe a ‘+’ and return to state *A*.

In this way, when using an adaptive measurement protocol, the recurrent part $\overset{↔}{X}[Y_{r}]$ of the measurement sequence may have a lower entropy rate than $h_{\mu }^{Y}$ for any repeated rank-one POVM measurement, even if the associated DQMP uses only rank-one PVMs, as in this example. These ideas are formalized and expanded upon in the next section.

### 4.6. Unifilar and Nonunifilar Qubit Sources

**Figure 11:**
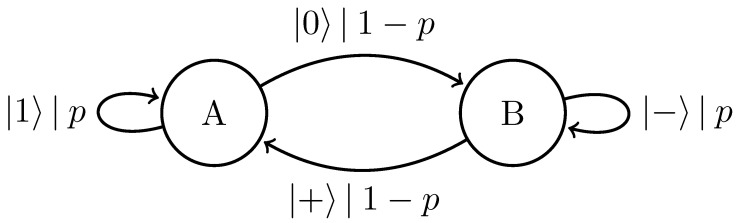
Unifilar qubit source. Each internal state emits one of two orthogonal states and then transitions—e.g., *A* emits either |0〉 or |1〉 that can be distinguished by measurement $M_{01}$—giving this source the property of quantum unifilarity. *p* is a parameter that takes values from 0 to 1. Other processes correspond to particular *p* values: for example, a nonorthogonal period-2 process (p=0), the maximally mixed i.i.d. process ($p=\frac{1}{2}$), and a deterministic sequence of either |1〉 or |−〉 (p=1).

**Figure 12:**
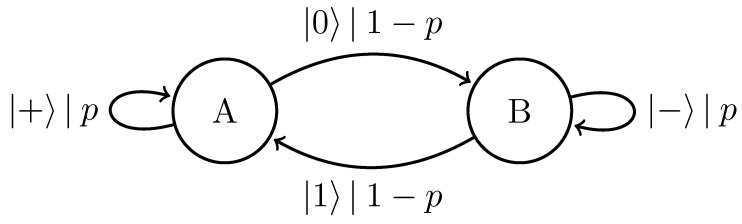
Nonunifilar qubit source. Each internal state emits one of two nonorthogonal states and then transitions. An observer will not be able to determine which state the source transitioned to with any POVM. *p* takes values from 0 to 1. Other processes correspond to particular p values: for example, an orthogonal period-2 process (p=0), the maximally mixed i.i.d. process ($p=\frac{1}{2}$), and a deterministic sequence of either |1〉 or |−〉 (p=1).

The last two classes of qubit processes to discuss are generated by the unifilar and nonunifilar qubit sources shown in Figure 11 and Figure 12, respectively. Both of these generators consist of two internal states (S={A,B}) and emit four possible pure-qubit states (Q={|0〉,|1〉,|+〉,|−〉}) that form two orthogonal pairs. However, for each internal state of the unifilar qubit source, the two possible emitted states are orthogonal, and one can unambiguously determine the next internal state. In other words, it has the property of quantum unifilarity. The same is not true of nonunifilar qubit sources. The next section illustrates how this difference strongly affects an observer’s ability to synchronize.

By varying the parameter *p*, one can interpolate between one of the several simpler processes already analyzed. Starting with the unifilar qubit source in Figure 11, for p=0, the generator becomes a period-2 source that emits the word $|\psi_{0+}\rangle$. For $p=\frac{1}{2}$, we obtain a two-state model that generates the i.i.d. maximally mixed process. And, as p→1, the generator emits longer strings of either only |1〉 or only |−〉 qubits depending on whether the source is in *A* or *B*. At p=1, the process becomes nonergodic (here demonstrated by the disconnection between internal states), and the source will only emit either |1〉 or |−〉 deterministically.

Similarly, the nonunifilar qubit source in Figure 12 simplifies for certain *p*’s. For p=0, we obtain a period-2 source, this time with sequence $|\psi_{01}\rangle$, and for $p=\frac{1}{2}$, it also generates the i.i.d. maximally mixed process. As p→1, it emits long sequences of either all |+〉 or all |−〉 and becomes nonergodic for p=1.

### 4.7. Unifilar Qutrit Source

**Figure 13:**
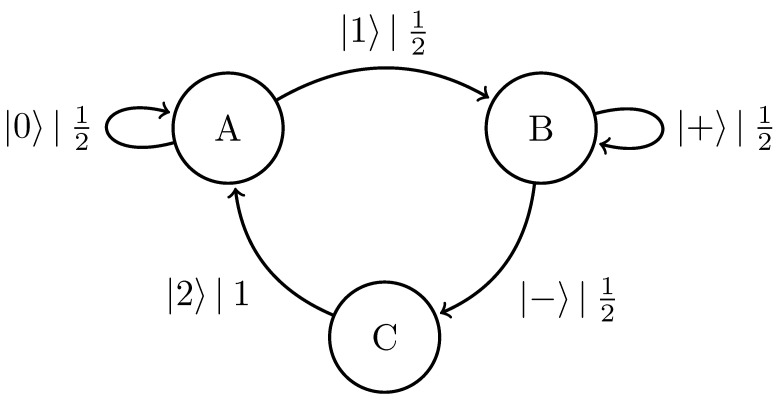
Unifilar qutrit source. When in internal states *A* and *B*, it emits a qutrit in the subspace of Hilbert space spanned by |0〉 and |1〉. When in *C*, it emits |2〉, which can always be distinguished from all other states in Q. This demonstrates additional opportunities for synchronization in higher-dimensional Hilbert spaces.

Expanding beyond processes over qubits, we consider a single example of a qutrit (d=3) process whose generator is shown in Figure 13 and that employs a five-qubit alphabet Q={|0〉,|1〉,|2〉,|+〉,|−〉}. Using a higher-dimensional Hilbert space makes more measurements available to an observer for synchronization. As a consequence, this example process exhibits behavior that is impossible with qubit processes alone: A subspace of Hilbert space (occupied by |2〉) is always distinguishable from all other states in Q and can be reserved for synchronization. The other subspace (including |0〉, |1〉, |+〉, and |−〉) consists of states that cannot be reliably distinguished. Note that this process is quantum unifilar, but one cannot remain synchronized by measuring in a single basis. The next section discusses multiple adaptive measurement protocols that can do so.

### 4.8. Discussion

Table 1 summarizes information properties for the above examples with either analytic results or numerical estimates. Together, these examples illustrate a range of different features of separable qudit processes. They demonstrate how the information properties defined and characterized in Section 3 are both indicative of underlying structural features of quantum information sources and strongly influenced by the distinguishability of states in a process’s quantum alphabet. Our analysis of the convergence of the quantum block entropy to its linear asymptote thus gives meaningful and interpretable ways of quantifying the randomness and correlation in a separable quantum process.

We continue by discussing two tasks that an observer might wish to perform when faced with a quantum information source emitting a separable quantum process. First, if they have prior knowledge of the internal structure of the source, they may want to determine the internal state it occupies during a given time step. This task is *synchronization*, and we discuss it in the next section. Second, if they have no knowledge of the source, they may want to measure the process it produces to infer its internal structure. This task is *system identification*, and we demonstrate how an observer can use a tomographic protocol to perform it in Section 6.

**Table 1:** Information properties for example quantum processes/sources. Decimal values were numerically estimated using Equations (38), (45) and (47), with l=8 for the unifilar qutrit source, l=10 for the unifilar and nonunifilar qubit sources, and l=12 for all other processes. Other values were calculated analytically.

	*s*	$|G_{q}|$	$E_{q}$	$T_{q}$	$R_{q}$
	(***Bits/Time Step***)		(***Bits***)	(***Bits** × **Time Steps***)	(***Time Steps***)
I.I.D. Qubit Process	$S(\rho_{iid})$	$1-S(\rho_{iid})$	0	0	0
Period-3 Process (ϕ=π)	0	1	$\log_{2}\left(3\right)$	2.33	3
Period-3 Process (ϕ=π/2)	0	1	$\log_{2}\left(3\right)$	4.22	*∞*
Quantum Golden Mean (ϕ=π)	2/3	1/3	0.2516	1/3	1
Quantum Golden Mean (ϕ=π/2)	0.4495	0.5505	0.1092	0.5687	*∞*
3-Symbol Quantum Golden Mean	0.6667	0.3333	0.4652	0.8855	*∞*
Unifilar Qubit Source (p=1/3)	0.9184	0.0816	0.0808	0.1976	*∞*
Nonunifilar Qubit Source (p=1/3)	0.7306	0.2614	0.3217	0.4090	*∞*
Unifilar Qutrit Source	0.8002	0.7848	1.290	2.156	*∞*

## 5. Synchronizing to a Quantum Source

How does an observer of a process with knowledge of its quantum generator determine its internal state? When an observer is certain about the internal state, the observer is *synchronized* to the quantum source. The following explores synchronizing to quantum processes—both the manner in which observations lead to inferring the source’s state and quantitative measures of partial and full synchronization.

Quantum measurement adds subtlety to this task in comparison to the task of synchronizing to a classical process given knowledge of its minimal unifilar model—the ε-machine—as described in Refs. [12,47].

### 5.1. States of Knowledge

Recall that a hidden Markov chain quantum source (HMCQS) consists of a set of internal states (S), a pure-state alphabet (Q), and a set of labeled transition matrices (T). We assume an observer has complete knowledge of the HMCQS that generates a process but can only infer the internal state at time t=l by applying block measurement $M_{0:l}$ and observing outcome $y_{0:l}$. They have no access to the qudits that were emitted before t=0.

An observer’s best guess for the internal state of a source given different sequences of observations can be represented as distributions over the source’s internal states, which are known as *mixed states* (not to be confused with mixed quantum states, which are represented by density matrices). Classically, after observing a particular length-*ℓ* word $w=x_{0:l}$, the observer is in the mixed state $\eta \left(w\right)=\left(p_{A},p_{B},\ldots \right)$. After the next observation $X_{l}$, they will transition to one of a set of new mixed states depending on the outcome $\eta (w,x_{l}=0)$, $\eta (w,x_{l}=1)$, and so on. The word corresponding to the new mixed state is a concatenation of *w* and the new observation $x_{l}$. The set of mixed states for a classical process and the dynamic between them define a process’s *mixed state presentation* (MSP), which is unifilar by construction [48].

Using a classical process’s MSP rather than a nonunifilar generator of the process has many computational advantages. Two of interest are that it allows one to calculate the entropy rate for processes without finite unifilar presentations [49] and that it allows one to calculate the uncertainty an observer experiences while attempting to synchronize to a process’s generator [50].

For quantum processes, there is no unique MSP but rather a multiplicity of possible MSPs, each corresponding to a different choice of measurement protocol. For a given source and given measurement protocol M, we can define a set of mixed states with each corresponding to the possible measurement sequence one can observe.

Consider the mixed states corresponding to length-*ℓ* sequences of observations. We restrict $M_{0:l}$ to consist of local POVMs, allowing for adaptive measurement. Given that an observer has applied measurement $M_{0:l}$ and seen measurement outcomes $y_{0:l}$, their best guess about the generator’s internal state is represented by the conditional distribution $\eta \left(y_{0:l}|M_{0:l}\right)=\left\{\mathrm{Pr}\left(\sigma |y_{0:l},M_{0:l}\right)\;\mathrm{for}\;\mathrm{all}\;\sigma \right\}$.

For t=0, an observer has no measurement outcomes with which to inform their prediction about the source’s internal state. However, they do know the stationary state distribution π of the model (since it can be calculated directly from T). This serves as a ‘best guess’ of the source’s internal state absent any measurements. If the initial state distribution $S_{0}$ is not π and the observer is aware of this fact, the mixed states for that process are $\eta (y_{0:l}|M,S_{0})$. We omit the conditioning on $S_{0}$ if $S_{0}=\pi$.

For most repeated PVM measurements of qubit processes, there are an uncountably infinite number of mixed states [14]. This *measurement-induced complexity* appears even for |S|=2. The examples in this section step back from this complexity to focus on measurements that are sufficiently informative to allow an observer to synchronize with finite observation sequence $y_{0:l}$ and result in only finite or (at most) a countably infinite set of recurrent mixed states.

### 5.2. Average State Uncertainty and Synchronization Information

To compare synchronization behavior for different measurement protocols, it is useful to use entropic quantities rather than working with the set of mixed states and their dynamic directly. In particular, we look at the entropy of the possible mixed states for length-*ℓ* sequences of measurements.

An observer’s uncertainty about the source’s internal state after applying measurement sequence $M_{0:l}$ (according to some protocol M) and observing outcome $y_{0:l}$ is given by the Shannon entropy of the corresponding mixed-state distribution:

$$\begin{array}{cc} H\left[\eta \left(y_{0:l}|M\right)\right] & =H\left[\mathrm{Pr}\left(\sigma |y_{0:l},M\right)\right]\\ \; & =-\sum_{\sigma \in S}\mathrm{Pr}\left(\sigma |y_{0:l},M\right)\log_{2}\mathrm{Pr}\left(\sigma |y_{0:l},M\right)\;. \end{array}$$

One can average over all measurement outcomes on a block of *ℓ* qudits to find the *average state uncertainty*:

$$\begin{array}{cc} H(l|M) & \equiv H\left[\eta \left(Y_{0:l}|M\right)\right]\\ \; & \equiv -\sum_{y_{0:l}}\mathrm{Pr}\left(y_{0:l}|M\right)H\left[\eta \left(y_{0:l}|M\right)\right]\;. \end{array}$$

For a given measurement protocol M, this quantity generally converges to a finite value in the l→∞ limit. This limit does not necessarily exist if the dynamics are not ergodic, for example, if there is some underlying periodicity. If such a limit exists, the *asymptotic state uncertainty* is

$$\begin{array}{c} C_{\infty }\left(M\right)=\lim_{l\to \infty }H\left(l|M\right)\;. \end{array}$$

Both H(l|M) and $C_{\infty }\left(M\right)$ are measured in bits.

If $H\left[\eta \left(y_{0:l}|M\right)\right]=0$, then $y_{0:l}$ is a *synchronizing observation*, and an observer who sees outcome $y_{0:l}$ can precisely identify the source’s internal state. If H(l|M)=0, then any measurement outcome seen when applying protocol M to *ℓ* qudits allows the observer to synchronize to the source. For classical and quantum processes, the Markov order is the first value of *ℓ* for which H(l) vanishes (for some $M_{0:l}$ in the quantum case). This generally does not occur for HMCQS with nonorthogonal states in Q except in the l→∞ limit, since $R_{q}$ is generally infinite.

Being synchronized after measuring *ℓ* qudits does not guarantee that the observer remains synchronized after measuring qudit l+1. In classical processes for which this occurs, persistent synchronization requires that the HMC of the process that the observer uses satisfies the additional condition of *unifilarity*. In the quantum setting, synchronization persists if and only if the underlying HMCQS is *quantum unifilar*, and when the observer is in internal state σ, their measurement protocol ensures they apply the measurement for which the internal state at time t+1 is completely determined.

This is equivalent to the statement ‘There exists some protocol M with measurements $M_{0:l}$ and $M_{l}$ such that

(50) $$\begin{array}{cc} \; & H\left[\mathrm{Pr}\left(\sigma |y_{0:l},M_{0:l}\right)\right]=0\\ \; & \;\Rightarrow H\left[\mathrm{Pr}\left(\sigma |y_{0:l}y_{l},M_{0:l}⊗M_{l}\right)\right]=0\;, \end{array}$$

for all $y_{l}\in Y$’.

The measurement that maintains synchronization when the source is in one internal state ($\sigma_{i}$) does not need to be the same as the measurement that maintains synchronization when the source is in another internal state ($\sigma_{j}$). When the measurement required to maintain synchronization depends upon the HMCQS’s current state, then *adaptive measurement protocols* are capable of maintaining synchronization even when no fixed-basis measurement can, as the following demonstrates with multiple examples.

Finally, the total amount of state uncertainty that an observer encounters while synchronizing to a process using a given measurement protocol M is the *synchronization information*, which is given by

(51) $$\begin{array}{c} S\left(M\right)=\sum_{l=0}^{\infty }H\left(l|M\right)\;. \end{array}$$

Note that, if the asymptotic state uncertainty $C_{\infty }\left(M\right)$ is greater than 0, S(M) is infinite. When $C_{\infty }\left(M\right)=0$, we can estimate S(M) by terminating the sum at some finite *ℓ*.

For classical processes, the synchronization information is closely tied to the transient information T. We will similarly connect S(M) to the quantum transient information $T_{q}$ and use it as a way to compare synchronization via different measurement protocols.

The remainder of this section explores synchronization to various models from Section 4. We pair each with a variety of measurements, both repeated POVMs and adaptive protocols, to demonstrate the way H(l|M) is affected by this choice.

### 5.3. Synchronizing to Quantum Presentations of Classical Processes

An HMCQS with a qudit alphabet consisting entirely of orthogonal states ($\langle \psi_{x}|\psi_{x^{\prime }}\rangle =0$, for all $|\psi_{x}\rangle$ and $|\psi_{x^{\prime }}\rangle \in Q$), is a quantum presentation of the underlying classical process $\overset{↔}{X}$. An observer can perform a measurement using orthogonal projectors $P_{x}=|\psi_{x}\rangle \langle \psi_{x}|$ for all $|\psi_{x}\rangle \in Q$ that unambiguously discriminates between the pure qudit states the source emits.

The task of synchronizing to such an HMCQS is equivalent to synchronizing to a source emitting classical symbols. If it is quantum unifilar, then the underlying classical HMC is unifilar, and synchronization is exponentially fast (on average) [12,47]. Absent unifilarity, an observer may not be able to synchronize even asymptotically—i.e., $C_{\infty }\left(M\right)>0$ for all M—despite measuring with orthogonal projectors.

### 5.4. Synchronizing to Periodic Processes

All periodic quantum processes have a vanishing von Neumann entropy rate—i.e., s=0—and each state only has one incoming and one outgoing transition. Due to these simplifications, an observer simply determines the source’s *phase* to synchronize; i.e., which of the *p* internal states the source occupies at time *t*. Once this phase is determined for any *t*, it is also determined for all other *t*. The initial phase uncertainty is equivalent to the initial state uncertainty $H\left[\pi \right]=\log_{2}\left(p\right)$, since π is uniform.

Any measurement protocol M that distinguishes between states in Q gives information about the phase and allows an observer to synchronize to the source asymptotically. If two states in Q cannot be distinguished by M, then synchronization may not be possible, even with an infinite number of measurements.

For classical periodic processes, an observer synchronizes at the Markov order l=p, and the synchronization information and the transient information are equal: S=T [11]. In contrast, quantum periodic processes generically have infinite Markov order, if there are nonorthogonal states in Q, and only asymptotically synchronize. Thus, $S\left(M\right)\geq T_{q}$. The condition for equality is that M is the optimal measurement over the process. For $R_{q}=\infty$, this means that M must be a global measurement over the bi-infinite chain of qudits.

**Figure 14:**
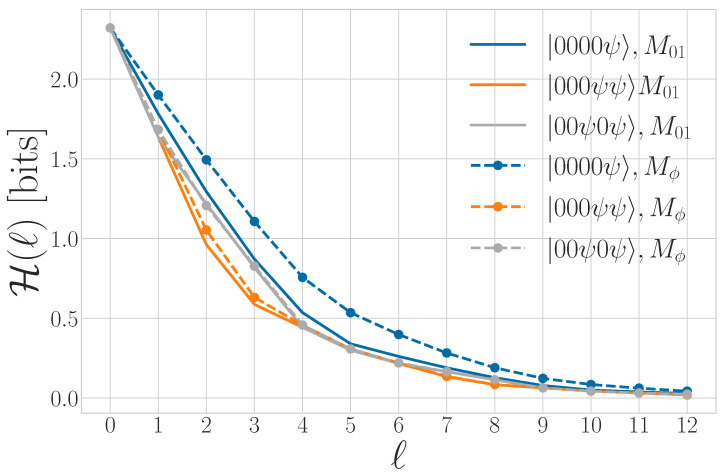
Average state uncertainty H(l) for period-5 qudit processes: ψ denotes state |ψ(ϕ)〉. The associated PVM is $M_{\phi }=\left\{|\psi (\phi )\rangle ,|\psi_{(}\phi +\pi )\rangle \right\}$. We set ϕ=3π/4. The area under each curve is the synchronization information S for that process and measurement.

Figure 14 shows the average state uncertainty for an observer measuring three different period-5 qubit processes consisting of two alphabet states: |0〉 and |ψ(ϕ=3π/4)〉. All other period-5 sequences with this qubit alphabet are equivalent to these three under shift and swap symmetries. As expected, H(l) decreases from $H\left(0\right)=\log_{2}5$ to $C_{\infty }=0$ for all combinations of sequence and measurement basis. That noted, the rate at which synchronization occurs—and the total amount of uncertainty seen by an observer, the synchronization information—depends on both the particular sequence and the particular repeated measurement applied to it.

For period-5 process with word ‘0000ψ’, $T_{q}\approx 6.32$ bits × time steps, using Equation (47), with l=12. When measuring it in basis $M_{01}$ ($M_{\phi }$), we find that $S(M_{01})\approx 7.92$ bits ($S(M_{\phi })\approx 9.30$ bits). These are also estimated by calculating up to l=12. Similarly, the bound between S(M) and $T_{q}$ holds for the other period-5 words. For word ‘000ψψ’, $T_{q}\approx 4.86$ bits × time steps, $S(M_{01})\approx 6.84$ bits, and $S(M_{\phi })\approx 7.05$ bits. For word ‘00ψ0ψ’, $T_{q}\approx 5.51$ bits × time steps, $S(M_{01})\approx 7.39$ bits, and $S(M_{\phi })\approx 7.47$ bits.

At this point, we wish to emphasize that *s*, $E_{q}$, and $R_{q}$ are equal for generic period-5 processes with nonorthogonal alphabets Q. Despite this, $T_{q}$ and S(M) identify physically relevant differences between these processes for the task of synchronization. These differences are intrinsic to the quantum process itself ($T_{q}$) and also appear within the measurement outcomes an observer obtains (S(M)).

As a final comment on periodic processes, we note that while s=0 for all periodic quantum process, many measurement protocols (even those for which $C_{\infty }=0$) give a measured classical process with a nonzero entropy rate $h_{\mu }^{Y}$. In fact, no fixed-basis measurement of a periodic process results in an observed process with $h_{\mu }^{Y}=0$ unless (i) all states in Q are orthogonal and (ii) the measurement is in an orthogonal basis which includes one projector for each state in Q.

**Figure 15:**
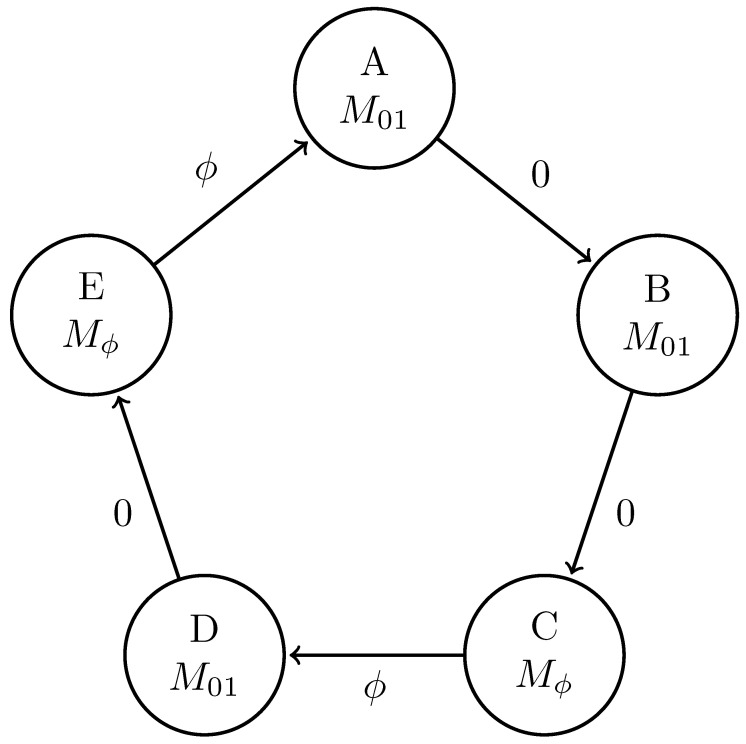
Adaptive measurement protocol for period-5 sequence |00ψ0ψ〉. Each state is labeled with the next measurement to perform, either $M_{01}$, with possible outcomes ‘0’ and ‘1’, or $M_{\phi }$, the PVM with elements |ψ(ϕ)〉〈ψ(ϕ)| and |ψ(ϕ+π)〉〈ψ(ϕ+π)| and possible outcomes ϕ and $\left(\phi +\pi \right)$. The five states shown are the measurement protocol’s recurrent states that an observer only encounters when synchronized. An observer who is synchronized and using this protocol sees a measured period-5 process with word ‘00ϕ0ϕ.’

In contrast, an observer using an adaptive measurement protocol can easily have zero uncertainty in the measurement outcomes once synchronized. For example, Figure 15 shows the recurrent states for a DQMP that swaps between two different measurements—$M_{01}$ and $M_{\phi }$—depending on the internal state of the source. The sequence of measurement outcomes observed is deterministic, and the recurrent measured process is a period-5 process with word ‘00ϕ0ϕ’ and $h_{\mu }^{Y_{r}}=0$.

### 5.5. Synchronizing with PVMs

The |0〉-|+〉 Quantum Golden Mean process has multiple synchronizing observations; see the generator in Figure 6. Measuring with $M_{01}$ and seeing a ‘1’ synchronizes the observer to internal state *B*; measuring with $M_{\pm }$ and seeing a ‘−’ synchronizes the observer to internal state *A*. Let us consider the mixed states produced by these two repeated PVMs in turn, starting with $M_{01}$.

**Figure 16:**
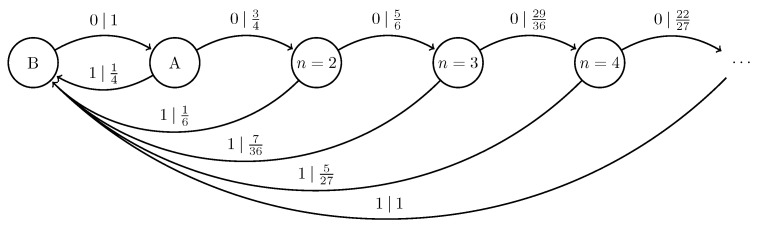
Mixed-state presentation for the |0〉-|+〉 Quantum Golden Mean process measured with $M_{01}$. *n* refers to the number of consecutive ‘0’s since the most recent ‘1’.

Figure 16 shows the mixed states for the measured process obtained by repeatedly applying $M_{01}$. Observing synchronizing measurement y=‘1’ means that the source just transitioned to state *B* while emitting a |+〉 qubit, i.e., H[η(‘1’)]=0. Additionally, the source in state *B* can only transition to state *A* while emitting a |0〉 qubit, so the observer will see a ‘0’, and H[η(‘10’)]=0. However, once the source is in state *A*, an observer easily desynchronizes from the source if they observe another ‘0’, as this is consistent with either of the two transitions to the source. As an observer sees more ‘0’s, they transition to mixed states further to the right of Figure 16. To summarize, measuring with $M_{01}$ makes use of synchronizing observations ‘1’ and ‘10’, but its MSP has a countably infinite number of recurrent states corresponding to sequences of *n* ‘0’s.

This measured process is an infinite-state classical renewal process whose information properties can be estimated using methods from Ref. [51]. After seeing *n* ‘0’s in a row, the next observation will be ‘1’ with the following probability:

$$\begin{array}{cc} \mathrm{Pr}(‘1’|{‘0’}^{n}) & =\frac{1}{4}*\mathrm{Pr}\left(A|{‘0’}^{n}\right)\\ \; & =\frac{3}{16}\left(1-{\left(-\frac{1}{3}\right)}^{n}\right)\;. \end{array}$$

We find $h_{\mu }^{Y}\approx 0.60$ bits per symbol, $E^{Y}\approx 0.053$ bits, and a single-symbol entropy of H[1]≈0.65 bits. $E^{Y}$’s small value and the fact that $h_{\mu }^{Y}$ is not significantly lower than H[l=1] indicate that the infinite-state renewal process presentation provides only a small predictive advantage over using a biased coin with Pr(‘0’)=5/6. This is further evidenced by how $\mathrm{Pr}\left(B|{‘0’}^{n}\right)=\frac{1}{4}\left(1-{\left(-1/3\right)}^{n-1}\right)$ converges exponentially quickly to its asymptotic value of $\mathrm{Pr}(B|{‘0’}^{n})=1/4$.

**Figure 17:**
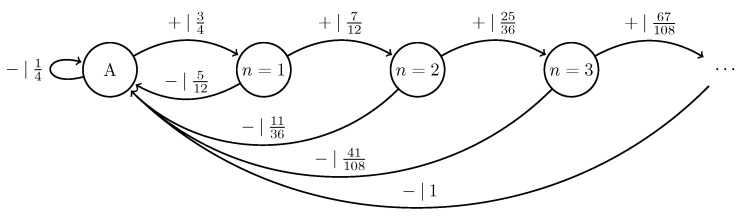
Mixed-state presentation for the |0〉-|+〉 Quantum Golden Mean process measured with $M_{\pm }$. *n* refers to the number of consecutive ‘+’s since the most recent ‘−’.

Measuring with $M_{\pm }$, symbol ‘−’ is a synchronizing observation and indicates the source is in state *A*. The recurrent mixed states for this observed process are shown in Figure 17, and they also form a classical renewal process. Observing *n* ‘+’s since the last ‘−’, the probability that the generator is in state *A* is $\mathrm{Pr}\left(A|{‘+’}^{n}\right)=\frac{3}{5}\left(1-{\left(-2/3\right)}^{n+1}\right)$. The measured process obtained with $M_{\pm }$ has $h_{\mu }^{Y}\approx 0.90$ bits per symbol, $E^{Y}\approx 0.020$ bits, and H[l=1]≈0.91 bits. Again, the infinite-state MSP provides only a small predictive advantage over a biased coin with Pr(‘+’)=2/3. Also, $\mathrm{Pr}(A|{‘+’}^{n})$ converges exponentially quickly to $\mathrm{Pr}(A|{‘+’}^{n})=3/5$.

**Figure 18:**
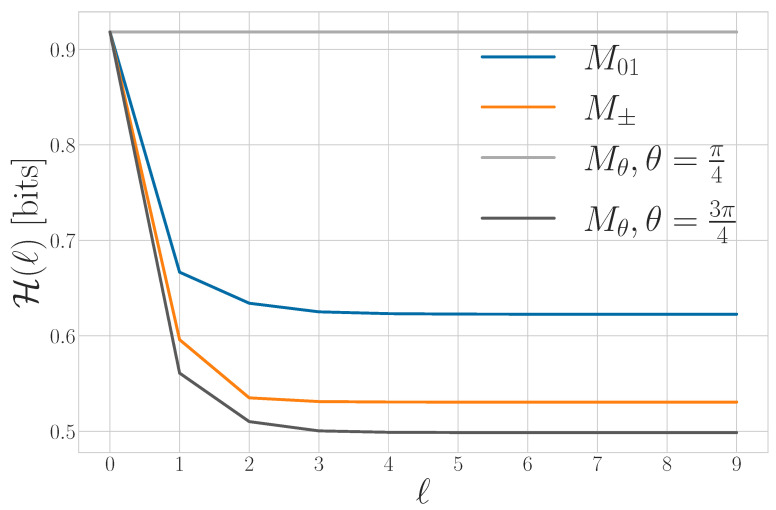
Average state uncertainty H(l) for the |0〉-|+〉 Quantum Golden Mean generator after *ℓ* measurements.

Applying either of these two fixed-basis measurements gives an average state uncertainty that decreases monotonically with *ℓ*, as shown in Figure 18. Since this source is not quantum unifilar, an observer repeatedly synchronizes and desynchronizes while measuring this process regardless of basis, and H(l) does not approach 0 as l→∞. We find that $C_{\infty }\left(M_{01}\right)\approx 0.62$ bits and $C_{\infty }\left(M_{\pm }\right)\approx 0.54$ bits. Measuring with $M_{\pm }$ not only results in less state uncertainty asymptotically but also results in less average uncertainty for all values of *ℓ*.

Figure 18 also displays the average state uncertainty for two other relevant PVMs: $M_{\theta }$ for θ=π/4 and θ=3π/4. Recall that $M_{\theta }$ is the PVM consisting of projectors onto orthogonal states |ψ(θ)〉 and |ψ(θ+π)〉. For comparison, $M_{01}$ corresponds to θ=0, and $M_{\pm }$ corresponds to θ=π/2.

**Figure 19:**
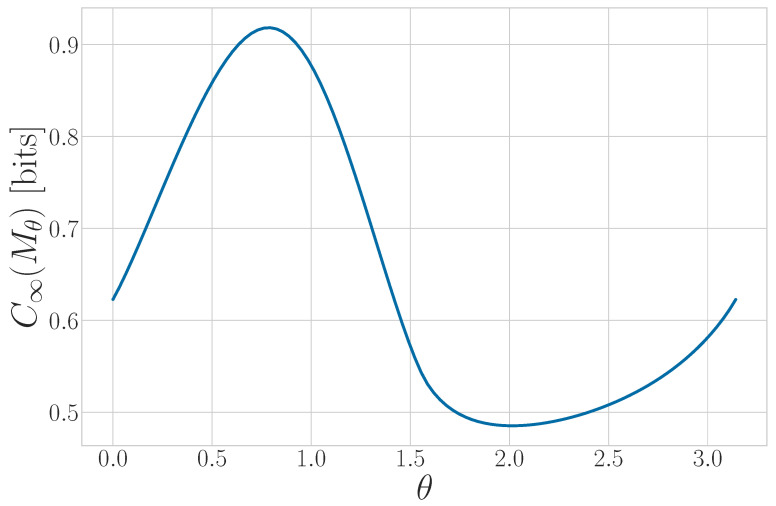
Asymptotic state uncertainty when applying the PVM $M_{\theta }$ to the |0〉-|+〉 Quantum Golden Mean.

For θ=π/4, the symbol states |0〉 and |+〉 give the observer the exact same distribution of measurement outcomes, and the observer cannot gain any information about the state of the source beyond the stationary state distribution. This can also been seen as the maximum of the asymptotic state uncertainty in Figure 19.

For θ=3π/4—and for the majority of values of θ—$M_{\theta }$ has no synchronizing observations. Despite this, Figure 18 demonstrates that $H(l|M_{\theta =3\pi /4})$ is lower for all *ℓ* than both bases that can exactly synchronize.

A measured process for generic θ does not have the renewal process structure of Figure 16 and Figure 17. Instead, in the absence of synchronizing observations, the number of mixed states generically grows exponentially with *ℓ*. If we approximate the MSP using length-*ℓ* observations, then there will be $|Y^{l}|$ mixed states—one for each possible sequence of measurements—each corresponding to a different distribution over the source’s internal states. These infinite-state MSPs can be characterized by their statistical complexity dimension [52].

Despite the explosive complexity, many of these PVMs give lower average state uncertainties than $M_{01}$ and $M_{\pm }$. θ=3π/4 is a representative example. The asymptotic state uncertainty when applying $M_{\theta }$ is shown in Figure 19. Note that the maximum value of $C_{\infty }\left(M_{\theta }\right)=H\left[\pi \right]$ occurs when θ=π/4, as discussed. The minimum asymptotic state uncertainty for this set of PVMs is $C_{\infty }\approx 0.49$ bits, which occurs for θ≈2.01. An observer may choose to use a basis with an exponential set of mixed states to lower their uncertainty in the source’s internal state at the cost of having to track the probability of a larger set of mixed states.

### 5.6. Maintaining Synchrony with Adaptive Measurement

Adaptive measurement protocols are also capable of maintaining synchronization when no fixed-basis measurement can. To appreciate this, consider Figure 11’s unifilar qubit source.

For 0<p<1, there are no synchronizing observations; however, almost any measurement reduces the average state uncertainty below H[π]. Additionally, if an observer comes to know the source’s state by other means (perhaps the source is initialized in state *A* at time t=0), then they are able to maintain synchronization with a simple adaptive measurement protocol $M_{adaptive}$.

This protocol, defined here only for recurrent states, is as follows. If the source is in state *A*, apply measurement $M_{01}$, and if the source is in state *B*, apply measurement $M_{\pm }$. It is only possible to define it this simply and maintain synchronization because the source is quantum unifilar.

**Figure 20:**
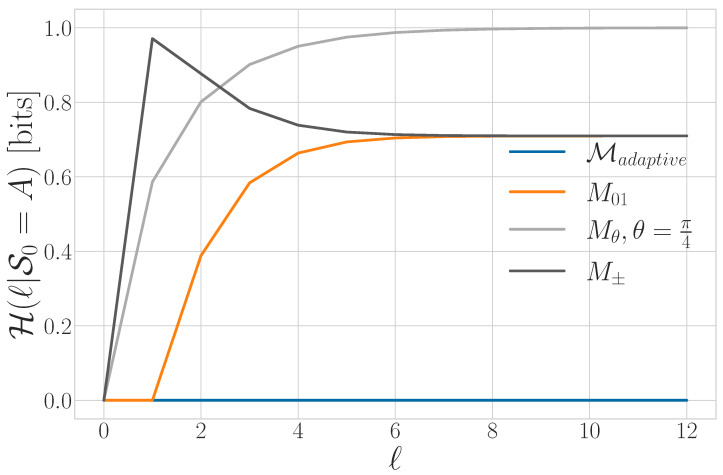
Average state uncertainty H(l) for the unifilar qubit source (p=0.6) initialized in state *A*. Only the adaptive measurement protocol (described in the text) is able to maintain synchronization.

Figure 20 shows the behavior of H(l) as the observer desynchronizes from the source initialized in state *A*. For each repeated PVM measurement, it eventually reaches an asymptotic value, though H(l) may be nonmonotonic.

### 5.7. Synchronizing to a Qutrit Source

A qubit source (d=2) presents limited opportunities for unambiguous state discrimination and, hence, for synchronization. Qutrits allow for more general behavior and have been suggested for applications in quantum communication networks where one state is reserved specifically for synchronization [53].

Let us apply this logic to the unifilar qutrit source in Figure 13. Synchronizing observation sequences for this process are ‘2’, ‘1+’, and ‘1−’ that definitively place the source in states *A*, *B*, and *C*, respectively. Once synchronized, an observer may remain synchronized by measuring with $M_{012}=\left\{|0\rangle \langle 0|,|1\rangle \langle 1|,|2\rangle \langle 2|\right\}$ when in state *A*, $M_{\pm 2}=\left\{|+\rangle \langle +|,|-\rangle \langle -|,|2\rangle \langle 2|\right\}$ when in state *B*, and either of the above when in state *C*.

**Figure 21:**
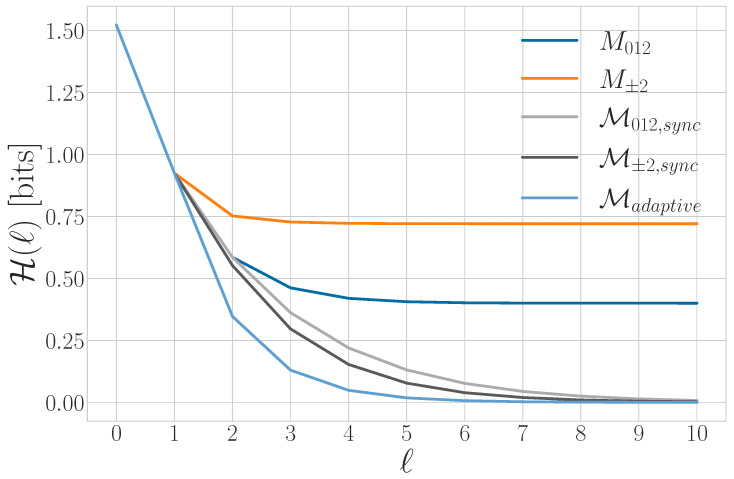
Average state uncertainty H(l) while measuring the process generated by the unifilar qutrit source. $M_{012}$ and $M_{\pm 2}$ are fixed-basis measurements. $M_{012,sync}$ and $M_{\pm 2,sync}$ measure in a fixed basis until they observe a 2 and stay permanently synchronized afterwards. $M_{adaptive}$ refers to the protocol in Figure 22.

**Figure 22:**
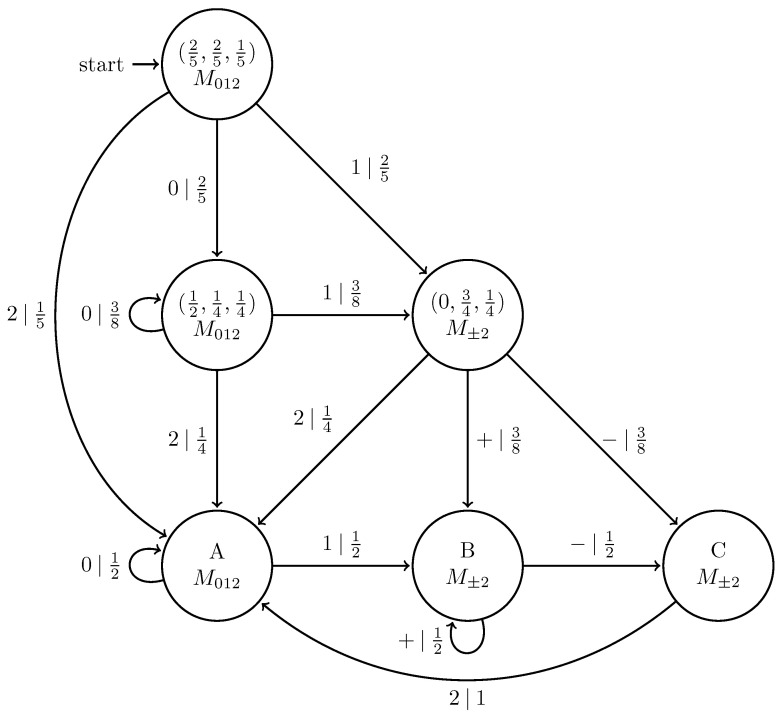
Adaptive measurement protocol defined for the qutrit process generator in Figure 13. The three transient mixed states are labeled with the internal source states probabilities $\left(p_{A},p_{B},p_{C}\right)$, and the three recurrent states correspond exactly to those states. This adaptive protocol permanently synchronizes to the source, since the source is quantum unifilar. Transitions with zero probability are omitted.

We explored synchronizing to this source with five different measurement protocols and compare them in Figure 21. The first two are repeated PVMs in the $M_{012}$ basis and the $M_{\pm 2}$ basis. The other three are adaptive measurement protocols that share a recurrent dynamic but have different transient states. Consider measuring in the $M_{012}$ ($M_{\pm 2}$) basis until observing a ‘2’ and therefore synchronizing to source state *A*. Then, use $M_{012}$ when the source is in state *A* and $M_{\pm 2}$ when the source is in state *B* or *C*. We refer to this protocol as $M_{012,sync}$ ($M_{\pm 2,sync}$). The fifth protocol $M_{adaptive}$ is defined by the DQMP in Figure 22 that uses an adaptive protocol over three transient mixed states in addition to the protocol just defined for the recurrent states.

The average state uncertainties for an observer implementing these five measurement protocols are shown in Figure 21. The fixed-basis measurements do not lead to persistent synchronization and have a nonzero asymptotic state uncertainty ($C_{\infty }\left(M_{012}\right)\approx 0.40$ bits and $C_{\infty }\left(M_{\pm 2}\right)\approx 0.72$ bits. If one measures in a fixed basis until synchronizing (by observing a ‘2’) and then takes advantage of the quantum unifilarity of the source to stay synchronized, then the asymptotic state uncertainty vanishes. This is the case for $M_{012,sync}$ and $M_{\pm 2,sync}$, which have synchronization information values of $S(M_{012,sync})\approx 3.91$ bits and $S(M_{012,sync})\approx 3.60$ bits. The extra complexity of the measurement protocol $M_{adaptive}$ admits additional synchronizing words (‘1+’ and ‘1−’) and lower synchronization information ($S(M_{adaptive})\approx 3.00$ bits) than the simpler strategy of waiting to see a ‘2’. Note that these three synchronization information values are all greater than our estimate of the quantum transient information for this process ($T_{q}\approx 2.16$ bits × symbols). These values values of S(M) were estimated using l=10.

### 5.8. Discussion

This section has detailed the process of synchronizing to a known qudit source. In contrast to sources of classical random variables, an observer may attempt to synchronize to a qudit source using a variety of measurement protocols. We can compare different protocols with two informational quantities introduced above: the asymptotic state uncertainty $C_{\infty }\left(M\right)$ and the synchronization information S(M).

For sources that are not quantum unifilar, no protocol can remain synchronized to the source. Nevertheless, for different measurement protocols $M_{0}$ and $M_{1}$, we can compare $C_{\infty }\left(M_{0}\right)$ and $C_{\infty }\left(M_{1}\right)$. The protocol with a lower value is better at the task of synchronization in the sense that an observer’s uncertainty in the source’s internal state will be lower on average.

This leads to a natural question: What is the best measurement protocol for synchronizing to a source? To answer it, we introduce a new protocol-independent property of a qudit process, the *minimal asymptotic state uncertainty*:

$$\begin{array}{c} C_{min}=\min_{M}C_{\infty }\left(M\right)\;, \end{array}$$

where the minimum is taken over all possible measurement protocols defined via DQMP. In practice, determining $C_{min}$ for a quantum process requires a proof that no measurement protocol can achieve a lower value. Fully exploring the space of measurement protocols is beyond the present scope. Nevertheless, we introduced candidates for $C_{min}$ in the above examples and found the minimal asymptotic state uncertainty for a restricted class of repeated PVMs numerically; recall Figure 19.

For sources that are quantum unifilar, we explored several protocols that are capable of persistent synchronization. For such processes, $C_{min}=0$ bits. We can compare different synchronizing measurement protocols $M_{0}$ and $M_{1}$ through their synchronization information values $S(M_{0})$ and $S(M_{1})$. A lower value means that the observer experiences less state uncertainty while synchronizing. What is the minimal amount of state uncertainty an observer can experience? We define the *minimal synchronization information* for a quantum process as

$$\begin{array}{c} S_{min}=\min_{M}S\left(M\right)\;. \end{array}$$

This minimum is also taken over all DQMPs, establishing that a given protocol is minimal and nontrivial.

One open question prompted by this work is ‘Is it always possible to synchronize to a source that is quantum unifilar?’ Equivalently, ‘Does $C_{min}=0$ for all processes generated by quantum unifilar sources?’ Answering these questions will also require a greater understanding of the space of DQMPs. Progress may involve proving the existence or nonexistence of a protocol that is able to synchronize to the unifilar qubit source in Figure 11.

Finally, we recount several reasons why synchronization is an important task not only for determining a source’s internal state but also for improving predictions of future measurement outcomes. Generally, HMCQSs have inherent stochasticity. Periodic sources are an exception. Through synchronization, we may substantially reduce the uncertainty in measurement outcomes. In the extreme case where a source’s internal state has only one possible transition, this uncertainty vanishes, and we can measure the next qudit with a PVM which has a deterministic outcome.

For example, if we know a |0〉-|+〉 Quantum Golden Mean generator is in state *B*, the next qudit is |0〉, and we will always see ‘0’ if we apply the measurement $M_{01}$. Thus, we consider synchronization as a form of dynamical inference where an observer uses knowledge of both a source’s internal structure and a sequence of measurement outcomes to inform a more accurate prediction of future measurement outcomes. Each mixed state corresponds to a different prediction.

## 6. Quantum Process System Identification

Synchronization is a task that is performed when an observer has an accurate model (here, a HMCQS) of the quantum information source. The next natural question is the following: How does an observer create an accurate model of an *unknown* quantum information source? This section begins to answer this question. It starts by briefly reviewing how one infers a classical information source from data. It then discusses how to identify the state of a single qudit with quantum state tomography. By combining these two ideas we arrive at an inference method for stationary qudit sequences.

### 6.1. Classical System Identification

An observer of an unknown stationary classical process is limited to use only the available data—distributions of words over symbol alphabet X—to infer the source’s structure. We denote the distribution of length-*ℓ* words as $P\left(\ell \right)=\left\{Pr\left(w\right)\forall w\in X^{\ell }\right\}$.

For l=1, we obtain a distribution over symbols in X. With P(1), we can reconstruct a memoryless (i.i.d.) model of the source with one internal state, and each Pr(x∈X) is obtained directly from P(1).

For l=2, we use P(2) to create conditional probability distributions for the next symbol conditioned on the previous one; i.e., $\mathrm{Pr}(x_{1}|x_{0})$, for all $x_{0}$, where $x_{1}\in X$. From these conditional distributions, we may construct a Markov approximation of the source with |X| states, one corresponding to each symbol. The conditional distributions set the transition matrices for Markov approximation of the source, i.e., $\mathrm{Pr}\left(x_{1}|x_{0}\right)=T_{x_{0},x_{1}}$.

Similarly, for l>2, we obtain a length-*ℓ* HMM approximation of the source by conditioning on length l−1 words, each of which corresponds to a different internal state. In this simplified picture, the number of internal states of the model grows exponentially with *ℓ*, as the number of possible words of length *ℓ* is ${\left|X\right|}^{l}$. This leads to numerical problems when inferring processes with correlations over long periods of time. That said, there are methods for both combining states that reflect identical predictions of future symbols and for performing inference over machine topologies with a certain number of states—known as Bayesian Structural Inference [54]—to determine the most likely ε-machine for the source.

### 6.2. Tomography of a Qudit

We cannot directly apply the procedure for inferring classical source dynamics from word distributions to qudit processes, since observations depend on the measurement basis/protocol one uses. To fully characterize a stationary quantum source, we must instead take many measurements in different bases to reconstruct the qudit density matrices. This task is known as *quantum state tomography*. We begin by inferring an individual qudit state $\rho_{0}$ before introducing a general method for quantum system identification using separable sequences of qudits.

Tomographic reconstruction of a single unknown qudit density matrix $\rho_{0}$ through measurement is challenging for two main reasons:

1. $\rho_{0}$’s complete description requires a number of parameters that scales exponentially with the state’s Hilbert space dimension.
2. Quantum measurement is probabilistic, so one must prepare and measure many copies of $\rho_{0}$ to estimate a single parameter.

Specific combinations of measurements are particularly useful for this task. For example, $\rho_{0}$ can be inferred by measuring with a set of mutually unbiased bases (MUBs) [3] or a single informationally complete POVM (IC-POVM) [4]. For a qubit, one possible MUB consists of the x, y, and z bases. By measuring many copies of the qubit in each of these three bases, one obtains three probabilities—Pr(+x), Pr(+y), and Pr(+z)—that uniquely determine the density matrix $\rho_{0}$. We do not discuss the necessary number of measurements to determine these parameters to within a desired tolerance for general qudit tomography, a question which is well studied [33,55].

An example of an IC-POVM for a qubit consists of the projectors onto states:

(52) $$\begin{array}{cc} |\phi_{1}\rangle & =|0\rangle ,\\ |\phi_{2}\rangle & =\frac{1}{\sqrt{3}}|0\rangle +\sqrt{\frac{2}{3}}|1\rangle ,\\ |\phi_{3}\rangle & =\frac{1}{\sqrt{3}}|0\rangle +\sqrt{\frac{2}{3}}e^{i2\pi /3}|1\rangle ,\;\;\mathrm{and}\\ |\phi_{4}\rangle & =\frac{1}{\sqrt{3}}|0\rangle +\sqrt{\frac{2}{3}}e^{i4\pi /3}|1\rangle \;. \end{array}$$

This is also a symmetric IC-POVM, or SIC-POVM, because any combination of two projectors has the same inner product.

By measuring many identical copies of $\rho_{0}$ with the same IC-POVM, one obtains a probability distribution over the $d^{2}$ possible measurement outcomes. This provides $d^{2}-1$ parameters (due to normalization) that uniquely determine the density matrix $\rho_{0}$.

The existence and properties of SIC-POVMs in higher-dimensional Hilbert spaces is an active area of research [56].

### 6.3. Tomography of a Qudit Process

Now that we know how to estimate the density matrix for an individual qudit, we can begin analyzing length-*ℓ* density matrices $\rho_{0:l}$. We will measure each qudit in turn rather than perform a joint measurement over the entire length-*ℓ* block of qudits. This is a luxury afforded to us because we are focusing on separable qudit sequences—the generic case of entangled qudit processes requires measuring in nonlocal bases.

For l=1, we reconstruct $\rho_{0}$ as described above and obtain a memoryless (i.i.d.) estimate of the source that emits qudit $\rho_{0}$ at every time step, following Equation (4). Unless $\rho_{0}$ is a pure state, there are many single-state HMCQSs that generate this process, since many different pure-state ensembles correspond to the same density matrix. A unique memoryless model of the source may be obtained by diagonalizing $\rho_{0}$ and having the source emit each pure eigenstate $|\psi_{i}\rangle$ with probability equal to the corresponding eigenvalue $\lambda_{i}$.

For l=2, we must reconstruct the two-qudit density matrix $\rho_{0:2}$. (Recall that our indexing is left-inclusive and right-exclusive; therefore, $\rho_{0:2}$ is the joint state of the qudits for t=0,1). Due to stationarity, $\rho_{0:2}$ (the joint state of the two qudits) must be consistent with the one-qudit marginals, i.e., $tr_{0}\left(\rho_{0:2}\right)=\rho_{1}=\rho_{0}=tr_{1}\left(\rho_{0:2}\right)$.

We will describe the iterative procedure for reconstructing $\rho_{0:l}$ for *qubits* in detail. When d=2, $\rho_{0:2}$ has 15 real parameters that must be determined via tomography. Tomography on the one-qubit marginals determines three parameters, and the condition of stationarity fixes three more. The state can be reconstructed fully by considering combinations of the set of mutually unbiased measurements. For two qubits, this means the 16 combinations of Pauli matrices ($\sigma_{I_{0}}⊗\sigma_{x_{1}}$, $\sigma_{x_{0}}⊗\sigma_{x_{1}}$, $\sigma_{x_{0}}⊗\sigma_{y_{1}}$, and so on) [57]. To fully characterize $\rho_{0:2}$, nine of these values must be determined—those not involving the identity operators $\sigma_{I_{0}}$ and $\sigma_{I_{1}}$ which are fixed by the one-qubit marginals. For d>2, this procedure can be modified by using a set of mutually unbiased bases in that higher-dimensional Hilbert space.

After determining $\rho_{0:2}$, one can continue on to determine $\rho_{0:3}$ (63 real parameters for qubits). Many of these parameters are fixed by the previous tomography on the one-qubit marginals (3 parameters), the two-qubit marginals (15 parameters), and their stationarity conditions (6 and 15 parameters, respectively). Combinations of three one-site Pauli matrices are sufficient for full reconstruction. One may continue this procedure for larger *ℓ*, typically until the number of measurements becomes experimentally infeasible.

### 6.4. Cost of I.I.D.

Quantum information sources are often assumed to be i.i.d. [6,33]. If an observer performing quantum state tomography assumes that an unknown quantum information source is i.i.d., then they will not go beyond determining the one-qudit marginal $\rho_{0}$. If the qudits are instead correlated, this will lead to an overestimate of the source’s randomness. They will erroneously conclude that each qudit is in state $\rho_{0}$ and that the entropy rate is $S(\rho_{0})$ bits per time step. The latter overestimates the true entropy rate by a factor of S(1)−s. An observer can obtain better estimates for the entropy rate and other informational quantities by following the above procedure and tomographically reconstructing blocks of qudits of length *ℓ*.

To demonstrate the degree to which this assumption may mislead an observer, consider the process generated by a nonunifilar qubit source in Figure 12. Assume that the source begins in its stationary state distribution: $\left(p_{A}=1/2,p_{B}=1/2\right)$. For any *p* such that 0≤p<1, $\rho_{0}$ is the maximally mixed state, and an observer assuming an i.i.d. process estimates that s=1 bit per time step. This is only an accurate description of the source for p=1/2, whereas, for many values of *p*, this source has significant correlations between subsequent qubits.

Let us take a closer look at the extreme values of *p*. For p=0, the process is period-2 with s=0 bits per time step and $E_{q}=1$ bit. This pattern can be easily detected by measuring in the {|0〉,|1〉} basis, where a measurement of 0 (1) immediately synchronizes an observer to state *B* (*A*). As p→1, the two source states become increasingly disconnected, s→0, and $E_{q}\to 1$. An observer measuring in the {|+〉,|−〉} basis observes a + (−) and is likely to measure another + (−). This source’s rich and varied behavior at different values of *p* will go entirely unappreciated when considering only the one-qubit density matrix, $\rho_{0}$.

### 6.5. Finite-Length Estimation of Information Properties

We just saw an example of how, if an observer assumes a source is i.i.d., they will generally underestimate the structure and correlation of the quantum process and will overestimate its entropy rate. The same is true if they only perform tomography on blocks of qudits up to finite length *ℓ*. Consider now an experimenter who tomographically reconstructs the density matrix $\rho_{0:l}$ and then assumes there are no additional (longer-range) correlations within the qudit process.

Their estimates for the quantum entropy rate, quantum excess entropy, and quantum transient information are given by Equations (38), (45), and (47), respectively.

**Figure 23:**
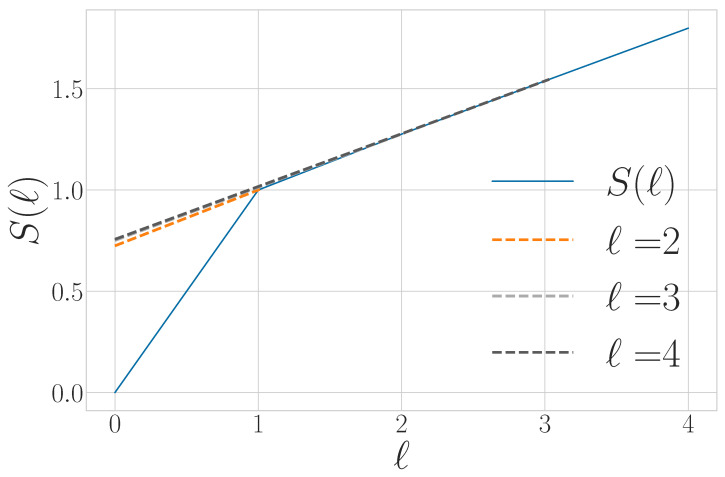
Block entropy and length-*ℓ* estimates for information properties of the process generated by the nonunifilar qubit source in Figure 12, with p=0.05. The slope and *y*-intercept of the linear estimates are *s* and $E_{q}$, respectively. Note that the estimates do not improve significantly for l>2, indicating that the two-qubit correlations are most significant for determining information properties of the process.

**Figure 24:**
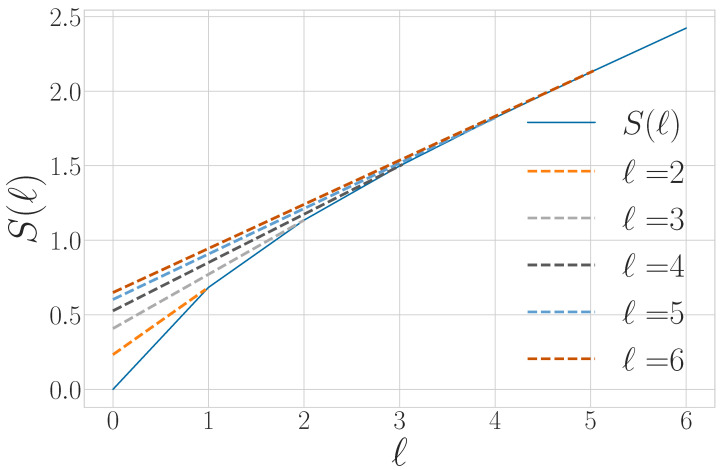
Block entropy and length-*ℓ* estimates for information properties of the process generated by the unifilar qubit source in Figure 11, with p=0.05. The slope and *y*-intercept of the linear estimates are *s* and $E_{q}$, respectively. Note that the estimates improve steadily for larger *ℓ*, indicating long-range correlations.

The difference between the length-*ℓ* estimate of an information property and its true value depends on the process’s internal structure and quantum alphabet. The estimates for the nonunifilar and unifilar qubit sources in Figure 23 and Figure 24 represent two extremes in this respect. As previously discussed, the single-qubit density matrix for the nonunifilar qubit source is the maximally mixed state. If an observer instead reconstructs $\rho_{0:2}$, they significantly improve their estimate of *s*, $E_{q}$, and $T_{q}$. However, for l>2, these estimates do not improve dramatically. There is always a tradeoff between the number of experiments necessary to reconstruct the process tomographically and the accuracy of the estimates obtained from that reconstruction. For this source, l=2 strikes a balance between those two resources.

In contrast, for the unifilar qubit source, the estimates of *s*, $E_{q}$, and $T_{q}$ improve steadily as *ℓ* increases. Longer-range correlations are captured by increasing the length of the reconstructed density matrices $\rho_{0:l}$. When observing this source, it is likely worth finding $\rho_{0:l}$ for the largest *ℓ* that is experimentally feasible.

When faced with an unknown quantum source, how does one pick an appropriate value of *ℓ*? One strategy is to increase *ℓ* until the correction made to the relevant information quantities by going to l+1 is below some threshold. Stationarity (and the resulting concavity of the quantum block entropy) ensures that future corrections will also be below that threshold.

### 6.6. Tomography with a Known Quantum Alphabet

**Figure 25:**
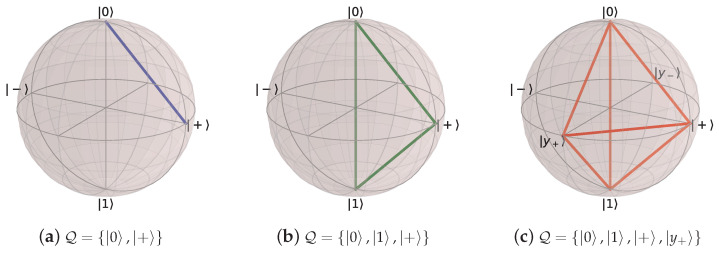
The Bloch sphere representation of the boundary of the set of possible length-1 density matrices ($\rho_{0}$) for the given Q. (**a**) The set of valid states is a line segment. An observer only needs to determine one parameter. (**b**) The set of valid states is the interior of a triangle. An observer must determine two parameters. (**c**) The set of valid states is the interior of a tetrahedron. An observer must determine three parameters, and the decomposition into an ensemble of basis states is not unique. Here, $|y_{+}\rangle =\frac{1}{\sqrt{2}}\left(|0\rangle -i|1\rangle \right)$.

If an observer has additional knowledge of what possible pure states an HMCQS may emit (i.e., the quantum alphabet Q), they can leverage this knowledge to simplify the task of system identification by inferring the word probabilities of the underlying classical process $\overset{↔}{X}$ rather than performing full tomographic reconstruction. For qubits, we can represent this simplification geometrically via the Bloch sphere; see Figure 25. Each point on the surface of the Bloch sphere represents a pure qubit state in $H^{2}$—such as |0〉 and |1〉 on the poles of the z axis—and each interior point represents a possible qubit density matrix. The following assumes that each element of Q is unique.

#### 6.6.1. l=1

The length-1 density matrix $\rho_{0}$ must satisfy the equation

$$\begin{array}{c} \rho_{0}=\sum_{|\psi_{x}\rangle \in Q}\mathrm{Pr}\left(|\psi_{x}\rangle \right)|\psi_{x}\rangle \langle \psi_{x}|\;. \end{array}$$

After finding $\rho_{0}$ via tomography, one may rearrange this equation to infer the length-1 word distributions of $\overset{↔}{X}$, given that $\mathrm{Pr}(X=x)=\mathrm{Pr}(|\psi_{x}\rangle )$. The feasibility of this task depends on the relationship between |Q| and *d*. For the qubit case (d=2), one can uniquely infer $\mathrm{Pr}(X_{0})$ if |Q|≤3, assuming no degenerate states in Q.

When Q is known, it may not be necessary to fully reconstruct $\rho_{0}$. We first present several simple examples before giving a general algorithm for finding $\rho_{0:l}$ without performing full-state tomography on $\rho_{0:l}$.

For d=2 and |Q|=2, the possible values of $\rho_{0}$ are restricted to a chord within the Bloch sphere defined by $\rho_{0}=p|\psi_{0}\rangle \langle \psi_{0}|+\left(1-p\right)|\psi_{1}\rangle \langle \psi_{1}|$ with 0<p<1. One needs only to determine the parameter *p* rather than reconstruct $\rho_{0}$ in its entirety. An observer can also pick a uniquely informative measurement to determine *p*. The optimal PVM for doing so is one whose antipodal projectors can be connected with the diameter of the Bloch sphere that runs parallel to the line of possible values of $\rho_{0}$. For example, if Q={|0〉,|+〉}, then the set of possible density matrices lies on the line segment in Figure 25a. The best PVM is then $M_{\theta }$ with $\theta =\frac{3\pi }{4}$ and measurement outcomes $y_{0}$ (corresponding to a projection on pure-state $|\psi (\theta =\frac{3\pi }{4})\rangle$) and $y_{1}$ (corresponding to the orthogonal projector). In this case, it can easily be shown that $p=\frac{\sqrt{2}+1}{2}-\sqrt{2}\mathrm{Pr}\left(y_{0}|\rho_{0}\right)$ and that $\cos^{2}\left(\frac{3\pi }{8}\right)\leq \mathrm{Pr}\left(y_{0}|\rho_{0}\right)\leq \sin^{2}\left(\frac{3\pi }{8}\right)$. $M_{01}$ and $M_{\pm }$ would also be able to determine *p* but require more samples to determine *p* to within some desired tolerance.

For d=2 and |Q|=3, the possible values of $\rho_{0}$ are confined to a simplex in the Bloch sphere defined by $\rho_{0}=p_{0}|\psi_{0}\rangle \langle \psi_{0}|+p_{1}|\psi_{1}\rangle \langle \psi_{1}|+\left(1-p_{0}-p_{1}\right)|\psi_{2}\rangle \langle \psi_{2}|$, with $0<p_{0},p_{1}<1$ and $p_{0}+p_{1}<1$. One may determine the parameters $p_{0}$ and $p_{1}$ rather than the three parameters usually required to characterize a qubit mixed state. There are two simple choices of measurements to do so: using a IC-POVM or using two different PVMs.

For the first case, consider measuring $\rho_{0}$ with a SIC-POVM with elements $E_{y}=\frac{1}{2}|\phi_{y}\rangle \langle \phi_{y}|$, with each $|\phi_{y}\rangle$ described by Equation (52). The probability of observing measurement *y* can be written as

$$\begin{array}{cc} \mathrm{Pr}(y|\rho_{0}) & =\sum_{x}\mathrm{Pr}\left(|\psi_{x}\rangle \right)\mathrm{Pr}\left(y||\psi_{x}\rangle \right)\\ \; & =\sum_{x}\frac{p_{x}}{2}{\left|\langle \psi_{x}|\phi_{y}\rangle \right|}^{2}\;. \end{array}$$

One can rearrange the system of 4 equations (one for each POVM element) to obtain a unique set of $p_{x}$’s.

Alternatively, one uses two PVMs whose projectors can be connected by (ideally orthogonal) diameters of the Bloch sphere that are parallel to the simplex of possible $\rho_{0}$ values. This will yield two parameters that uniquely determine a point on the $\rho_{0}$ simplex. An example with Q={|0〉,|1〉,|+〉} is shown in Figure 25b, for which the possible values of $\rho_{0}$ are confined to the interior of a triangle in the Bloch sphere. One can determine $p_{0}$ and $p_{1}$ by measuring with orthogonal PVMs $M_{01}$ and $M_{\pm }$ (among many other combinations), in which case $\left(1-p_{0}-p_{1}\right)=2\mathrm{Pr}($‘+’$|\rho_{0},M_{\pm })-1$, and $\left(p_{0}-p_{1}\right)=2\mathrm{Pr}($‘0’$|\rho_{0},M_{01})-1$.

For d=2 and |Q|=4, the possible values of $\rho_{0}$ are confined to a tetrahedron in the Bloch sphere whose vertices are the elements of Q, and one cannot uniquely infer the classical symbol distribution from a fully reconstructed $\rho_{0}$. For example, if Q={|0〉,|1〉,|+〉,|−〉}, and $\rho_{0}$ is the maximally mixed state, this could correspond to any mixture of the form $\rho_{0}=p|0\rangle \langle 0|+p|1\rangle \langle 1|+\left(1-p\right)|+\rangle \langle +|+\left(1-p\right)|-\rangle \langle -|$ with 0<p<1. Thus, for d=2 and |Q|≥3, the tomographic advantage to knowing Q is reduced but not eliminated, as an observer can immediately exclude any value of $\rho_{0}$ that lies outside the convex polyhedron defined by the elements of Q. This is shown in Figure 25c, where the region of possible $\rho_{0}$ values is confined to less than $\frac{1}{4}$ of the volume of the Bloch sphere.

Similar simplifications apply for d=3 (qutrits) when Q is known. Full tomography of an arbitrary mixed qutrit state requires the determination of eight parameters, whereas determining the classical distribution given Q requires |Q|−1 parameters. This presents an advantage in general when |Q|≤8. We do not explicitly construct measurements that can realize this advantage, as a geometric understanding of mixed states over the eight-dimensional space $H^{3}$ is significantly more involved than the three-dimensional Bloch sphere describing mixed states over $H^{2}$.

For a generic qudit, the number of parameters required for full tomography is $d^{2}-1$. And so, we expect that knowledge of Q gives a clear tomographic advantage (fewer parameters must be determined) when $|Q|<d^{2}$.

We are now prepared to give a general protocol for |Q|=n and arbitrary *d*. We wish to recover the underlying distribution of alphabet states ($p_{x}=\mathrm{Pr}\left(|\psi_{x}\rangle \right)$, 0≤x<n) from measurement statistics alone. First, we construct a POVM with n+1 elements: $E_{y}=c_{y}|\psi_{x}\rangle \langle \psi_{x}|$ for each $|\psi_{x}\rangle$ in Q, and $E_{n}=I-\sum_{y=0}^{n-1}E_{y}$. Each $c_{y}$ is a parameter which can be varied to ensure that $E_{n}$ is positive semi-definite. By applying this POVM to $\rho_{0}$, we obtain a distribution ($\mathrm{Pr}(y|\rho_{0})$) over the n+1 possible measurements. The first *n* are related to our desired distribution by

$$\begin{array}{cc} \mathrm{Pr}(y|\rho_{0}) & =\sum_{x}p_{x}\mathrm{Pr}\left(y||\psi_{x}\rangle \right)\\ \; & =\sum_{x}p_{x}c_{y}{\left|\langle \psi_{x}|\psi_{y}\rangle \right|}^{2}\;, \end{array}$$

with one equation for each y<n. If a set of $p_{x}$’s is a solution to this system of linear equations, it is consistent with the observed measurements. The solution will be unique for $|Q|<d^{2}$.

#### 6.6.2. l=2

Knowing Q provides further advantage when considering the tomography of multiple qudits. The distribution over classical words of length *ℓ* has ${\left|Q\right|}^{l}-1$ parameters, whereas full tomography of *ℓ* qudits requires the determination of $d^{2l}-1$ parameters.

For l=2,

(53) $$\begin{array}{c} \begin{array}{c} \rho_{0:2}=\sum_{|\psi_{x_{0}}\rangle ,|\psi_{x_{1}}\rangle \in Q}\left[\mathrm{Pr}\left(|\psi_{x_{0}}\rangle ⊗|\psi_{x_{1}}\rangle \right)\left(|\psi_{x_{0}}\rangle ⊗|\psi_{x_{1}}\rangle \right)\left(\langle \psi_{x_{0}}|⊗\langle \psi_{x_{1}}|\right)\right]\;. \end{array} \end{array}$$

Once again, we consider the case of d=2 and |Q|=2 explicitly. There are four length-2 classical word probabilities, but there are three constraints imposed by (i) normalization, (ii) stationarity, and (iii) consistency with the one-qubit marginal. Thus, one only needs to determine a single parameter to reconstruct $\rho_{0:2}$.

Consider the task of reconstructing the length-2 density matrix produced by the |0〉-|+〉 Quantum Golden Mean generator in Figure 6 with the knowledge that Q={|0〉,|+〉}. One would first analyze the one-qubit density matrix to find that $\mathrm{Pr}\left(|0\rangle \right)=\frac{2}{3}$ and $\rho_{0}=\frac{2}{3}|0\rangle \langle 0|+\frac{1}{3}|+\rangle \langle +|$.

The word probability Pr(|00〉) is the only necessary additional information to find $\rho_{0:2}$. The following simple measurement protocol can determine Pr(|00〉): Measure two consecutive qubits with $M_{\pm }$. If the qubit is in state |+〉, one will never see outcome ‘−.’ If the qubit is in state |0〉, one will see outcome ‘−,’ with probability $\frac{1}{2}$. And so, Pr(|00〉)=4Pr(‘−−’$|\rho_{0:2},M_{\pm }⊗M_{\pm })$.

This procedure can be generalized to arbitrary two-element alphabets $Q=\{|\psi_{0}\rangle ,|\psi_{1}\rangle \}$. First, measure two consecutive qudits with a PVM $M_{\tilde{0}1}$, where one element is a projector onto $|\psi_{1}\rangle$ with outcome ‘1’ and the orthogonal projector corresponds to measurement outcome ‘$\tilde{0}$’. Second,

$$\begin{array}{cc} \mathrm{Pr}( & |\psi_{0}\rangle ⊗|\psi_{0}\rangle )\\ \; & ={\left(\mathrm{Pr}\left(\text{`}\tilde{0}\text{’}||\psi_{0}\rangle ,M_{\tilde{0}1}\right)\right)}^{-2}\mathrm{Pr}\left(\text{`}\tilde{0}\tilde{0}\text{’}|\rho_{0:2},M_{\tilde{0}1}⊗M_{\tilde{0}1}\right)\\ \; & ={\left(1-\left|\langle \psi_{0}|\psi_{1}\rangle \right|\right)}^{-2}\mathrm{Pr}\left(\text{`}\tilde{0}\tilde{0}\text{’}|\rho_{0:2},M_{\tilde{0}1}⊗M_{\tilde{0}1}\right)\;. \end{array}$$

This provides a clear advantage over the usual nine parameters necessary to reconstruct $\rho_{0:2}$, as it takes into account that the one-qubit marginals and stationarity each impose three constraints.

For d=2 and |Q|≥3, we must determine additional parameters of the underlying classical distribution over Q. We do so by repeatedly applying the SIC-POVM with elements $E_{y}=\frac{1}{2}|\phi_{y}\rangle \langle \phi_{y}|$, with each $|\phi_{y}\rangle$ described by Equation (52).

The probability of observing the length-2 measurement sequence $y_{0}y_{1}$ can be written as

$$\begin{array}{cc} \mathrm{Pr}(y_{0}y_{1}|\rho_{0:2}) & =\sum_{x_{0},x_{1}}p_{x_{0},x_{1}}\mathrm{Pr}\left(y_{0}y_{1}||\psi_{x_{0}}\rangle ⊗|\psi_{x_{1}}\rangle \right)\\ \; & =\sum_{x_{0},x_{1}}\frac{p_{x_{0},x_{1}}}{4}{\left|\langle \psi_{x_{0}}|\phi_{y_{0}}\rangle \right|}^{2}{\left|\langle \psi_{x_{1}}|\phi_{y_{1}}\rangle \right|}^{2}\;. \end{array}$$

There are ${\left|Q\right|}^{2}$$p_{x_{0},x_{1}}$s. If a set of $p_{x_{0},x_{1}}$s is a solution to this system of equations, it is consistent with the measurement statistics, and the solution will be unique for |Q|=3.

For |Q|=n and arbitrary *d*, we may construct a POVM from Q, as we did for l=1. It is then possible to infer a set of $p_{x_{0},x_{1}}$ that solves the resulting system of equations from measurement statistics. For length-2 words, the equations are

$$\begin{array}{cc} \mathrm{Pr}(y_{0}y_{1}|\rho_{0:2}) & =\sum_{i}p_{x_{0},x_{1}}\mathrm{Pr}\left(y_{0}y_{1}||\psi_{x_{0}}\rangle ⊗|\psi_{x_{1}}\rangle \right)\\ \; & =\sum_{x_{0},x_{1}}p_{x_{0},x_{1}}c_{y_{0}}c_{y_{1}}{\left|\langle \psi_{x_{0}}|\psi_{y_{0}}\rangle \right|}^{2}{\left|\langle \psi_{x_{1}}|\psi_{y_{1}}\rangle \right|}^{2}\;. \end{array}$$

There are $n^{2}$ equations, one for each length-2 measurement sequence corresponding to the POVM elements $E_{y_{0}}⊗E_{y_{1}}$. Calculating the other possible outcomes (corresponding to $E_{n}$) is redundant due to normalization.

#### 6.6.3. l≥3

Extending this analysis to a length-*ℓ* density matrix $\rho_{0:l}$ takes the form

$$\begin{array}{c} \rho_{0:\ell }=\sum_{|\psi_{w}\rangle \in Q^{\ell }}\left[\mathrm{Pr}(|\psi_{w}\rangle )|\psi_{w}\rangle \langle \psi_{w}|\right]\;, \end{array}$$

where each $|\psi_{w}\rangle$ has the form of Equation (5). One can determine the length-*ℓ* word distributions uniquely for the general case where ${\left|Q\right|}^{l}<d^{2l}$.

The various measurement strategies explored above for l=2 can be extended to arbitrary values of *ℓ*. We will explicitly describe two: using a SIC-POVM for d=2 and using a POVM constructed from Q for arbitrary *d*.

Consider repeatedly applying the SIC-POVM with elements $E_{y}=\frac{1}{2}|\phi_{y}\rangle \langle \phi_{y}|$, with each $|\phi_{y}\rangle$ described by Equation (52). Taking *ℓ* measurements, one observes a length-*ℓ* word $y_{0:l}$. Y is the four-element alphabet of measured symbols.

The probability of observing the length-*ℓ* measurement sequence $y_{0:l}$ can be written as

$$\begin{array}{cc} \mathrm{Pr}(y_{0:\ell }|\rho_{0:\ell }) & =\sum_{|\psi_{w}\rangle }\mathrm{Pr}\left(|\psi_{w}\rangle \right)\mathrm{Pr}\left(y_{0:\ell }||\psi_{w}\rangle \right)\\ \; & =\sum_{|\psi_{w}\rangle }\frac{\mathrm{Pr}(|\psi_{w}\rangle )}{2^{\ell }}{\left|\langle \psi_{w}|\phi_{y_{0:\ell }}\rangle \right|}^{2}\;, \end{array}$$

where $|\phi_{y_{0:l}}\rangle ={⨂}_{t=0}^{l}|\phi_{y_{t}}\rangle$, and the factor of $2^{l}$ comes from the POVM elements. Each of the $4^{l}$ sequences has a probability that can be estimated from measurement. This system of equations can be solved to find underlying length-*ℓ* word probabilities that are consistent with measurements. If |Q|≤3, this solution is unique.

We can also measure *ℓ* times with a POVM constructed directly from Q. In this case, the resulting equations are

$$\begin{array}{cc} \mathrm{Pr}(y_{0:\ell }|\rho_{0:\ell }) & =\sum_{|\psi_{w}\rangle }\mathrm{Pr}\left(|\psi_{w}\rangle \right)\mathrm{Pr}\left(y_{0:\ell }\right)\left||\psi_{w}\rangle \right)\\ \; & =\sum_{|\psi_{w}\rangle }p_{w}\left(\prod_{t=0}^{\ell -1}c_{y_{t}}\right){\left|\langle \psi_{w}|\psi_{y_{0:\ell }}\rangle \right|}^{2}\;. \end{array}$$

As before, one infers the ${\left|Q\right|}^{l}$ underlying word probabilities ($p_{w}$’s) uniquely in the case where $|Q|<d^{2}$.

### 6.7. Source Reconstruction

After observing a separable qudit process and finding the length-*ℓ* density matrix $\rho_{0:l}$, can one infer the HMCQS that generated it? The following reconstructs the source that generates the $\rho_{0:l}$ of an unknown process for different values of *ℓ*. Note that a source which generates $\rho_{0:l}$ may fail to generate $\rho_{0:l+1}$.

#### 6.7.1. l=1

After determining $\rho_{0}$, an observer may construct an i.i.d. approximation of the quantum information source. Given any decomposition of $\rho_{0}$ into pure states $|\psi_{x}\rangle$—as in Equation (3)—the corresponding HMCQS consists of one internal state $Q=\{|\psi_{x}\rangle \}$, and the single transition probability for each $|\psi_{x}\rangle$ is its corresponding probability $\mathrm{Pr}(|\psi_{x}\rangle )$ in the decomposition of $\rho_{0}$. A unique model may be obtained by taking $\rho_{0}$’s eigendecomposition. In this case, the model emits each eigenstate $|\psi_{i}\rangle$ with a probability equal to the corresponding eigenvalue: $\mathrm{Pr}\left(|\psi_{i}\rangle \right)=\lambda_{i}$.

#### 6.7.2. l=2

With $\rho_{0:2}$, an observer begins to model a source that generates correlations between qudits. Doing so requires finding a separable decomposition of $\rho_{0:2}$ of Equation (53)’s form. If Q is known, multiple procedures for finding such a separable decomposition have been introduced above. For a generic two-qudit density matrix, determining whether it is separable or entangled is generally NP-hard [58]. There are also many necessary and sufficient conditions for separability; for example, there is the Positive Partial Transpose (PPT) criterion [59].

The following assumes that the observer has a separable decomposition of $\rho_{0:2}$ into alphabet states $Q^{\prime }$ that may or may not be the source’s alphabet Q. In general, the sets of basis states in the decomposition of a two-qudit density matrix may differ, but we require a symmetric decomposition such that the basis states of both qudits are $Q^{\prime }$. From this decomposition, they can construct an HMCQS with an underlying Markov dynamic described by Equation (9). This HMCQS has $|Q^{\prime }|$ internal states and transition probabilities $T_{\sigma_{x},\sigma_{x^{\prime }}}=\mathrm{Pr}\left(|\psi_{x^{\prime }}\rangle ||\psi_{x}\rangle \right)$ that can be calculated from $\rho_{0:2}$. Different separable decompositions of $\rho_{0:2}$ yield different HMCQSs, whose statistics over longer-length sequences may differ. Determining which is a more accurate model of the source requires performing tomography on $\rho_{0:3}$ to refine the model.

#### 6.7.3. l≥3

Finding a separable decomposition becomes more computationally expensive as the number of qudits increases [60]. Nevertheless, if one obtains a separable decomposition of $\rho_{0:3}$ where the basis states for all 3 qudits are $Q^{\prime }$, then one can create a length-2 Markov approximation of the source. Each length-2 sequence of qudits in $Q^{\prime }$ corresponds to a different internal HMCQS state. Thus, it has $|Q^{\prime }{|}^{2}$ internal states, unless some length-2 sequences are forbidden. The transition probabilities take the form $T_{\sigma_{x_{0},x_{1}},\sigma_{x_{1},x_{2}}}=\mathrm{Pr}\left(|\psi_{x_{2}}\rangle ||\psi_{x_{0}}\rangle |\psi_{x_{1}}\rangle \right)$. Only transition probabilities that obey concatenation are nonzero.

One can continue in this manner, approximating the source given a separable decomposition of $\rho_{0:l}$, to obtain an HMCQS with $|Q^{\prime }{|}^{l-1}$ states or fewer if some sequences are forbidden. Each state corresponds to a word of length l−1, where the pure states composing the word are drawn from $Q^{\prime }$. The number of internal states in the model grows exponentially with *ℓ*, but not all of these states may lead to unique future predictions. If so, they can be combined without a loss of predictivity, as is done in classical computational mechanics. A general algorithm for doing so is beyond the present scope.

### 6.8. Discussion

The preceding section detailed many aspects of identifying an unknown quantum process by tomographically reconstructing the length-*ℓ* density matrices $\rho_{0:l}$. When correlations exist between qudits, this provides a predictive advantage over the common assumption that sources are i.i.d.. Starting with the method for reconstructing classical processes, we developed a variety of measurement protocols for different values of *d*, |Q|, and *ℓ* to find a process’s statistics when Q is known. We then introduced effective models for quantum sources derived entirely from separable decompositions of the density matrices $\rho_{0:l}$ reconstructed via tomography.

Since our aim is to harness correlations to improve predictions of future measurement outcomes, it is worth asking, how accurately can future measurement outcomes be predicted? To begin to answer this, the following now defines maximally predictive measurements for the special case of l=2 and for arbitrary *ℓ*.

For a correlated qudit process, an observer with knowledge of the length-2 density matrix $\rho_{0:2}$ may perform a measurement on the first qudit and condition upon the outcome to reduce their uncertainty when measuring the second qudit. They apply $M_{0}$ (with possible outcomes $y_{i}$) on the first qubit $\rho_{0}$. This leaves the joint system in the classical quantum state:

$$\begin{array}{c} \rho_{0:2}^{M_{0}}=\sum_{y_{i}}\mathrm{Pr}\left(y_{i}\right)|y_{i}\rangle \langle y_{i}|⊗\rho_{1}^{y_{i}}\;, \end{array}$$

where the $\rho_{1}^{y_{i}}$ are the qudit density matrices conditioned on outcome $y_{i}$.

The conditional von Neumann entropy of the second qubit is then

$$\begin{array}{c} S\left(\rho_{1}|M_{0}\left(\rho_{0}\right)\right)=\sum_{y_{i}}\mathrm{Pr}\left(y_{i}\right)S\left(\rho_{1}^{y_{i}}\right)\;. \end{array}$$

The rank-one measurement with the minimal uncertainty in measurement outcomes is in the eigenbasis of $\rho_{1}^{y_{i}}$. Different $y_{i}$ values generally correspond to different minimal-entropy measurements on the second qubit.

For two qubits, we can find the PVM for which the conditional von Neumann entropy of the second qubit is minimized. This leads to a basis-independent property of the process:

$$\begin{array}{c} S_{min}\left(\rho_{1}|M_{min}\left(\rho_{0}\right)\right)=\min_{M_{0}}S\left(\rho_{1}|M_{0}\left(\rho_{0}\right)\right)\;, \end{array}$$

where the minimum is taken over all PVMs on $\rho_{0}$.

If an experimenter reconstructs $\rho_{0:l}$, a measurement on all but the last qudit in the block with a measurement protocol M with possible outcomes $y_{0:l-1}$ leaves the block in the classical-quantum state:

$$\begin{array}{c} \rho_{0:l}^{M}=\sum_{y_{0:l-1}}\mathrm{Pr}\left(y_{0:l-1}\right)|y_{0:l-1}\rangle \langle y_{0:l-1}|⊗\rho_{l}^{y_{0:l-1}}\;. \end{array}$$

To minimize the conditional von Neumann entropy of the *ℓ*th qubit, we calculate

$$\begin{array}{c} S_{min}\left(\rho_{l}|M_{min}\left(\rho_{0:l-1}\right)\right)=\min_{M}S\left(\rho_{l}|M\left(\rho_{0}\right)\right)\;, \end{array}$$

where the minimum is taken over all local measurement protocols on $\rho_{0:l-1}$.

Further development necessitates exploring the space of measurement protocols to find those with the greatest predictive advantage over i.i.d. models for arbitrary separable qudit processes.

Finally, we note that our procedure for source reconstruction requires a number of model states that grows exponentially with *ℓ*. Future work on finding *minimal* models from density matrices will also require combining states with identical predictions and performing inference over possible model topologies, as with classical Bayesian Structural Inference.

## 7. Conclusions

Inspired by prior information-theoretic studies of classical stochastic processes, we introduced methods to quantify structure and information production for stationary quantum information sources. We identified properties related to the quantum block entropy S(l) that allow one to determine the amount of randomness and structure within a given qudit process. We gave bounds on informational properties of the resulting measured classical processes. In particular, we showed that they cannot have a lower entropy rate or block entropy (at any *ℓ*) than the original quantum process.

We analyzed a number of hidden Markov chain quantum sources (HMCQSs), explaining how an observer synchronizes to a source’s internal states via measuring the emitted qudits. If the source allows for synchronizing observations, then we showed that adaptive measurement protocols are capable of synchronizing and maintaining synchronization when fixed-basis measurements cannot.

Sequels will extend these methods and results in a number of ways. Despite focusing here on separable quantum sequences for simplicity, entangled qudit sequences can similarly be studied by combining an HMCQS and a *D*-dimensional quantum system capable of sequentially generating matrix product states [24]. Doing so will open up the study of entropy convergence of matrix product operators [61].

Many results exist for classical stochastic processes that may be extended to quantum processes. For example, there exist closed-form expressions for informational measures for nondiagonalizable classical dynamics [50,62,63,64]. Extending these to quantum dynamics would allow for more accurate determination of the quantum information properties introduced here. Similarly, the preceding lays the groundwork for fluctuation theorems and large deviation theories of separable quantum processes. Finally, it will be worthwhile to develop a causal equivalence relation for quantum stochastic processes and develop quantum ε-machines by extending classical results [65].

Separable quantum sequences also serve as a resource for information processing by finite-state quantum information transducers that transform one quantum process to another. Beyond interest in their own right, such operations have thermodynamic consequences, either requiring work to operate (as overcoming dissipation induced by Landauer erasure [66]) or acting as a quantum version of information-powered engines capable of leveraging environmental correlations to perform useful work [26,67,68]. This behavior has already been demonstrated for certain quantum processes [69].

Finally, spatially extended, ground, and thermal states of spin chains under various Hamiltonians are quantum processes. And so, the quantum information measures introduced here can serve to classify these states according to the source complexity required to generate them. 

## Data Availability

The raw data supporting the conclusions of this article will be made available by the authors on request.

## Appendix A. Information in Classical Processes

This section briefly recounts properties that quantify the randomness and correlation of classical stochastic processes as developed in Ref. [11]. Details and complete proofs can be found there. The main text here introduces versions appropriate to quantum processes.

### Appendix A.1. Shannon Entropy

We quantify the amount of uncertainty in a discrete random variable *X* with possible outcomes $\left\{x_{1},x_{2},\ldots x_{n}\right\}$ by its *Shannon entropy*: [7]:

$$\begin{array}{c} H\left[X\right]\equiv -\sum_{i=1}^{n}\mathrm{Pr}\left(x_{i}\right)\log_{2}\mathrm{Pr}\left(x_{i}\right)\;. \end{array}$$

We use $\log_{2}$, in which case the units for Shannon entropy are *bits*.

To study correlations between multiple random variables, we use several additional information quantities related to the Shannon entropy. First, the *joint entropy* of two discrete random variables—*X* and *Y*, with possible outcomes $\left\{x_{1},x_{2},\ldots x_{n}\right\}$ and $\left\{y_{1},y_{2},\ldots y_{m}\right\}$, respectively—is defined as

$$\begin{array}{c} H\left[X,Y\right]\equiv -\sum_{i=1}^{n}\sum_{j=1}^{m}\mathrm{Pr}\left(x_{i},y_{j}\right)\log_{2}\mathrm{Pr}\left(x_{i},y_{j}\right)\;. \end{array}$$

*X* and *Y* are statistically independent if and only if the joint entropy decomposes as H[X,Y]=H[X]+H[Y].

Second, the *conditional entropy* of *X* conditioned on *Y* is

$$\begin{array}{c} H\left[X|Y\right]\equiv -\sum_{i=1}^{n}\sum_{j=1}^{m}\mathrm{Pr}\left(x_{i},y_{j}\right)\log_{2}\mathrm{Pr}\left(x_{i}|y_{j}\right)\;. \end{array}$$

H[X|Y] is the uncertainty in the value of *X* after already knowing the value of *Y*. Note that H[X|Y]≥0 and is not symmetric: H[X|Y]≠H[Y|X]. If *X* and *Y* are uncorrelated, then H[X|Y]=H[X].

From the above definitions, one can derive the following identity, linking conditional and joint entropies:

$$\begin{array}{c} H\left[X|Y\right]=H\left[X,Y\right]-H\left[Y\right]\;. \end{array}$$

Third and finally, the *mutual information* between two random variables is

$$\begin{array}{c} I\left[X:Y\right]\equiv H\left[X\right]-H\left[X|Y\right]\;. \end{array}$$

This is the amount of information one can gain about *X* by having complete knowledge of *Y*. The mutual information is symmetric, and I[X:Y]=0 if and only if *X* and *Y* are statistically independent.

### Appendix A.2. Block Entropy

For a classical stochastic process, we quantify the amount of uncertainty in a block of *ℓ* consecutive random variables by taking the joint entropy $H\left[X_{0:l}\right]$. This is the *block entropy*:

$$\begin{array}{cc} H\left[l\right] & \equiv H\left[X_{0:l}\right]\\ \; & =-\sum_{x_{0:l}}\mathrm{Pr}\left(x_{0:l}\right)\log_{2}\mathrm{Pr}\left(x_{0:l}\right)\;, \end{array}$$

where the sum is taken over all words of length *ℓ* and H[0]≡0.

H[l] is monotonically increasing and concave down, and its behavior as l→∞ is indicative of a process’s correlations and randomness [11]. The generic behavior of H[l] and its relation to other information properties that we will define can be seen in Figure 2.

### Appendix A.3. Shannon Entropy Rate

The following briefly summarizes Ref. [11]’s results. Refer there for a more thorough exploration of information-theoretic quantities related to multivariate systems and the block entropy.

First among these is the *Shannon entropy rate* $h_{\mu }$:

(A1) $$\begin{array}{c} h_{\mu }=\lim_{l\to \infty }\frac{H\left[l\right]}{l}\;. \end{array}$$

The limit in Equation (A1) is guaranteed to exist for all stationary processes [70]. $h_{\mu }$ is irreducible randomness produced by an information source. Its units are *bits per symbol*.

The Shannon entropy rate can equivalently be written using the *conditional entropy*:

(A2) $$\begin{array}{c} h_{\mu }=\lim_{l\to \infty }H\left[X_{0}|X_{-l:0}\right]\;, \end{array}$$

Therefore, $h_{\mu }$ can equivalently be thought of as the average uncertainty in the next symbol if all preceding symbols are known.

To better appreciate $h_{\mu }$, we consider a few simple cases.

For an i.i.d. process, the block entropy trivially is $H\left[l\right]=lH\left[X_{0}\right]$, and therefore, $h_{\mu }=H\left[X_{0}\right]$. Since there are no correlations between variables, knowledge of past symbols cannot reduce the uncertainty of the next.

If a process is periodic (for example, consisting of alternating 0s and 1s), then a keen observer will note this pattern and be able to predict with certainty all future symbols of the process. In this case, $h_{\mu }=0$.

For stationary Markov and hidden Markov processes, an observer can leverage past observations to reduce their uncertainty about succeeding symbols. $h_{\mu }$ will be their average uncertainty about the next symbol once they have accounted for all of the correlations with past symbols.

Graphically, $h_{\mu }$ corresponds to the slope of the block entropy curve as l→∞, as shown in Figure 2.

### Appendix A.4. Redundancy

For a stochastic process with alphabet X, the maximum entropy rate is $\log_{2}\left|X\right|$, corresponding to i.i.d. random variables $X_{t}$ with uniform distributions over all measurement outcomes $x_{t}$. Any other stationary process can be *compressed* down to its entropy rate $h_{\mu }<\log_{2}\left|X\right|$.

The amount that a particular source can be compressed is known as its *redundancy*, which is defined as

$$\begin{array}{c} R\equiv \log_{2}\left|X\right|-h_{\mu }\;. \end{array}$$

R includes two very different effects: bias within individual random variables and correlations between different random variables. To determine the relative importance of those two factors requires closer examination of H[l].

### Appendix A.5. Block Entropy Derivatives and Integrals

Since the limit in Equation (A1) exists, H[l] scales (at most) linearly. We are interested in how H[l] converges to its linear asymptote, and we will see that taking discrete derivatives of H[l] (and integrals of those derivatives) provides us with useful quantities for classifying processes.

Consider applying a discrete derivative operator Δ to a function F:Z→R:

$$\begin{array}{c} \Delta F(l)=F(l)-F(l-1)\;. \end{array}$$

We can apply Δ to *F* multiple times to obtain higher-order derivatives:

$$\begin{array}{c} \Delta^{n}F\left(l\right)=\left(\Delta \circ \Delta^{n-1}\right)F\left(l\right)\;. \end{array}$$

Taking discrete derivatives of H[l] yields a set of functions $\Delta^{n}H\left[l\right]$. We then study how these discrete derivatives themselves converge to their asymptotic values. To do so, we take “integrals” of a discrete function ΔF(l) in the following manner:

(A3) $$\begin{array}{c} \sum_{l=A}^{B}\Delta F\left(l\right)=F\left(B\right)-F\left(A-1\right)\;. \end{array}$$

To study the convergence properties of each $\Delta^{n}H\left[l\right]$, we compare it at each *ℓ* to its asymptotic value $\lim_{l\to \infty }\Delta^{n}H\left[l\right]$. We do so with the following general integral form:

(A4) $$\begin{array}{c} I_{n}\equiv \sum_{l=l_{0}}^{\infty }\left[\Delta^{n}H\left(l\right)-\lim_{l\to \infty }\Delta^{n}H\left[l\right]\right]\;, \end{array}$$

where $l_{0}$ is the first value of *ℓ* for which $\Delta^{n}H\left[l\right]$ is defined.

### Appendix A.6. Entropy Gain

The first derivative of H[l] is known as the *entropy gain*. It is defined as

$$\begin{array}{c} \Delta H\left[l\right]\equiv H\left[l\right]-H\left[l-1\right]\;, \end{array}$$

for l>0. We set $\Delta H\left[0\right]\equiv \log_{2}\left(X\right)$. The entropy gain is the amount of additional uncertainty introduced by increasing the block size by one random variable, and its units are *bits per symbol*. Note that, because H[l] is monotone increasing and concave, ΔH[l]≥ΔH[l+1]≥0 for all *ℓ*. This behavior is shown in Figure 3.

The entropy gain can be rewritten as a conditional entropy:

(A5) $$\begin{array}{c} \Delta H\left[l\right]=H\left[X_{0}|X_{-l:0}\right]\;, \end{array}$$

in which case its relation to the entropy rate becomes clear. Using Equation (A2), we see that

$$\begin{array}{c} h_{\mu }=\lim_{l\to \infty }\Delta H\left[l\right]\;. \end{array}$$

For an observer with no prior knowledge of an information source, it will often be necessary to estimate the entropy rate of a source using finite sequences of data. In this case, the entropy gain can serve as a finite-*ℓ* approximation of the true entropy rate:

(A6) $$\begin{array}{cc} h_{\mu }\left(l\right) & \equiv \Delta H\left[l\right]\\ \; & \equiv H\left[l\right]-H\left[l-1\right]\;. \end{array}$$

### Appendix A.7. Predictability Gain

The second derivative of H[l] is the *predictability gain*, which is defined as

$$\begin{array}{cc} \Delta^{2}H\left[l\right] & \equiv \Delta h_{\mu }\left(l\right)\\ \; & =h_{\mu }\left(l\right)-h_{\mu }\left(l-1\right)\;, \end{array}$$

where l>0. Note that $\Delta^{2}H\left[l\right]\leq 0$.

$|\Delta^{2}H\left[l\right]|$ is the average amount of additional predictive information an observer obtains when expanding their observations from blocks of length l−1 to blocks of length *ℓ*, and its units are *bits per symbol^2^*. Large values of $|\Delta^{2}H\left[l\right]|$ imply that the *ℓ*th measurement is particularly informative to an observer and therefore greatly improves their estimate of the entropy rate, as given by Equation (A6).

One can also calculate higher-order discrete derivatives of H[l]. For our purposes, this is not necessary except to note that, for stationary processes,

$$\begin{array}{c} \lim_{l\to \infty }\Delta^{n}H\left[l\right]=0,n\geq 2\;. \end{array}$$

For n=2, this follows from convergence of $h_{\mu }\left(l\right)$, and the argument for all n>2 is similar.

### Appendix A.8. Total Predictability

We can now integrate the functions $\Delta^{n}H\left[l\right]$. As a general heuristic, the larger the magnitude of these integrals, the more correlation or statistical bias exists within the process.

We begin by studying how the predictability gain $\Delta^{n}H\left[l\right]$ converges to its asymptotic value $\lim_{l\to \infty }\Delta^{2}H\left[l\right]=0$. Since $\Delta^{2}H\left[0\right]$ is undefined, we will integrate using Equation (A4) with $l_{0}=1$ to obtain the total predictability G:

(A7) $$\begin{array}{c} G\equiv I_{2}=\sum_{l=1}^{\infty }\Delta^{2}H\left(l\right)\;. \end{array}$$

Since $\Delta^{2}H\left[l\right]<0$ for all *ℓ*, G<0 as well. The units of G are *bits per symbol*. Graphically, it is the area between the predictability gain curve and its linear asymptote of 0, as seen in Figure 4.

To interpret G’s value, we apply Equation (A3) to get

$$\begin{array}{cc} G & =-\Delta H\left[0\right]+\lim_{l\to \infty }\Delta H\left[l\right]\\ \; & =-\log_{2}\left(|X|\right)+h_{\mu }\\ \; & =-R\;. \end{array}$$

A process’s total predictability is then equal in magnitude to its redundancy, and we can interpret |G| as the amount of predictable information per symbol for a process. Here, we emphasize once more that for a given process with alphabet X, any random variable has a maximum entropy of $\log_{2}\left(|X|\right)$ that consists of two kinds of information: $h_{\mu }$, the irreducible randomness, and |G|, the amount of information that an observer can possibly predict about it.

The total predictability is thus a function of the entropy rate for a given process and shares $h_{\mu }$’s weakness: It cannot identify statistical correlations between random variables. A large value of |G| could be the result of either an i.i.d. process with heavily biased random variables or strong correlations between subsequent variables. Fortunately, our next quantity does distinguish between these cases.

### Appendix A.9. Excess Entropy

Investigating the convergence of the entropy gain to the asymptotic entropy rate $h_{\mu }$ leads to a well-studied process property, the excess entropy:

$$\begin{array}{cc} E\equiv I_{1} & =\sum_{l=1}^{\infty }\left[\Delta H\left[l\right]-\lim_{l\to \infty }\Delta H\left[l\right]\right]\\ \; & =\sum_{l=1}^{\infty }\left[\Delta H\left[l\right]-h_{\mu }\right]\;. \end{array}$$

Since $\Delta H\left[l\right]\geq h_{\mu }$, E≥0 and its units are *bits*.

Our interpretation of E becomes clearer after applying Equation (A3) to obtain

(A8) $$\begin{array}{c} E=\lim_{l\to \infty }\left[H\left[l\right]-h_{\mu }l\right]\;. \end{array}$$

While $h_{\mu }$ determines the linear asymptotic behavior of H[l], E encapsulates all sublinear effects. We largely discuss processes for which E is finite, known as *finitary* processes. A process for which E is infinite (for example, if H[l] scales logarithmically with *ℓ*), is known as an *infinitary* process. Graphically, E corresponds to the l=0 intercept of the linear asymptote to the block entropy curve, as shown in Figure 2, as well as the area between the area between the entropy gain curve and its asymptote $h_{\mu }$, as seen in Figure 3.

The excess entropy can also be written as a mutual information between two halves of a process’ chain of random variables:

(A9) $$\begin{array}{c} E=\lim_{l\to \infty }I\left[X_{-l:0}:X_{0:l}\right]\;. \end{array}$$

This suggests it is the total amount of information in a process’s past that is useful for predicting the future. It is therefore considered an indicator of the amount of process *memory*. A more structural approach reveals that E is a lower bound on the actual amount of memory required to predict a stochastic process [65].

Importantly, E easily distinguishes between i.i.d. processes (for which E=0) and processes with correlations between random variables (E>0). For all processes that are periodic with period *p*, $h_{\mu }=0$, and H[l] reaches a maximum value of $\log_{2}p$ for l=p. Therefore, all period-*p* processes have $E=\log_{2}p$.

As with $h_{\mu }$, it is often useful for an observer to make an approximation of the true excess entropy using only finite length-*ℓ* symbol sequences. Using Equation (A8), we can estimate E as

(A10) $$\begin{array}{cc} E(l) & \equiv H\left[l\right]-lh_{\mu }\left(l\right)\;. \end{array}$$

### Appendix A.10. Transient Information

Based upon our analysis of the excess entropy, we can now say that as l→∞, the block entropy curve has a linear asymptote defined as

(A11) $$\begin{array}{c} H\left[l\right]∼E+h_{\mu }l\;. \end{array}$$

We capture the way that H[l] converges to this asymptote by taking another discrete integral to obtain the transient information:

$$\begin{array}{c} T\equiv -I_{0}=\sum_{l=0}^{\infty }\left[E+h_{\mu }l-H\left[l\right]\right]\;. \end{array}$$

The units of T are *bits × symbol*. T is represented graphically in Figure 2 as the area between the H[l] curve and its linear asymptote for l→∞.

The transient information can be rewritten as

$$\begin{array}{c} T=\sum_{l=1}^{\infty }l\left[h_{\mu }\left(l\right)-h_{\mu }\right]\;, \end{array}$$

indicating that T is a measure of how difficult it is to *synchronize* to a process. An observer is considered “synchronized” when they are able to infer exactly which internal state the source currently occupies. If they remain synchronized, then they can optimally predict future measurement outcomes. That is, their estimate of the source’s entropy rate $h_{\mu }\left(l\right)$ is equal to its actual entropy rate $h_{\mu }$.

T is a notable quantity since, unlike E, it is capable of distinguishing between different period-*p* processes.

### Appendix A.11. Markov Order

The final property we introduce for classical stochastic processes is the Markov order. A process has Markov order *R* if *R* is the minimum value for which the following condition holds:

$$\begin{array}{c} \mathrm{Pr}\left(X_{0}|X_{R:0}\right)=\mathrm{Pr}\left(X_{0}|X_{-\infty :0}\right)\;. \end{array}$$

From Equation (2), we conclude that a Markov process is one for which R≤1. More generally, for a process with Markov order *R*, the distribution for $X_{t}$ depends only on the previous *R* symbols. Equivalently, *R* is the value of *ℓ* for which the block entropy reaches its linear asymptote:

$$\begin{array}{c} H\left[R\right]=E+h_{\mu }R\;. \end{array}$$

This fact is represented graphically in Figure 2.

The overwhelming majority of finite-state hidden Markov processes have a infinite Markov order, meaning that $H\left[l\right]<E+h_{\mu }l$ for all *ℓ* [71]. Nevertheless, an observer can still make use of finite-*ℓ* estimates of the information properties defined here. In fact, these estimates typically converge exponentially fast with *ℓ* [72].

## Appendix B. Quantum Channels for Preparation and Measurement

The main text claims that a separable qudit process is the result of passing realizations of a classical process $\overset{↔}{X}$ with symbol alphabet X through a classical quantum channel that takes $x\in X\to |\psi_{x}\rangle \in H$. It also claims that the act of measurement can be described as a quantum-classical channel taking $\rho_{0:l}\to y_{0:l}$. The following elaborates on both claims, adopting the formalism of Ref. [6].

We first describe passing realizations of a classical process $\overset{↔}{X}$ through a *conditional quantum encoder*. Consider a classical quantum state composed of a classical register of dimension |X| and a qudit in H initialized in the state |0〉. Furthermore, let the classical register store the outcome of some classical random variable $X_{t}$ with outcomes x∈X such that

(A12) $$\begin{array}{c} \rho_{XA}=\sum_{x}\mathrm{Pr}\left(x\right)|x\rangle {\langle x|}_{X}⊗|0\rangle {\langle 0|}_{A}\;. \end{array}$$

This state will serve as input to a conditional quantum encoder $E_{XA\to B}$ that consists of a set $\left\{E_{A\to B}^{x}\right\}$ of |X| completely positive trace-preserving (CPTP) maps. We construct each map such that it transforms the initial quantum state |0〉 to the desired pure qudit state $|\psi_{x}\rangle$, i.e.,

(A13) $$\begin{array}{c} E_{A\to B}^{x}\left(|0\rangle {\langle 0|}_{A}\right)=|\psi_{x}\rangle {\langle \psi_{x}|}_{B}\;, \end{array}$$

with $E_{A\to B}^{x}$ being a unitary operation. (For d=2, we can consider rotations on the Bloch sphere).

The encoding is

$$\begin{array}{cc} \rho_{B} & =E_{XA\to B}\left(\rho_{XA}\right)\\ \; & =tr_{X}\left(\sum_{x}\mathrm{Pr}\left(x\right)|x\rangle \langle x|⊗E_{A\to B}^{x}\left(|0\rangle \langle 0|\right)\right)\\ \; & =\sum_{x}\mathrm{Pr}\left(x\right)|\psi_{x}\rangle {\langle \psi_{x}|}_{B}\;. \end{array}$$

Now, taking *ℓ* classical registers that consist of length-*ℓ* words $x_{0:l}$ of a classical process $\overset{↔}{X}$, we can use $E_{XA\to B}$ to encode $X_{0:l}$ and obtain

$$\begin{array}{cc} \rho_{0:l} & =tr_{X_{0:l}}\left(\sum_{x_{0:l}}\mathrm{Pr}\left(x_{0:l}\right)\prod_{t=0}^{l-1}|x_{t}\rangle \langle x_{t}|⊗E_{A\to B}^{x_{t}}\left(|0\rangle \langle 0|\right)\right)\\ \; & =\sum_{w}\mathrm{Pr}\left(w\right)|\psi_{w}\rangle \langle \psi_{w}|\;, \end{array}$$

where $|\psi_{w}\rangle$ take the separable form of Equation (5), and $\rho_{0:l}$ therefore matches Equation (6).

The measurement of qudits is described similarly. Let *M* be a POVM with elements $\left\{E_{y}\right\}$ that acts on a qudit state ρ in such a way that it records each measurement outcome in a classical register *Y*. The distribution over values in *Y* is determined by

$$\begin{array}{cc} Y & =M(\rho )\\ \; & =\sum_{y}tr\left(E_{y}\rho \right)|y\rangle \langle y|\;. \end{array}$$

Likewise, consider $M_{0:l}$ to be a length-*ℓ* sequence of measurements with possible measurement outcome sequences $y_{0:l}$ determined according to some protocol M. When applying $M_{0:l}$ to *ℓ* consecutive qudits in the joint state $\rho_{0:l}$, we assume that $M_{0:l}$ consists of local measurements in the form of Equation (10). In this case, we factor the POVM elements corresponding to particular sequences of measurement outcomes: $E_{y_{0:l}}={⨂}_{t=0}^{l-1}E_{y_{t}}$. A length-*ℓ* measurement outcome can be stored in $Y_{0:l}$, a set of *ℓ* classical registers, with its associated probability $tr(E_{y_{0:l}}\rho_{0:l})$ so that

$$\begin{array}{cc} Y_{0:l} & =M_{0:l}\left(\rho_{0:l}\right)\\ \; & =\sum_{y_{0:l}}tr\left(E_{y_{0:l}}\rho_{0:l}\right)|y_{0:l}\rangle \langle y_{0:l}|\\ \; & =\sum_{y_{0:l}}tr\left(\overset{l-1}{\underset{t=0}{⨂}}E_{y_{t}}\rho_{0:l}\right)|y_{0:l}\rangle \langle y_{0:l}|\;, \end{array}$$

where the last line assumes local measurements. Each $|y_{0:l}\rangle$ is then a separable state, and all $|y_{0:l}\rangle$ are orthogonal.

## References

[1] Heisenberg W. (1927). Über den anschaulichen inhalt der quantentheoretischen kinematik und mechanik. Z. Phys..

[2] Peres A. (1991). Two simple proofs of the Kochen-Specker theorem. J. Phys. A.

[3] Wootters W.K., Fields B.D. (1989). Optimal state-determination by mutually unbiased measurements. Ann. Phys..

[4] Renes J.M., Blume-Kohout R., Scott A.J., Caves C.M. (2004). Symmetric informationally complete quantum measurements. J. Math. Phys..

[5] von Neumann J. (2018). Mathematical Foundations of Quantum Mechanics: New Edition.

[6] Wilde M. (2017). Quantum Information Theory.

[7] Shannon C.E. (1948). A mathematical theory of communication. Bell Sys. Tech. J..

[8] Schumacher B. (1995). Quantum coding. Phys. Rev. A.

[9] Crutchfield J.P. (2012). Between order and chaos. Nat. Phys..

[10] Crutchfield J.P., Young K. (1989). Inferring statistical complexity. Phys. Rev. Let..

[11] Crutchfield J.P., Feldman D.P. (2003). Regularities unseen, randomness observed: Levels of entropy convergence. Chaos.

[12] Travers N.F., Crutchfield J.P. (2011). Exact synchronization for finite-state sources. J. Stat. Phys..

[13] Travers N.F., Crutchfield J.P. (2011). Asymptotic synchronization for finite-state sources. J. Stat. Phys..

[14] Venegas-Li A.E., Jurgens A.M., Crutchfield J.P. (2020). Measurement-induced randomness and structure in controlled qubit processes. Phys. Rev. E.

[15] Holevo A.S. (1998). Quantum coding theorems. Usp. Mat. Nauk.

[16] Datta N., Suhov Y. (2002). Data Compression Limit for an Information Source of Interacting Qubits. Quantum Inf. Process..

[17] Petz D., Mosonyi M. (2001). Stationary quantum source coding. J. Math. Phys..

[18] Nagamatsu Y., Mizutani A., Ikuta R., Yamamoto T., Imoto N., Tamaki K. (2016). Security of quantum key distribution with light sources that are not independently and identically distributed. Phys. Rev. A.

[19] Pollock F.A., Rodriguez-Rosario C., Frauenheim T., Paternostro M., Modi K. (2018). Operational Markov condition for quantum processes. Phys. Rev. Lett..

[20] Pollock F.A., Rodriguez-Rosario C., Frauenheim T., Paternostro M., Modi K. (2018). Non-Markovian quantum processes: Complete framework and efficient characterization. Phys. Rev. A.

[21] Taranto P., Pollock F.A., Milz S., Tomamichel M., Modi K. (2019). Quantum Markov order. Phys. Rev. Lett..

[22] Taranto P., Pollock F.A., Modi K. (2021). Non-Markovian memory strength bounds quantum process recoverability. npj Quantum Inf..

[23] Taranto P., Milz S., Pollock F.A., Modi K. (2019). Structure of quantum stochastic processes with finite Markov order. Phys. Rev. A.

[24] Schon C., Solano E., Verstraete F., Cirac J.I., Wolf M.M. (2005). Sequential generation of entangled multiqubit states. Phys. Rev. Lett..

[25] Schon C., Hammerer K., Wolf M.M., Cirac J.O., Solano E. (2007). Sequential generation of matrix-product states in cavity QED. Phys. Rev. A.

[26] Boyd A.B., Mandal D., Crutchfield J.P. (2017). Correlation-powered information engines and the thermodynamics of self-correction. Phys. Rev. E.

[27] Chapman A., Miyake A. (2015). How an autonomous quantum Maxwell demon can harness correlated information. Phys. Rev. E.

[28] Gu M., Wiesner K., Rieper E., Vedral V. (2012). Occam’s quantum razor: How quantum mechanics can reduce the complexity of classical models. Nat. Commun..

[29] Mahoney J.R., Aghamohammadi C., Crutchfield J.P. (2016). Occam’s quantum strop: Synchronizing and compressing classical cryptic processes via a quantum channel. Sci. Rep..

[30] Suen W.Y., Elliot T.J., Thompson J., Garner A.J., Mahoney J.R., Vedral V., Gu M. (2022). Surveying structural complexity in quantum many-body systems. J. Stat. Phys..

[31] Binder F.C., Thompson J., Gu M. (2018). Practical unitary simulator for non-Markovian complex processes. Phys. Rev. Lett..

[32] Loomis S.P., Crutchfield J.P. (2019). Strong and Weak Optimizations in Classical and Quantum Models of Stochastic Processes. J. Stat. Phys..

[33] Nielsen M.A., Chuang I.L. (2011). Quantum Computation and Quantum Information: 10th Anniversary Edition.

[34] Gudder S. (2008). Quantum Markov chains. J. Math. Phys..

[35] Monras A., Beige A., Wiesner K. (2010). Hidden Quantum Markov Models and non-adaptive read-out of many-body states. arXiv.

[36] Wiesner K., Crutchfield J.P. (2008). Computation in finitary stochastic and quantum processes. Phys. D.

[37] Srinivasan S., Gordon G., Boots B. Learning hidden quantum Markov models. Proceedings of the International Conference on Artificial Intelligence and Statistics.

[38] Perez-Garcia D., Verstraete F., Wolf M.M., Cirac J.I. (2006). Matrix product state representations. arXiv.

[39] Verstraete F., García-Ripoll J.J., Cirac J.I. (2004). Matrix product density operators: Simulation of finite-temperature and dissipative systems. Phys. Rev. Lett..

[40] Moore C., Crutchfield J.P. (2000). Quantum automata and quantum grammars. Theoret. Comp. Sci..

[41] Qiu D., Li L., Mateus P., Gruska J. (2012). Quantum Finite Automata. Handbook of Finite State Based Models and Applications.

[42] Zheng S., Qiu D., Li L., Gruska J. (2012). One-way finite automata with quantum and classical states. Lecture Notes in Computer Science.

[43] Junge M., Renner R., Sutter D., Wilde M.M., Winter A. (2018). Universal recovery maps and approximate sufficiency of quantum relative entropy. Ann. Henri Poincaré.

[44] Lanford O.E., Robinson D.W. (1968). Mean entropy of states in quantum-statistical mechanics. J. Math. Phys..

[45] Ohya M., Petz D. (1993). Quantum Entropy and Its Use.

[46] Cover T.M., Thomas J.A. (1991). Elements of Information Theory.

[47] Travers N.F., Crutchfield J.P. (2011). Equivalence of history and generator *ϵ*-machines. arXiv.

[48] Ellison C.J., Mahoney J.R., Crutchfield J.P. (2009). Prediction, retrodiction, and the amount of information stored in the present. J. Stat. Phys..

[49] Jurgens A.M., Crutchfield J.P. (2021). Shannon entropy rate of hidden Markov processes. J. Stat. Phys..

[50] Crutchfield J.P., Riechers P., Ellison C.J. (2016). Exact complexity: Spectral decomposition of intrinsic computation. Phys. Lett. A.

[51] Marzen S., Crutchfield J.P. (2015). Informational and causal architecture of discrete-time renewal processes. Entropy.

[52] Jurgens A., Crutchfield J.P. (2021). Divergent predictive states: The statistical complexity dimension of stationary, ergodic hidden Markov processes. Chaos.

[53] Fujiwara Y. (2013). Parsing a sequence of qubits. IEEE Trans. Inf. Theory.

[54] Strelioff C.C., Crutchfield J.P. (2014). Bayesian structural inference for hidden processes. Phys. Rev. E.

[55] Thew R.T., Nemoto K., White A.G., Munro W.J. (2002). Qudit quantum-state tomography. Phys. Rev. A.

[56] Bengtsson I., Życzkowski K. (2017). Geometry of Quantum States: An Introduction to Quantum entanglement.

[57] Lawrence J., Brukner Č., Zeilinger A. (2002). Mutually unbiased binary observable sets on n qubits. Phys. Rev. A.

[58] Gharibian S. (2008). Strong NP-hardness of the quantum separability problem. arXiv.

[59] Horodecki P. (1997). Separability criterion and inseparable mixed states with positive partial transposition. Phys. Lett. A.

[60] Horodecki M., Horodecki P., Horodecki R. (2001). Separability of n-particle mixed states: Necessary and sufficient conditions in terms of linear maps. Phys. Lett. A.

[61] Pirvu B., Murg V., Cirac J.I., Verstraete F. (2010). Matrix product operator representations. New J. Phys..

[62] Riechers P., Crutchfield J.P. (2018). Spectral simplicity of apparent complexity, Part I: The nondiagonalizable metadynamics of prediction. Chaos.

[63] Riechers P.M., Crutchfield J.P. (2018). Beyond the spectral theorem: Decomposing arbitrary functions of nondiagonalizable operators. AIP Adv..

[64] Riechers P., Crutchfield J.P. (2018). Spectral simplicity of apparent complexity, Part II: Exact complexities and complexity spectra. Chaos.

[65] Shalizi C.R., Crutchfield J.P. (2001). Computational mechanics: Pattern and prediction, structure and simplicity. J. Stat. Phys..

[66] Landauer R. (1961). Irreversibility and heat generation in the computing process. IBM J. Res. Dev..

[67] Boyd A.B., Mandal D., Crutchfield J.P. (2016). Leveraging environmental correlations: The thermodynamics of requisite variety. J. Stat. Phys..

[68] Jurgens A.M., Crutchfield J.P. (2020). Functional thermodynamics of Maxwellian ratchets: Constructing and deconstructing patterns, randomizing and derandomizing behaviors. Phys. Rev. Res..

[69] Huang R., Riechers P., Gu M., Narasimhachar V. (2022). Engines for predictive work extraction from memoryfull quantum stochastic processes. arXiv.

[70] Cover T.M., Thomas J.A. (2006). Elements of Information Theory.

[71] James R.G., Mahoney J.R., Ellison C.J., Crutchfield J.P. (2014). Many roads to synchrony: Natural time scales and their algorithms. Phys. Rev. E.

[72] Travers N.F. (2014). Exponential bounds for convergence of entropy rate approximations in hidden Markov models satisfying a path-mergeability condition. Stoch. Proc. Appl..

